# *Achog1* is required for the asexual sporulation, stress responses and pigmentation of *Aspergillus cristatus*

**DOI:** 10.3389/fmicb.2022.1003244

**Published:** 2022-11-25

**Authors:** Lei Shao, Yumei Tan, Shiying Song, Yuchen Wang, Yongxiang Liu, Yonghui Huang, Xiyi Ren, Zuoyi Liu

**Affiliations:** ^1^College of Agriculture, Guizhou University, Guiyang, China; ^2^Guizhou Key Laboratory of Agricultural Biotechnology, Guiyang, China; ^3^Institute of Biotechnology, Guizhou Academy of Agricultural Sciences, Guiyang, China; ^4^Innovative Institute for Plant Health, Zhongkai University of Agriculture and Engineering, Guangzhou, China

**Keywords:** pull down, LC–MS/MS, asexual sporulation, stress response, RNA-seq

## Abstract

*Aspergillus cristatus* is the dominant fungus during the fermentation of Fuzhuan brick tea; hypotonic conditions only induce its sexual development to produce ascospores, while hypertonic conditions only induce its asexual development to produce conidia, indicating that osmotic stress can regulate spore production in *A*. *cristatus*. However, the underlying regulatory mechanism is unclear. In this study, the role of *Achog1*, which is homologous to *hog1* from *Saccharomyces cerevisiae*, in sporulation, different kinds of stress responses and pigment production was investigated. Deletion mutants of *Achog1* were obtained by homologous recombination. Phenotypic observations showed that the time required to produce conidia was delayed, and the number of conidia produced was significantly reduced in the deletion mutants of *Achog1* in hypertonic media, indicating that *Achog1* plays a positive role in asexual development. Stress sensitivity tests showed that Δ*Achog1* strains were sensitive to hyperosmolarity, and the order of the sensitivity of Δ*Achog1* to different osmotic regulators was 3 M sucrose >3 M NaCl >3 M sorbitol. Moreover, the deletion mutants were sensitive to high oxidative stress. pH sensitivity tests indicated that *Achog1* inhibited the growth of *A*. *cristatus* under alkaline stress. Additionally, pigmentation was decreased in the *Achog1* deletion mutants compared with the WT. All the above developmental defects were reversed by the reintroduction of the *Achog1* gene in Δ*Achog1*. Pull-down and LC–MS/MS analysis showed that the expression levels of proteins interacting with *Achog1* were significantly different under low and high osmotic stress, and proteins related to conidial development were present only in the cultures treated with hyperosmotic stress. Transcription profiling data showed that *Achog1* suppressed the expression of several genes related to asexual development, osmotic and oxidative stress resistance. On the basis of gene knockout, pull-down mass spectrometry and RNA-seq analyses, a regulatory pathway for *Achog1* was roughly identified in *A*. *cristatus*.

## Introduction

*Aspergillus cristatus* is the dominant fungus during the fermentation of Fuzhuan brick tea and is known as the “Golden Flower Fungus” because of its golden yellow cleistothecia (Mo et al., 2008; Tan et al., 2017). Studies have shown that *A*. *cristatus* has antitumor activity (Deng et al., 2007), bacteriostatic effects (Li et al., 2011), antioxidant activity (Ouyang et al., 2011) and digestive enzyme activity (Yang, 2005; Ding, 2012). *A*. *cristatus* is a homothallic filamentous fungus and performs both sexual and asexual reproduction. Unlike *Aspergillus nidulans*, light does not significantly affect the development of *A*. *cristatus* (Tan et al., 2018). Interestingly, hypotonic conditions can only induce sexual development, while hypertonic conditions can only induce asexual development. Thus, *A*. *cristatus* provides an excellent genetic system for studying the mechanism of sporulation of filamentous fungi.

The sexual development of other fungi in *Aspergillus* is mainly affected by light, temperature, nutrition, and pH. For example, a complex protein, VeA/VelB/LaeA, called “Velvet,” plays a central role in the regulation of sexual development under light and dark conditions (Bayram et al., 2008). The regulation of asexual development is mainly regulated by the core regulatory network composed of *brlA*, *abaA*, and *wetA*, and other genes are directly or indirectly regulated by this network (Han and Adams, 2001; Tao and Yu, 2011; Alkhayyat et al., 2015). *brlA* was mainly found in vesicle and pedicel but not in mycelia and conidia. *brlA* is located upstream of the central regulatory pathway, and the expression of several genes related to asexual development, such as *rodA*, is regulated by *brlA* (Twumasi et al., 2009). We have been trying to explore the functions of genes related to sporulation in *A*. *cristatus* since 2013. Many genes associated with sexual and asexual spore production have been reported, such as *flbA*, *preA*, and *AcndtA* (Wang et al., 2014; Xiang et al., 2020; Wang et al., 2021). Nevertheless, it is not clear that why *A*. *cristatus* can produce ascospores under hypotonic conditions and conidia under hypertonic conditions. We speculate that there is a certain association between osmotic stress and spore production in *A*. *cristatus*.

It is well known that the high osmolarity glycerol mitogen-activated protein kinase signal transduction pathway (HOG-MAPK, HOG) is the main way to adapt to changes in osmolarity in the environment. Hog1 (MAPK) is the core protein kinase in this pathway (Krantz et al., 2006). It was first reported in *S*. *cerevisiae*. Cells respond to increases in osmolarity in the extracellular environment by activating *hog1* (Wurgler-Murphy et al., 1997). *hog1* was also reported to be involved in osmotic stress regulation in other fungi. *osm1* is a functional homolog of *hog1*, the hyphal growth of Δ*osm1* is very sensitive to osmotic stress, and the hyphal morphology changes under hypertonic conditions in *Magnaporthe grisea* (Dixon et al., 1999; Xu, 2000). *Thhog1* regulates the hypertonic stress response in *Trichoderma harzianum* (Delgado-Jarana et al., 2006). In *A*. *fumigatus*, *sakA*, the homolog of *hog1*, could regulate conidial germination (Du et al., 2006). A similar phenomenon was found in *Botrytis*. *cinerea* (Segmüller et al., 2007; Heller et al., 2012). *hog1* also plays a role in the oxidative stress response (Calmes et al., 2015). *Cpmk1*, a homolog of *hog1*, can regulate the production of pigment in *Cryphonectria parasitica* (Park et al., 2004). The effect of *hog1* on PH stress has also been reported. The deletion mutant of *hog1* slowed down significantly in the medium with alkaline pH in *A*. *fumigatus* (Ma et al., 2012). In summary, *hog1* plays an important role in the stress response, asexual sporulation and pigment production in fungi.

Sequence alignment revealed a gene homologous to *hog1*, SI65_07698, in the *A*. *cristatus* genome database. In this study, SI65_07698 was named *Achog1* based on the results of a phylogenetic analysis. *Achog1* deletion and complementation strains were generated to verify the role of *Achog1*. Under different osmotic stress conditions, pull-down mass spectrometry was carried out, and RNA sequencing (RNA-seq) was performed on the Δ*Achog1* and WT strains to explore the connection between the HOG pathway and spore production of *A*. *cristatus*.

## Materials and methods

### Strains, culture conditions and morphological analyses

The WT strains of *A*. *cristatus* (CGMCC 7.193) used in this study were isolated from Fuzhuan brick tea produced by the Yiyang Tea Factory in Yiyang, China. The strains were grown on low-concentration sodium chloride solid MYA media (20 g of malt extract, 5 g of yeast extract, 30 g of sucrose, 50 g of sodium chloride, and 1,000 mL of water) at 28°C to induce sexual development and high-concentration sodium chloride solid media (20 g of malt extract, 5 g of yeast extract, 30 g of sucrose, 170 g of sodium chloride, and 1,000 mL of water) at 37°C to induce asexual development. SD/−Trp, SD/−Leu, SD/−Trp/−Leu, SD/−Trp/− Leu/-His and SD/−Trp/−Leu/-His/−Ade (purchased from Beijing Cool Lab Technology, China). The colonies were imaged using a Canon EOS 7D Mark II camera (Canon, Tokyo, Japan).

### Identification of proteins interacting with *AcHog1*

The *Achog1* gene fragment was ligated into the expression vector pGEX-6p-1, and GST fusion protein expression and transformation were performed. Then, the total proteins of *A*. *cristatus* cultivated under normal osmotic stress and hypertonic stress were extracted, and pull-down assays were carried out. The proteins were subsequently detected *via* LC–MS/MS. Proteins were verified by yeast two-hybrid assays. To facilitate subsequent analysis, the samples of *A*. *cristatus* cultivated under normal stress and hypertonic stress were named WT-D and WT-H, respectively.

### Phylogenetic analysis

Phylogenetic analysis was performed using MEGA 6.06 software with the conserved domains of homologous AcHog1 proteins (Tamura et al., 2013). ClustalW was employed for multiple sequence alignment with the default values. A maximum likelihood with a bootstrap value of 1,000 was used to generate a phylogenetic tree.

### Construction of the *Achog1* deletion strain (Δ*Achog1*) and complementation strain (Δ*Achog1-C*)

Homologous recombination was employed to delete the whole open reading frame (ORF) of *Achog1*. An *Achog1* deletion cassette containing *hph* as a selective marker was constructed *via* the fusion of the 5′-untranslated region (5’-UTR) and the 3′-untranslated region (3’-UTR). Briefly, the 5’-*BamH*I-*Xho*I UTR and 3’-*Spe*I-*Xba*I UTR were amplified using specific primer pairs (Supplementary Table S1) from genomic DNA of the WT strain and cloned into the pDHt/sk-hyg cloning sites to generate the final *Achog1*-L-pDHt/sk-hyg-*Achog1*-R knockout vector. To complement the *Achog1*-null mutant, the *Achog1* gene with its own promoter was amplified from the genomic DNA with the primer pair qc-*hog1*-F/R (Supplementary Table S1) and then inserted into pDHt/sknt at the enzyme sites *Hind*III*/Kpn*I.

The plasmids were confirmed *via* PCR, restriction enzyme digestion and sequencing. The knockout plasmids were transformed into the WT strains, and complementary plasmids were transformed into the Δ*Achog1* knockout strains by *Agrobacterium tumefaciens*-mediated transformation (ATMT), as previously described (Tan et al., 2018). The transformants were selected on MYA media that included 50 ug/mL hygromycin B or 80 ug/mL geneticin (G418) and were confirmed *via* PCR (the primers used are listed in Supplementary Table S1).

### Estimating transgene copy numbers *via* RT-PCR

The transgene copy number in Δ*Achog1* was estimated by using RT-PCR based on the methods of Song et al. (2002). Using the genomic DNA of Δ*veA*, a standard curve was established, in which the copy number was determined by Southern blot analysis (Tan et al., 2018). The genes encoding glyceraldehyde-3-phosphate dehydrogenase (*GAPDH*) and *HYG* were selected from the *A*. *cristatus* genome as candidate reference genes (the primers used are listed in Supplementary Table S1).

### Sample preparation for mRNA sequencing

RNA-seq was performed on the Δ*Achog1* and WT strains. A conidial suspension (concentration 1 × 10^6^ conidia/mL) was used for inoculation. Three biological replicates were grown on MYA media comprising 17% NaCl at 37°C. The WT strain produced a large number of conidial heads, but the Δ*Achog1* knockout strain did not after 39 h of culture. Based on these observations, all the samples were collected from the cellulose membrane, flash frozen in liquid nitrogen and stored at-80°C.

### Library preparation and sequencing

Total RNA was extracted as described previously using TRIzol (Gilbert et al., 2016). An Agilent Bioanalyzer 2,100 was subsequently used to assess the concentration and quality of the total RNA. mRNA libraries were prepared as described previously (Parkhomchuk et al., 2009). The quality of the final cDNA libraries was verified using an Agilent Bioanalyzer 2,100. The libraries were sequenced using an Illumina NovaSeq 6,000 (Illumina, United States). After filtering the raw data and checking the sequencing error rate and GC content distribution, the clean reads used for the subsequent analysis were obtained. HISAT2 software was used to quickly and accurately align the clean reads with the reference genome to obtain the positioning information. Differentially expressed genes were analysed using DESeq2 (Love et al., 2014); gene ontology (GO) functional enrichment and Kyoto Encyclopedia of Genes and Genomes (KEGG) pathway enrichment of the differentially expressed genes were performed by using ClusterProfiler software (Kanehisa and Goto, 2000; Young et al., 2010).

### RT-qPCR detection

Total RNA was extracted at the tested time point. Then, 2 mg of RNA was utilized to synthesize cDNA using a RevertAid First Strand cDNA Synthesis Kit (Thermo Fisher Scientific, catalogue#K1622). RT-qPCR was performed using a CFX96 Real-Time PCR Detection System (Bio-Rad Laboratories, Hercules CA, United States) in a total volume of 10 μL, which consisted of 5 μL of SsoFast EvaGreen SuperMix (Bio-Rad, catalogue# 172–5,201), 1 μL of each primer (10 pmol/ml) and 1 μL of template. *GAPDH* was selected as a candidate reference gene. The primers employed in the RT-qPCR experiments were designed using the Primer 3 online program,^1^ and the resulting RT-qPCR products were tested *via* agarose gel electrophoresis. Each primer pair was tested with serial dilutions of cDNA to determine the linear range of the RT-qPCR assays. Three biological replicates were analysed. All the RT-qPCR primers used are listed in Supplementary Table S1.

## Results

### Phylogenetic analysis of *Achog1* homologs and identification of the deletion strain Δ*Achog1* and complementation strain Δ*Achog1-C*

The homolog of *hog1*, SI65-07698, was identified based on the genome of *A*. *cristatus* (accession number [JXNT01000000]) (Ge et al., 2016). SI65_07698 is 1,689 bp in length, includes six introns and encodes a predicted protein of 366 amino acids showing 98% identity to *S*. *cerevisiae* Hog1 and the stress-activating kinase SakA of *A*. *nidulans*. The results of multisequence alignment revealed that Hog1 was very conserved across *Aspergillus* species (Supplementary Figure S1A). The predicted SI65_07698 protein was found to contain a Pkc-like superfamily domain (Supplementary Figure S1C), which was consistent with the conserved domain of *hogA* in *A*. *nidulans*. SI65_07698 was named *Achog1* based on these analysises. It is worth noting that halophilic *Aspergillus* contains 3 Hog1 homologs, which are differentially regulated during different salinity conditions (Rodríguez-Pupo et al., 2021). *A*. *cristatus* does not contain a second Hog1 homolog, as it occurs in most aspergilli (Kawasaki et al., 2002; Garrido-Bazán et al., 2018). *Achog1* deletion mutants were obtained by gene replacement with the hygromycin B phosphotransferase gene (*hph*) as a selective marker to verify the function of *Achog1* in *A*. *cristatus*. Six Δ*Achog1* transformants were ultimately obtained (Supplementary Figure S2B shows five of the transformants). The expression of *Achog1* in Δ*Achog1* strain was detected and the results showed that *Achog1* was not expressed in Δ*Achog1* strain, which further indicated that *Achog1* had been successfully knocked out (Supplementary Figure S2D, the last column). Real-time fluorescent PCR showed that the numbers of *hph* in Δ*Achog1* were single copies (Supplementary Figure S3 and Supplementary Table S2). Eighteen Δ*Achog1-C* strains with the WT-like phenotype were verified by PCR. The results are shown in Supplementary Figure S2C.

### *Achog1* promoted conidial germination and increased conidial number under hypertonic stress

**Figure 1:**
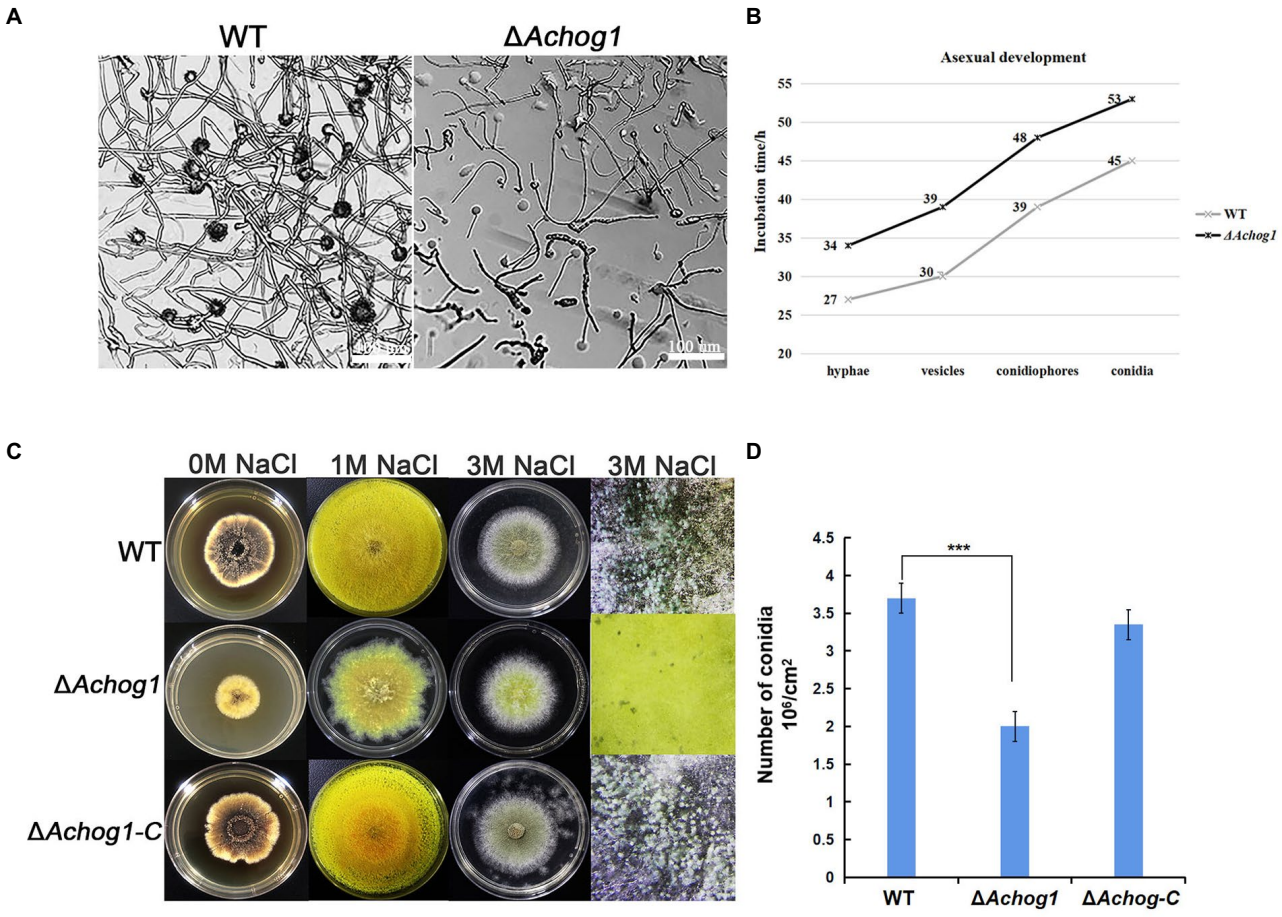
The effect of *Achog1* on asexual sporulation. **(A)** The morphology of WT and Δ*Achog1*. All strains were cultivated on MYA medium containing 3 M NaCl at 37°C for 39 h. **(B)** Incubation time of asexual development. **(C)** The morphology of WT and Δ*Achog1*. All strains were cultivated on MYA medium containing 1 M and 3 M NaCl at 37°C for 7 days. **(D)** Conidia of WT and Δ*Achog1* under stereoscopic microscopy. Statistics of the number of conidia produced by the WT, Δ*Achog1* and Δ*Achog1-C* strains cultivated on MYA medium containing 3 M NaCl at 37°C for 7 days. The total number of conidia per fixed area was counted with a haematocytometer under a microscope, and the data were used to calculate the number of conidia per cm^2^. Error bars represent the standard deviation of at least three replicates. Values that differed significantly from the value for the WT strain according to a *t test* are indicated with asterisks (***p < 0.001).

**Figure 2:**
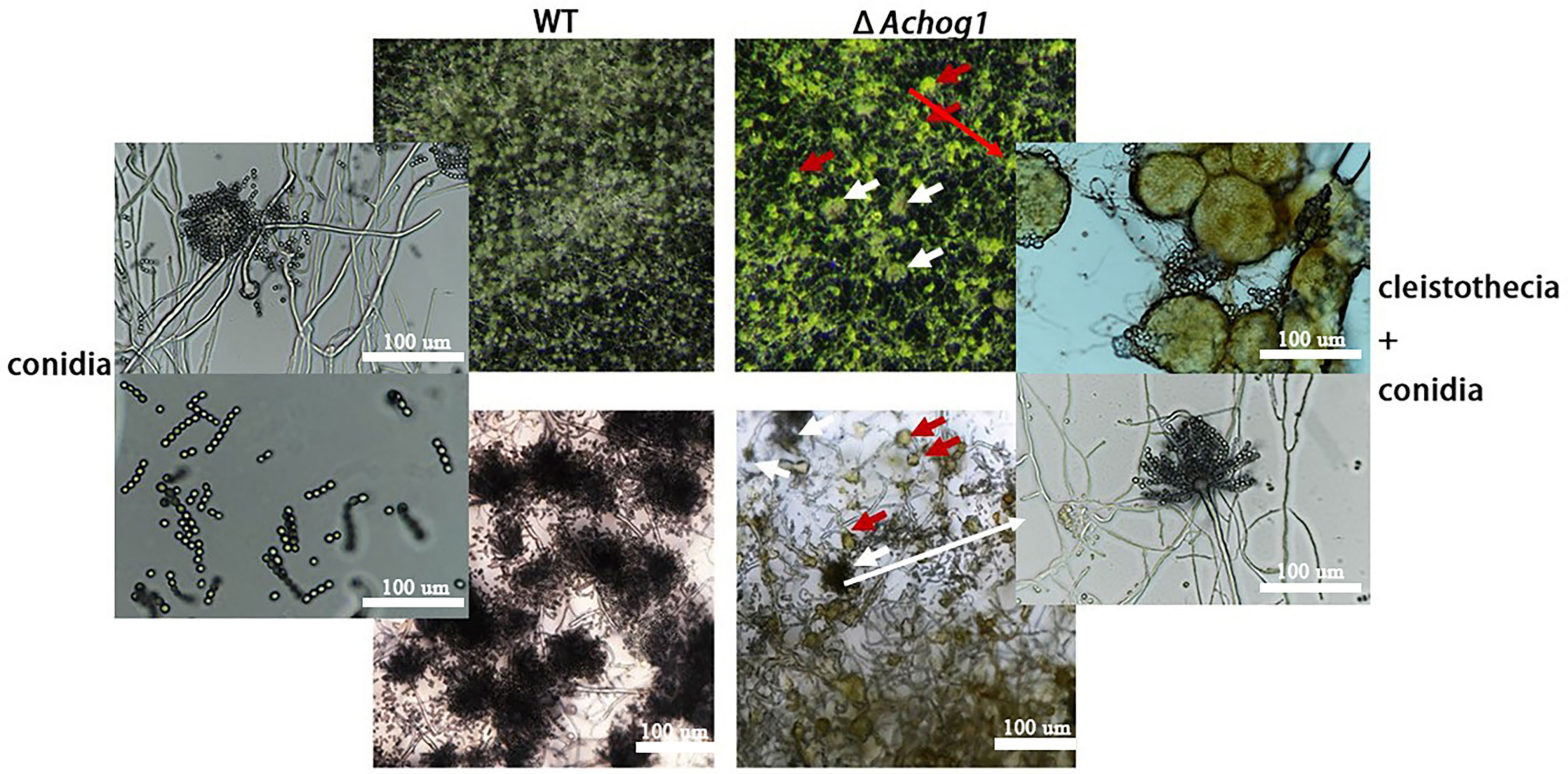
Microscopic observation of WT and Δ*Achog1*. WT and Δ*Achog1* strains cultivated on MYA medium containing 3 M NaCl at 37°C for 14 days. The red arrows refer to cleistothecia, and the white arrows refer to conidia.

*Aspergillus cristatus* could produce pure sexual/asexual spores under hypotonic and hypertonic stress. It was speculated that there was an association between osmotic stress and sporulation. Therefore, the sexual and asexual development of WT and Δ*Achog1* was observed under different osmotic stresses by inoculating an equal amount of conidia (concentration: 1 × 10^6^ conidia/mL). When hyphae of the WT strain formed conidiophores, most hyphae of Δ*Achog1* only formed vesicles at 39 h on MYA media containing 3 M NaCl (Figures 1A,B, indicating that deleting *Achog1* delays the germination of conidia. A large number of grey-green conidia were produced by WT, while only a small number of conidia was produced by Δ*Achog1* in the center of the colony as the incubation time increased (Figure 1C). The conidial number produced by WT was nearly 2 times that produced by the Δ*Achog1* strain (Figure 1D). No significant difference was found in sexual development between WT and Δ*Achog1* on hypotonic media (Figure 1C: the first column). However, hyphal curling appeared in Δ*Achog1* after culture with 3 M NaCl for 7 days, and a small amount of golden yellow cleistothecia appeared in the Δ*Achog1* strain after culturing for 14 days (Figure 2), which was an interesting phenomenon. These results indicated that *Achog1* positively regulated asexual sprouting and might inhibit sexual development under hypertonic stress in *A*. *cristatus*.

### Role of *Achog1* in the response to hypertonic stress

**Figure 3:**
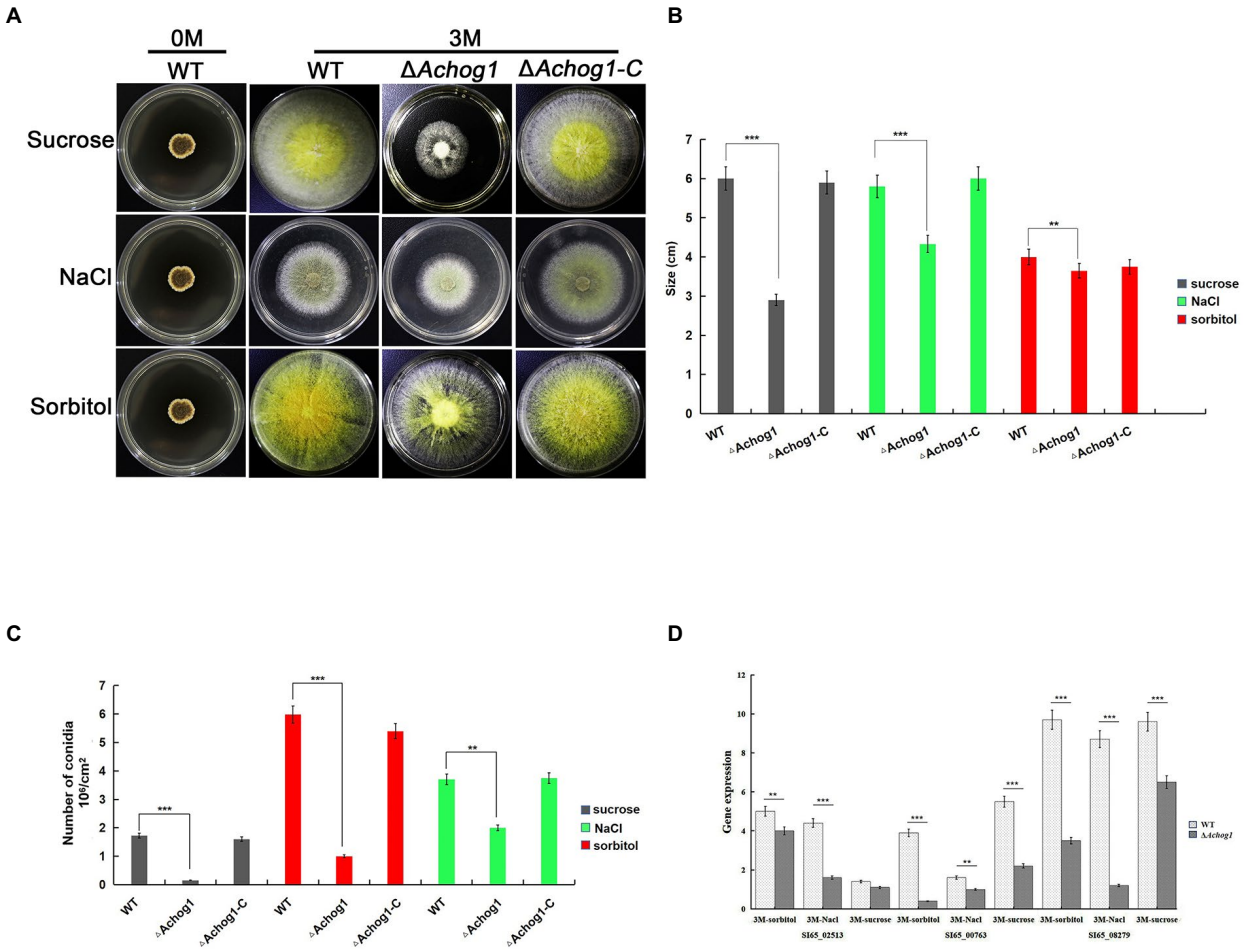
Effect of *Achog1* on the osmotic stress response. **(A)** Colony morphology of WT, Δ*Achog1* and Δ*Achog1-C* strains grown in medium with 1 M，3 M sucrose，sorbitol and NaCl at 37°C for 7 days; **(B)** Statistics of the number of conidia produced by WT, Δ*Achog1* and Δ*Achog1-C* strains grown in medium with 1 M，3 M sucrose, sorbitol and NaCl at 37°C for 7 days; **(C)** Statistics of the colony diameters in medium with 1 M, 3 M sucrose, sorbitol and NaCl at 37°C for 7 days; **(D)** Expression of genes related to the response of high osmotic stress. Error bars indicate the standard deviation of at least three replicates. Values that differed significantly from the value for the WT strain according to a *t test* are indicated with asterisks (**p < 0.01, ***p < 0.001).

It is well known that one of the main functions of *hog1* is to respond to hyperosmolarity in other fungi. The conidia of WT and Δ*Achog1* (concentration: 1 × 10^6^ conidia/mL) were cultivated on MYA media with 3 M sorbitol and sucrose to confirm whether *Achog1* is involved in hypertonic stress. The growth of Δ*Achog1* was significantly slower than that of WT on MAY media with 3 M sucrose, and Δ*Achog1* also grew slowly on media with 3 M NaCl and sorbitol (Figures 3A,B). Furthermore, the expression of genes related to hypertonic stress was detected. The results showed that the expression of genes related to hypertonic stress in Δ*Achog1* was lower than that of in WT under hypertonic stress (Figure 3D). Fewer conidia were produced by Δ*Achog1* (Figure 3C). The results of sensitivity tests showed that *Achog1* could positively regulate the response to hypertonic stress, and the sensitivity of Δ*Achog1* to different osmotic stress regulators was in descending order: 3 M sucrose >3 M NaCl >3 M sorbitol.

### *Achog1* positively regulates oxidative stress tolerance

**Figure 4:**
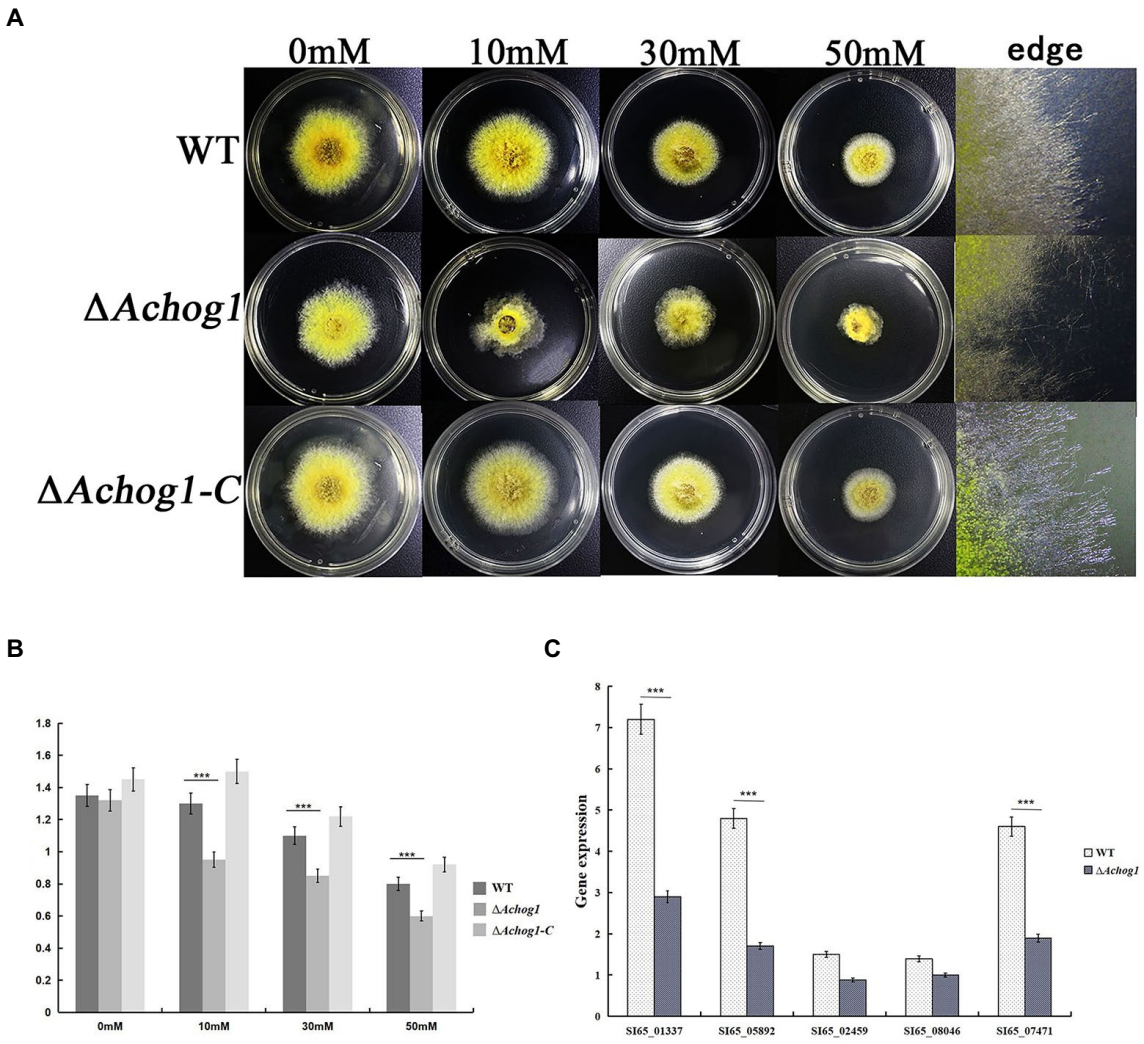
Effect of *Achog1* on the oxidative stress response. **(A)** Colony morphology of WT and Δ*Achog1* under MYA medium with different concentrations of H_2_O_2_ at 28°C for 3 days. **(B)** Colony diameters of WT and Δ*Achog1*. **(C)** Expression of genes related to the response of oxidative stress. Error bars indicate the standard deviation of at least three replicates. Values that differed significantly from the value for the WT strain according to a *t test* are indicated with asterisks (***p < 0.001).

A study showed that *hog1* mediated the oxidative stress pathway in *C*. *albicans* (Thomas et al., 2013). The conidia of WT and Δ*Achog1* (concentration: 1 × 10^6^/mL) were cultivated in MYA with different concentrations of H_2_O_2_ (0 mM, 10 mM, 30 mM, and 50 mM) to verify whether *Achog1* was involved in oxidative stress. The colony diameter of Δ*Achog1* decreased, and its edges were irregular (Figures 4A,B), suggesting that the tolerance of Δ*Achog1* to oxidative stress was significantly reduced. We detected the expression of genes related to oxidative stress. The results showed that the expression of genes related to oxidative stress in Δ*Achog1* was lower than that of in WT under oxidative stress (Figure 4C). When the *Achog1* gene was complemented, the sensitivity to H_2_O_2_ was essentially restored. These results indicated that *Achog1* positively regulated the response to oxidative stress.

### *Achog1* positively regulates the response to alkali stress

**Figure 5:**
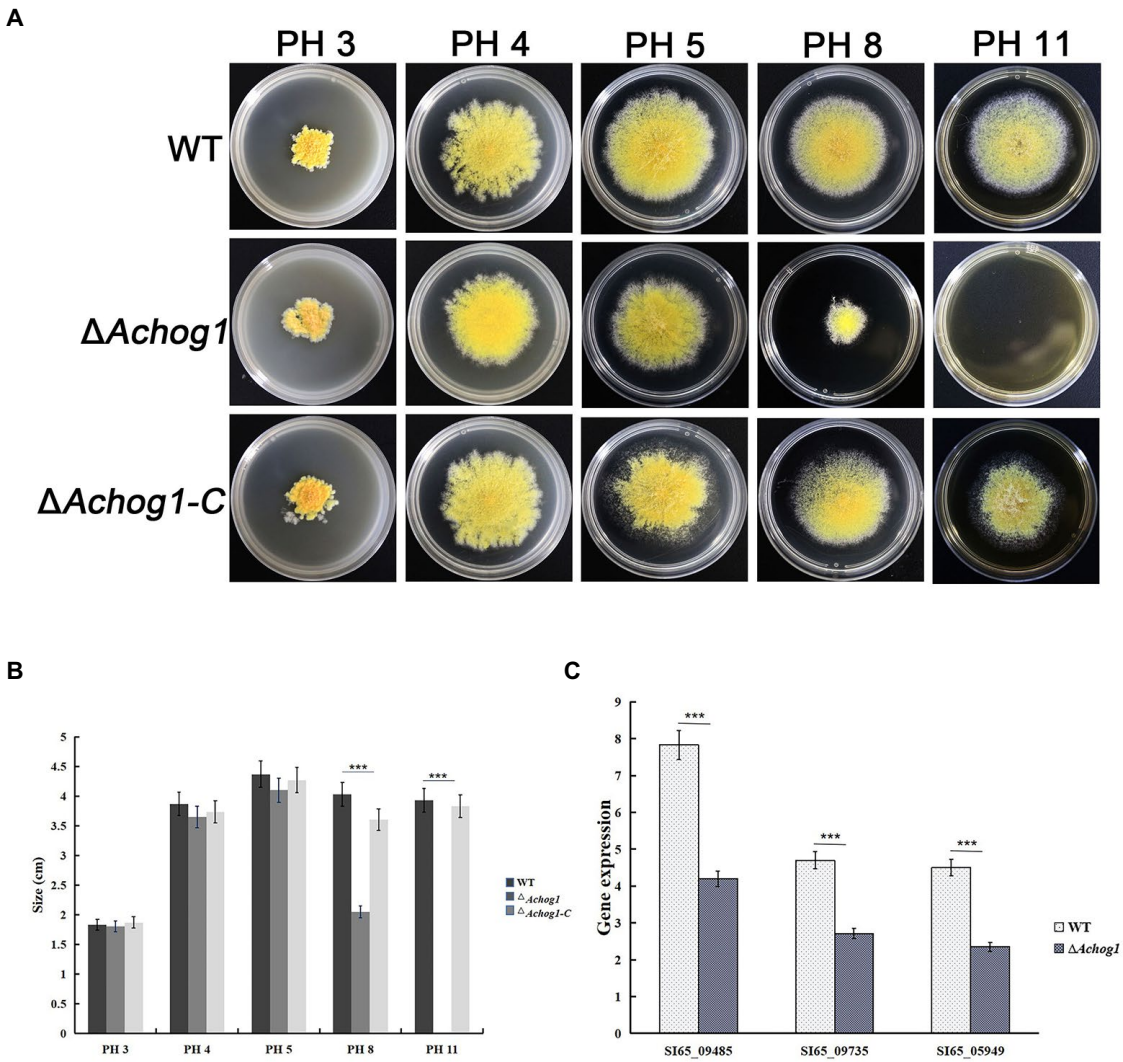
Effect of *AChog1* on the pH stress response. **(A)** Colony morphology of the WT, Δ*Achog1* and Δ*Achog1*-C strains on MYA containing 5% NaCl with different pH values at 28°C for 6 days. **(B)** Colony diameter. **(C)** Expression of genes related to the response of pH stress. Error bars indicate the standard deviation of at least three replicates. Values that differed significantly from the value for the WT strain according to a *t test* are indicated with asterisks (***p < 0.001).

It was shown that the Δ*Afhog1* deletion mutant strain grew slower than the wild type strain in medium containing high PH in *A*. *fumigatus* (Ma et al., 2012). To test whether *Achog1* was involved in PH stress, equal amounts of conidia (1.0 × 10^6^ conidia/mL) of WT, Δ*Achog1*, and Δ*Achog1-C* were inoculated on MYA media containing 5% NaCl with different pH values (pH 3, pH 4, pH 5, pH 8, and pH 11). Δ*Achog1* grew normally in media at pH 3–5, its growth was just slower than that of the WT and Δ*Achog1-C* strains after culturing for 6 days at 28°C. However, the growth of Δ*Achog1* significantly slowed down in the media at pH 8, and Δ*Achog1* could not grow at pH 11 (Figures 5A,B). Hyphae samples cultured in the medium with pH 8 were collected to detect the expression of genes related to pH stress. The results showed that the expression of genes related to pH stress in Δ*Achog1* was lower than that of in WT (Figure 5C); indicating that *Achog1* is mainly involved in alkali stress.

### Deletion of *Achog1* reduces pigment formation

**Figure 6:**
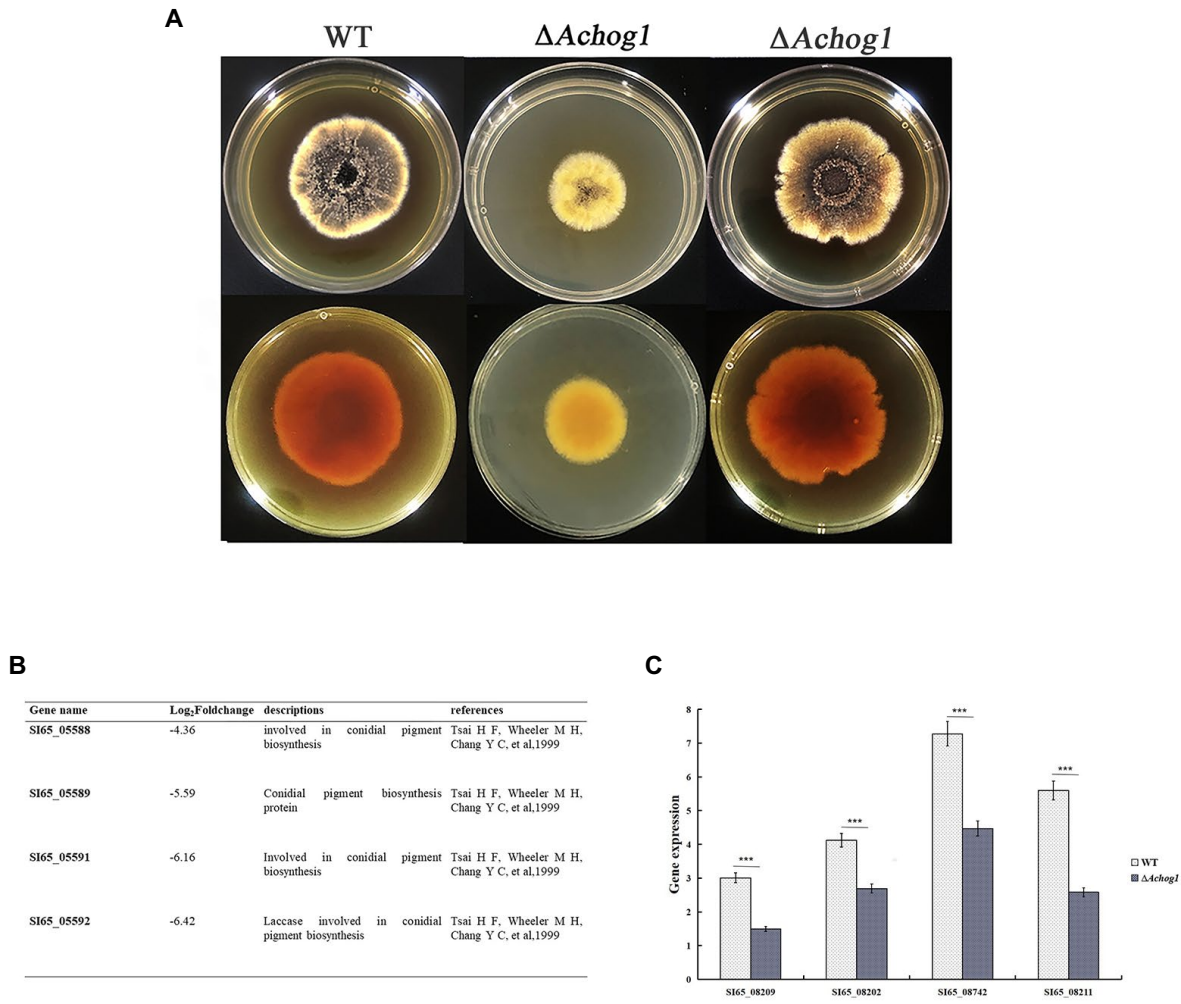
Pigmentation of the WT, Δ*Achog1*, Δ*Achog1-C* strains. **(A)** Pigmentation of the WT, Δ*Achog1*, and Δ*Achog1-C* strains on MYA medium at 28°C for 7 days. **(B)** Genes related to pigmentation according to the results of RNA-seq. **(C)** Expression of genes related to pigment synthesis.

The colonies of the WT strain were brown-black in the center and yellow at the edge on MYA media. However, the colonies of the Δ*Achog1* strain were brown, and their edges were pale yellow. The pigments produced by the Δ*Achog1-C* strains were similar to those produced by the WT (Figure 6A). Genes involved in pigment synthesis, such as SI65_05588 (*ayg1*), SI65_05589 (*arp2)*, SI65_05591 (*abr1*) and SI65_05592 (*abr2*), were downregulated according to RNA-seq data (Figure 6B). Furthermore, we detected the expression of four genes related to pigment synthesis. The results showed that the expression of these genes in Δ*Achog1* strain was lower than that of in WT (Figure 6C). These results suggested that the *Achog1* gene played a role in regulating pigment synthesis in *A*. *cristatus*.

### Analysis of proteins interacting with *AcHog1*

**Figure 7:**
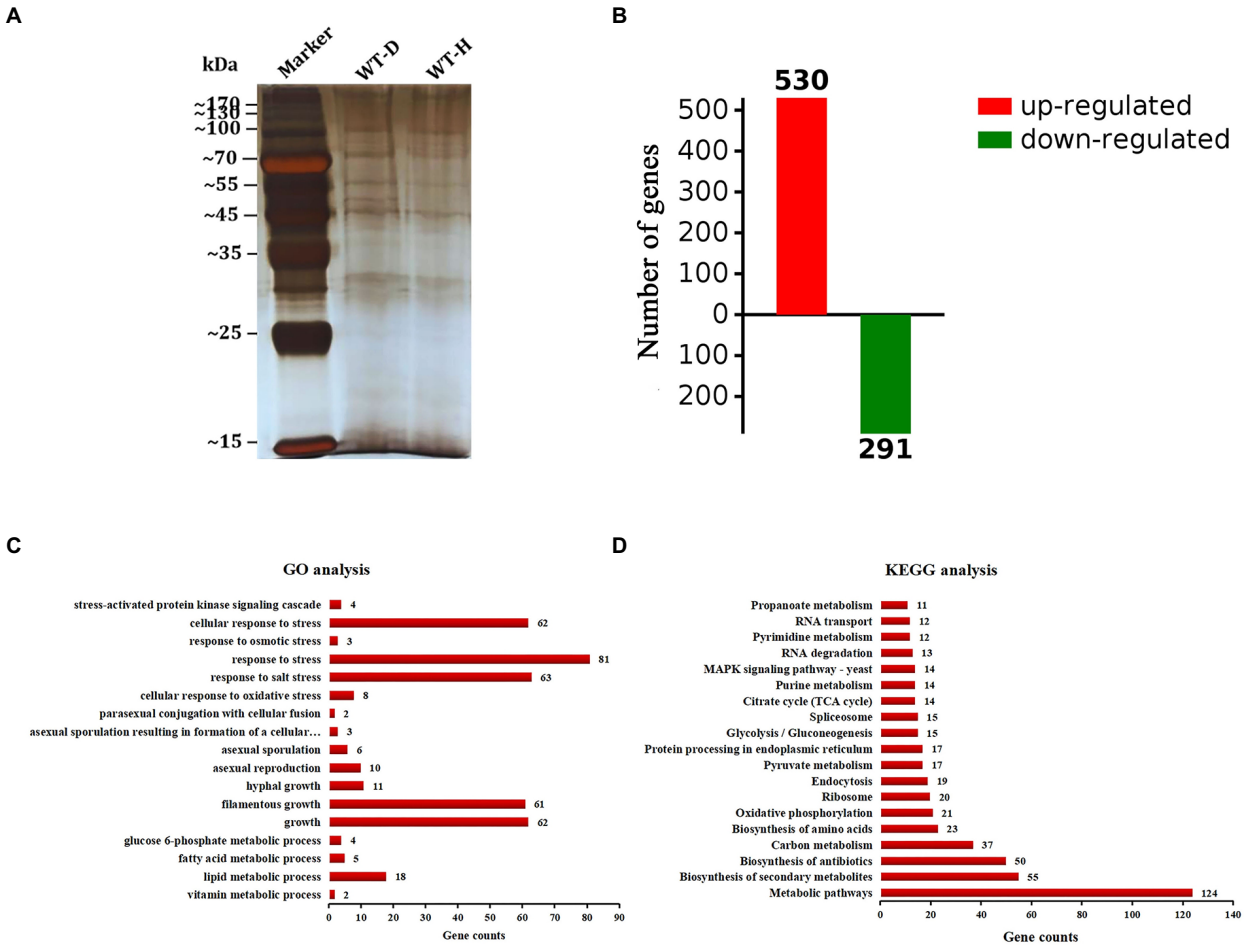
Analysis of differentially expressed proteins. **(A)** Proteins were pulled down in WT-D and WT-H. **(B)** Differential proteins (FC ≥ 2 were considered up-regulatregulated, and FC ≤ 0.5 proteins were considered down-regulreglated). **(C)** GO enrichment analysis. **(D)** KEGG enrichment analysis. The *X*-axis shows the number of proteins. The *Y*-axis shows the pathway names.

A pull-down mass spectrometry experiment was performed to determine the proteins in WT-D and WT-H (Figure 7A). The total ion chromatogram and evaluation chart of the identified proteins showed that the mass spectrometry data were normal, indicating that the identified proteins were accurate (Supplementary Figure S4). A total of 940 proteins and 2,980 peptides were identified (Supplementary Tables S8, S9). A total of 530 proteins were upregulated, and 291 proteins were downregulated (proteins with a fold change (FC) ≥ 2 were upregulated, and those with an FC ≤ 1/2 were downregulated; Figure 7B). Comparing WT-H with WT-D, 483 were present only in WT-H, and 251 were present only in WT-D, demonstrating that there were significant differences in the proteins interacting with AcHog1 between the normal stress and hypertonic stress conditions.

The top 20 proteins with the highest scores are listed in Supplementary Table S3. Among them, 5 proteins are uncharacterized, and the remaining 15 proteins are known functions. For example, the protein A0A1E3B957 is 6-phosphogluconate dehydrogenase. It has been reported that 6-phosphogluconate dehydrogenase is mainly involved in the salt stress response in plants (Nemoto and Sasakuma, 2000; Huang et al., 2003). AcHog1 interacted with 6-phosphogluconate dehydrogenase in samples incubated in MYA containing 3 M NaCl, suggesting that AcHog1 could regulate 6-phosphogluconate dehydrogenase to participate in the salt stress response in *A*. *cristatus*. RodA/RodB-related conidia (Pedersen et al., 2011), Ste20 and Cla4 located upstream of the HOG1 pathway were also found in WT-H (Dan et al., 2001; Joshua and Höfken, 2019). The data proved AcHog1 could interact with 6-phosphogluconate dehydrogenase and might be involved in the salt stress response. In addition, *Achog1* could interact with RodA/RodB, Ste20 and Cla4 and *Achog1* might be also involved in the production of conidia and the response of HOG pathway.

**Figure 8:**
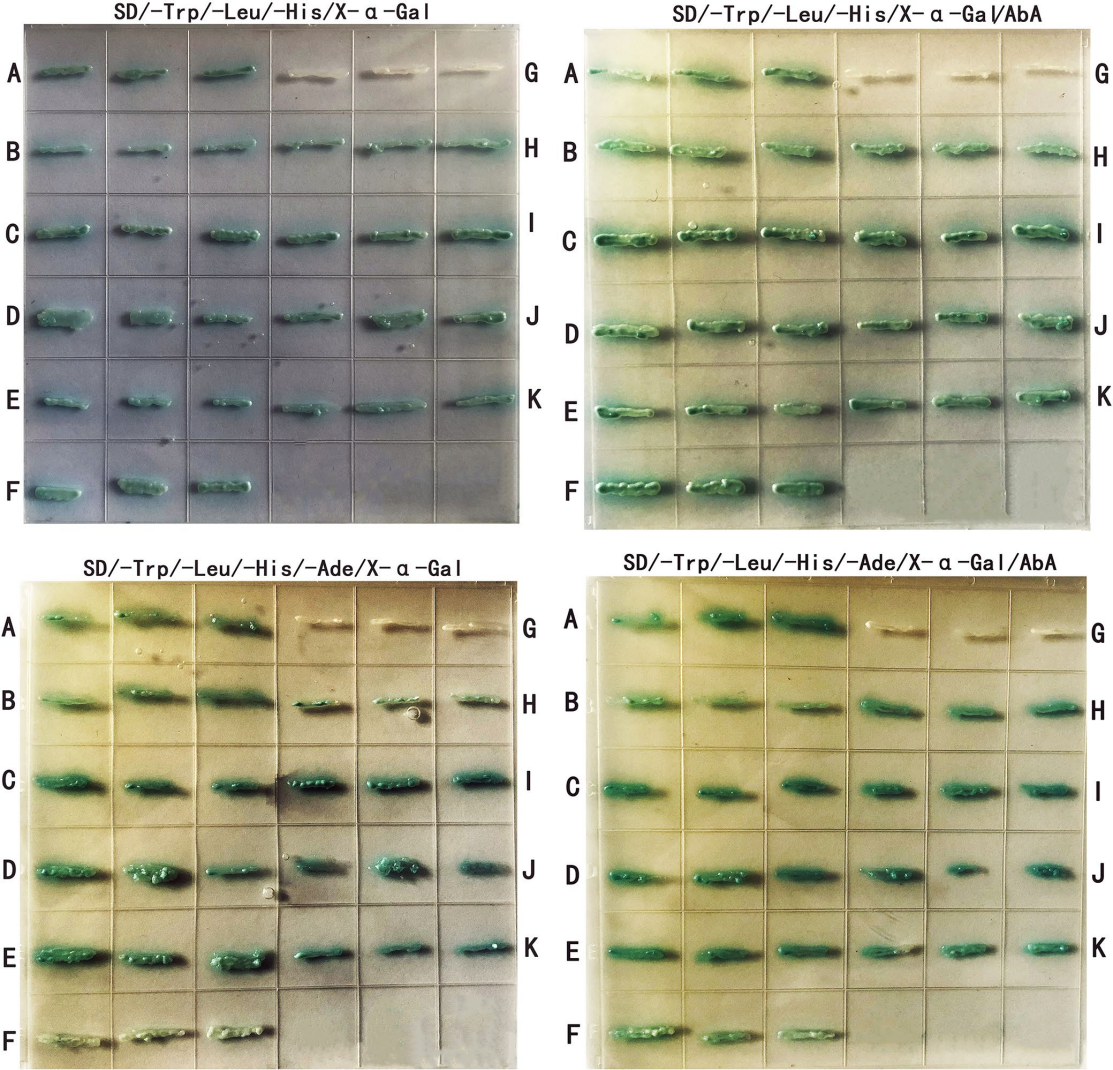
Differential protein yeast two-hybrid validation. **(A–K)** correspond to positive control (PGBKT7-53 + PGADT7-T), SI65_07477 (AcRodB), SI65_06242 (AcSte7), SI65_05510 (AcBmh1), SI65_09934 (AcCsp), SI65-06799 (AcPhnA), negative control (PGBKT7-lam + PGADT7-T), SI65_10255 (AcRodA), SI65_03085 (AcCla4), SI65_07597 (AcSte20), SI65_05898 (AcSko1), respectively.

GO enrichment analysis showed that the differentially expressed proteins were mainly involved in various metabolic processes, asexual development processes and stress responses (Figure 7C). Nine proteins (Supplementary Table S4) related to asexual sporulation and stress responses were randomly selected for yeast two-hybrid validation. PGBKT7-AcHog1 and PGADT7 plasmids containing 9 proteins were co-transformed into yeast AH109 receptive cells. After 5 days of culturing in SD/−Leu/−Trp, a single colony was cultured on SD/−Trp/−Leu/-His and SD/−Trp/−Leu/-His/−Ade medium containing X-α-Gal and AbA for 5 days. PGBKT7-53 + PGADT7-T was chosen as a positive control (Figure 8A), and PGBKT7-lam + PGADT7-T was a negative control (Figure 8G). Both the colonies in the positive control and in the experimental groups (Figures 8B–F,H–K) were blue, indicating that all the selected proteins could interact with AcHog1 (Figure 8). These results showed that AcHog1 might regulate asexual sporulation and the response to osmotic stress and oxidative stress in *A*. *cristatus*. KEGG enrichment analysis showed that 14 differentially expressed proteins were enriched in the MAPK pathway and were significantly upregulated (Figure 7D). The AA1E3BER4 (AcSko1) protein is located downstream of AcHog1 in the HOG pathway in WT-H, and its interaction with AcHog1 was verified by using yeast two-hybrid assays (Figures 8B,K). Therefore, we speculated that Sko1 was the target protein of AcHog1, which will be a focus of our future studies.

### Summary of RNA-seq data and RT-qPCR detection of selected genes

To investigate the roles of *Achog1* in *A*. *cristatu*s, RNA-seq of Δ*Achog1* and WT was performed at selected time points. Conidiophores and chain-like conidia appeared on the WT strain; however, only some vesicles were formed in Δ*Achog1*, when the Δ*Achog1* and WT strains were cultivated on MYA media containing 3 M NaCl for 39 h (Figure 1A). Therefore, mycelial samples of the cultures at 39 h were collected for RNA-seq. All sequences obtained by sequencing were uploaded to the NCBI database: Sequence Read Archive,^2^ and get the accession number: PRJNA877470. A summary of the RNA-seq data is reported in Supplementary Table S5. In this study, a total of 1,074 differentially expressed genes (Supplementary Table S10) were obtained from the *Achog1* transcriptome data (|log2(FC)| > = 1 and padj < = 0.05). Among these differentially expressed genes, 480 were upregulated, and 594 were downregulated. The top 20 genes upregulated and downregulated in Δ*Achog1* are listed in Supplementary Tables S6, S7, respectively. Among them, SI65_00200 (*phiA*) is a cell wall protein (Schachtschabel et al., 2012); SI65_01919 encodes a low-affinity glucose transporter, and a study showed that low-affinity glucose required the coordinated activities of the HOG and glucose signaling pathways in *S*. *cerevisiae* (Tomás-Cobos et al., 2004). The protein kinases Dsk1 (SI65_03917) contributed to cell cycle and pre-mRNA splicing (Tang et al., 2012). In addition, genes responding to the HOG pathway and related to sporulation were screened from the RNA-seq data by referring to the relevant literature reports and the genome database of *Aspergillus*. The results were shown in Table 1. It was noted that genes related to asexual development were downregulated, such as *brlA* (SI65_02778) and *wetA* (SI65_00383) (Tsai et al., 1999), were involved in asexual sporulation and downregulated in the Δ*Achog1* mutant. We speculated that *Achog1* could affect the conidia production of *A*. *cristatus* by down-regulating the expression of these genes.

**Table 1:** Expression level detection of sporulation genes in Δ*Achog1* strain.

Gene ID	Gene name	Log_2_FC (Δ*Achog1*/WT)	Function
Genes related to sporalution			
SI65_10255	*rodA*	−5.57	Formation rodlet layer during conidiophore development
SI65_05592	*abr2*	−6.41	Laccase involved in conidial pigment biosynthesis
SI65_02778	*brlA*	−4.08	Regulation of stalk development
SI65_05589	*ayg1*	−5.59	Conidial pigment biosynthesis protein
SI65_05588	*arp2*	−4.36	Involved in conidial pigment biosynthesis
SI65_08417	*dewA*	−4.18	Coating of conidia
SI65_03945	*alb1*	−2.84	Polyketide synthase involved in biosynthesis of the conidial pigment
SI65_05591	*abr1*	−6.15	Involved in conidial pigment biosynthesis
SI65_07477	*rodB*	−1.61	Formation rodlet layer during conidiophore development
SI65_02949	*ppoC*	−1.57	Balance sexual and asexual development
SI65_05213	*silG*	−2.23	Light response regulator
SI65_00414	*treB*	−1.14	Degradation intracellular trehalose during germination
SI65_00383	*wetA*	−1.02	Regulation conidiophore maturation and formation
SI65_10210	*mcmA*	1.05	MADS-box family transcription factor
SI65_04931	*mutA*	1.06	Highly expressed during sexual development, specifically expressed in Hulle cells
SI65_05022	*ppoB*	1.13	Balance sexual and asexual development
Genes related to HOG pathway			
SI65_09147	*ssk1*	−1.27	Regulated an osmosensing MAPK kinase cascades
SI65_00763	*ypd1*	−6.78	Two-component histidine phosphotransfer protein
SI65_02778	*msn4*	−4.09	Controlled the H_2_O_2_ response

**Figure 9:**
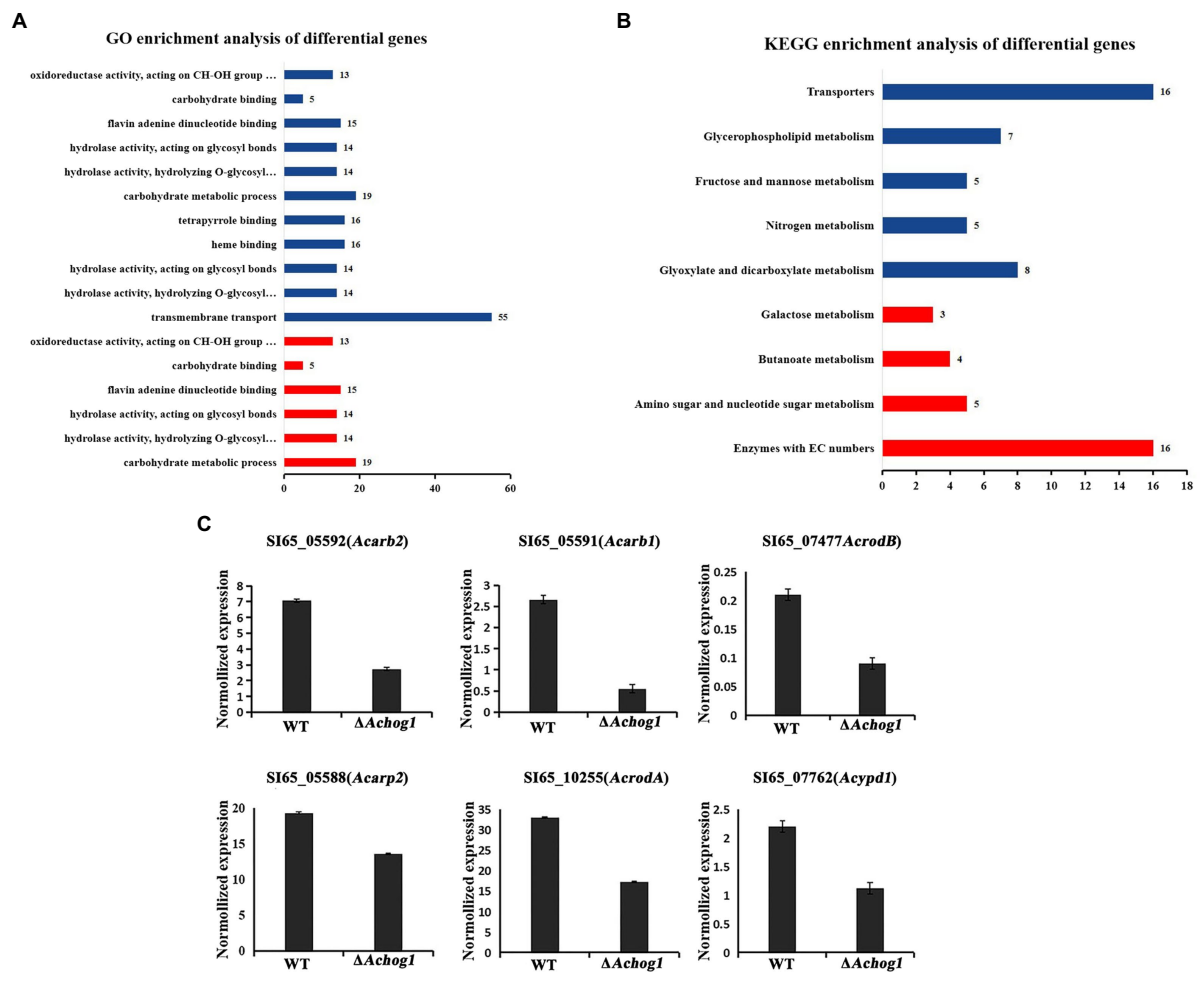
Transcriptional analysis of *ΔAchog1* and WT strains. **(A)** GO analysis of differentially significant enrichment. **(B)** KEGG analysis of differentially significant enrichment. The *X*-axis shows the number of differentially expressed genes. The *Y*-axis shows the pathway names. Red indicates significant upregulation; blue indicates significant downregulation. **(C)** RT-qPCR verification of selected genes.

GO enrichment analysis showed that the differentially expressed genes were mainly enriched in carbohydrate metabolism, transmembrane transport, hydrolase activity, binding and other processes. Carbohydrate metabolic process (GO: 0005975), hydrolase activity (GO: 0016798, GO: 0016798) and oxidoreductase activity (GO: 0016614) were significantly upregulated. Transmembrane transport (GO: 0055085) was significantly downregulated (Figure 9A). The differentially expressed genes were mainly enriched in various metabolic pathways and MAPK pathways by KEGG enrichment analysis. Galactose metabolism (ani00052) and amino sugar and nucleotide sugar metabolism (ani00520) were significantly upregulated, glycerophospholipid metabolism (ani00564) and glyoxylate and dicarboxylate metabolism (ani00564) were significantly downregulated (Figure 9B). In addition, all the genes enriched in the MAPK pathway were downregulated. We speculated that *Achog1* also played an important role in hydrolase activity, various metabolic processes and MAPK pathways in *A*. *cristatus*.

To verify the reliability of the expression changes in the transcriptome, 6 genes were selected for RT-qPCR detection (Figure 9C). The gene expression patterns of the gene analysis were similar in the RT-qPCR and RNA-seq, indicating that the transcriptome sequencing results obtained in this study were reliable.

## Discussion

**Figure 10:**
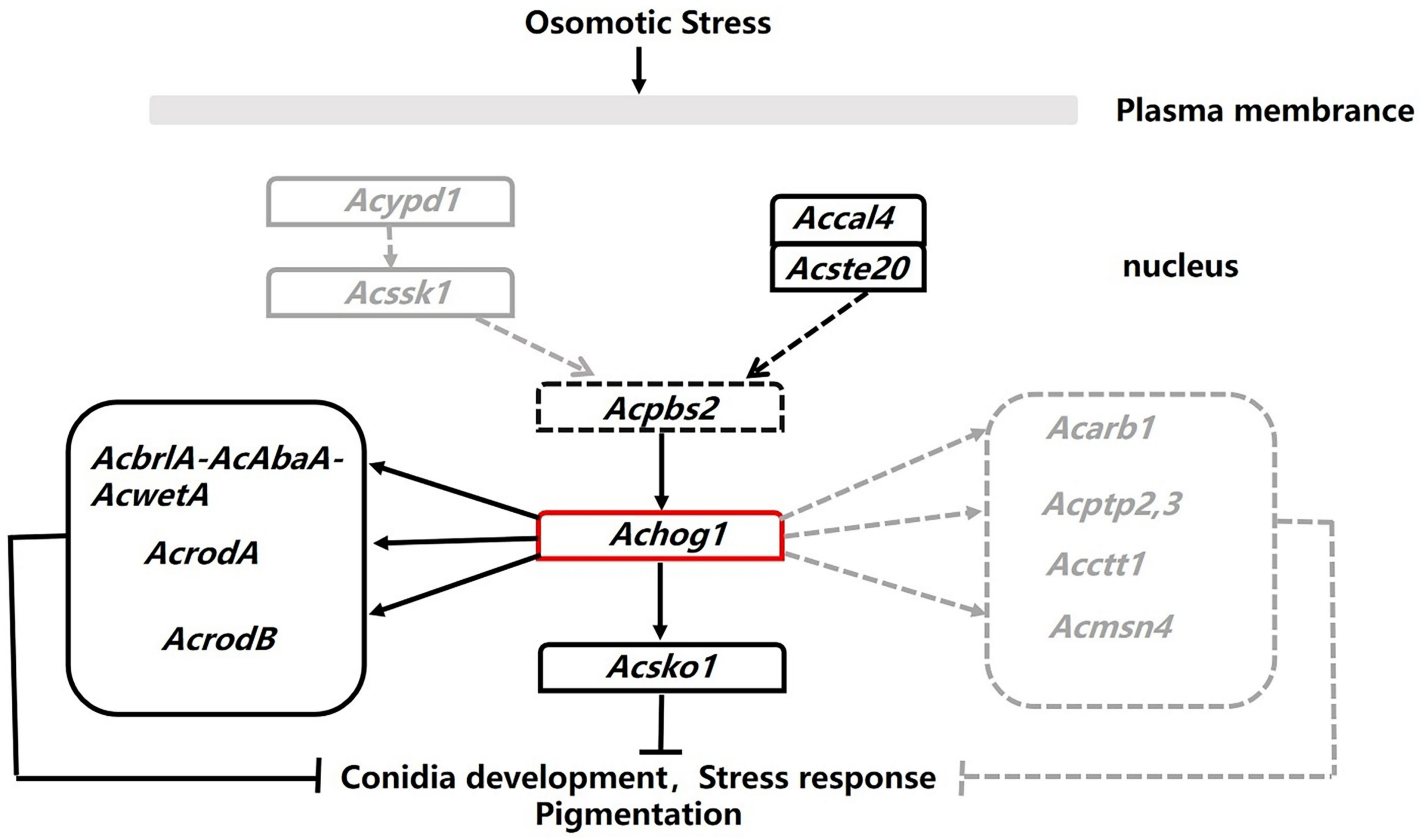
Regulation map of *Achog1*. The black solid lines represented genes from pull-down assays. The gray dotted lines represented genes from the RNA-seq.

*Aspergillus cristatus* is a homothallic filamentous fungus that produces sexual or asexual spores under different osmotic conditions. However, the mechanism that controls reproduction in spores is currently unknown. We speculated that osmotic stress was involved in regulating this process based on the fact that *A*. *cristatus* can only produce ascospores in medium containing 1 M NaCl, while it can only produce conidia in medium containing 3 M NaCl. It is well known that the HOG pathway is one of the main pathways in response to osmotic stress (Gustin et al., 1998; Estruch, 2000; Welsh, 2000; Sean et al., 2004). The key gene of the HOG pathway is *hog1*, which belongs to PKc kinase. A PKc-like gene, SI65_07698, was found in the *A*. *cristatus* genome database by sequence alignment. The conserved domain of the gene was consistent with that of *hog1* in *S*. *cerevisiae*, so it was named *Achog1*. In this study, we constructed the Δ*Achog1* and Δ*Achog1-*C strains to further investigate the function of *Achog1*. The results indicate that *Achog1* plays important roles in asexual development, osmotic stress, oxidative stress, PH stress and pigmentation in *A*. *cristatus*. Moreover, we roughly describe the regulatory pathway (Figure 10) of *Achog1* in *A*. *cristatus* combined with Pull-down mass spectrometry and RNA-Seq.

PKc kinase plays an important role in the growth, development and conidial formation of filamentous fungi (Dickman and Yarden, 1999). When *osm1*, a functional homolog of the yeast *hog1*, was deleted, the number of conidia increased exponentially in mutants under hypertonic conditions (Xu, 2000); In *A*. *nidulans*, the *sakA* mutation showed no apparent effect on asexual development (Kawasaki et al., 2002). In contrast to these studies, Δ*Achog1* produced 2 times fewer conidia under hypertonic stress compared with the WT in our study. The deletion of *hog1* homologs affects the germination of spores in *A*. *fumigatus* (Xue et al., 2004; Du et al., 2006). And Δ*sakA* conidiospores lost viability more rapidly than those from the wild-type strain in *A*. *nidulans* (Kawasaki et al., 2002; Garrido-Bazán et al., 2018). Similarly, a large number of conidiophores were produced in WT strains in media with 3 M NaCl at 39 h, while Δ*Achog1* could produce only vesicles, indicating that conidial germination was delayed. In addition, it was found that a total of 16 genes related to sporulation were significant difference in the expression according to the RNA-seq data. Among them, SI65_10255, SI65_02778, SI65_07477 and SI65_00383 were genes involved in the development of phialides and conidiophores, and their expression levels were significantly down-regulated in Δ*Achog1* strains. Four proteins (A0A1E3B4I1, A0A1E3B9P1, A0A1E3BK58 and A0A1E3BGW2) enriched in the glucose 6-phosphate metabolic process (GO: 0051156) were identified in LC–MS/MS differential protein analysis. The glucose 6-phosphate metabolic process is known to be a key branch of the HMP pathway, which is important in conidial formation of *A*. *nidulans* (Broek, 1997). The HMP pathway is also very active during the conidial formation of *Aspergillus niger* (Ruijter and Visser, 1999). Because the samples selected for Pull-down experiment were *A*. *cristatus* cultured for 7 days under hypertonic stress. Under this culture condition, *A*. *crsitatus* could only produce a large number of conidia. We speculated that the HMP pathway existed in *A*. *cristatus* according to the LC/MS–MS data. And *Achog1* might negatively regulate the four proteins to affect the formation of conidia. *hogA* plays an important role in the regulation of sexual development in *A*. *nidulans*. The progression of cleistothecia formation in Δ*hogA* strains was somewhat precocious (Kawasaki et al., 2002). However, the sexual development between WT and Δ*Achog1* was not significant on hypotonic medium. Interestingly, hyphal curling appeared in Δ*Achog1* at 7 days. *ΔAchog1* strains produced cleistothecia on MYA medium containing 3 M NaCl after 14 days. This phenomenon was rarely observed in the Δ*hog1* strains of other fungi. These results suggested that *Achog1* played a positive role in the asexual development of *A*. *cristatus* and that *Achog1* may regulate the transition between sexual and asexual development, but the specific regulatory mechanism still needs to be further studied.

Osmotic stress is an important factor regulating the growth and development of filamentous fungi (Bahn, 2008; Duran et al., 2010). In this study, the *Achog1* deletion mutant grew significantly slower than the wild type in medium containing a high concentration of NaCl (2 M/3 M), indicating that the deletion of *Achog1* reduces the resistance of *A*. *cristatus* to hyperosmolarity. The same results were obtained when we replaced the osmotic pressure regulators with sucrose and sorbitol. We demonstrate that *Achog1* plays a positive role in osmotic stress according to these results. However, the role of *Achog1* in the osmotic response of *A*. *cristatus* contrasts sharply with that of *sakA* in *A*. *nidulans* and *hog1* in other fungi. In *A*. *nidulans*, *sakA* is even dispensable for osmotic stress resistance (Kawasaki et al., 2002; Garrido-Bazán et al., 2018). In other fungi, the resistance of *hog1* to hyperosmolarity is much lower than that in *A*. *cristatus*. For example, 0.4 M NaCl can greatly inhibit colony growth, although it has been shown to be not completely lethal to deletion mutants of *hog1* in *M*. *oryzae* (Montemurro, 1992). After *hog1 is* deleted from *F*. *graminearum*, the mutants cannot grow in media with 0.7 M NaCl (Morawetz et al., 1996). In *Botrytis cinerea*, *hog1* mutant strains cannot grow on media containing 1.5 M NaCl (Morawetz et al., 1996). Although the functions of *hog1*-type MAPKs are relatively conserved, they might play diverse roles in different fungi. In addition, the *Achog1* deletion mutant exhibited a significant decrease in conidiation compared with the WT strain in the presence of 3 M NaCl, indicating that *Achog1* can promote conidia production under hyperosmotic stress.

*hog1* responds not only to osmotic stress but also to oxidative stress (Alonso-Monge et al., 2003). An oxidative stress sensitivity test was performed on *Achog1* deletion mutants. It was found that Δ*Achog1* grew slowly in media containing different concentrations of H_2_O_2,_ especially at high concentrations of H_2_O_2_. Our experiments indicate that *Achog1* plays a positive role in the regulation of the oxidative stress response. This finding was similar to that observed in *A*. *fumigatus* and *F*. *graminearum*. In *A*. *fumigatus*, H_2_O_2_, as a mimic of oxidative stress, significantly inhibited the growth of *hog1* mutant strains (Du et al., 2006). The growth of deletion mutants of each individual gene involved in the *HOG* pathway was suppressed under oxidative stress in *F*. *graminearum* (Montemurro, 1992). Moreover, the hyphae of Δ*Achog1* were extremely irregular at the edge, suggesting that the polar growth of hyphae was affected under oxidative stress. In the hypertonic sample (WT-H), many proteins interacting with AcHog1 were enriched in the stress response pathway. Moreover, some differentially expressed genes related to the stress response were also identified among the Δ*Achog1* transcriptome data. Sixty-five genes were enriched in oxidoreductase activity molecular function, and 30 genes were downregulated. We speculate that *Achog1* can maintain the balance of intracellular and extracellular stress by downregulating the expression of these proteins and genes so that Δ*Achog1* can grow normally under hypertonic stress and oxidative stress when high-concentration stress stimulates *A*. *cristatus*.

*Achog1* is also involved in pH stress. In our study, both acid and alkali stress tests were carried out and found that Δ*Achog1* grew slower in the media at pH 8 than WT. Δ*Achog1* could not even grow in media at pH 11, which was similar to the results of a study in *A*. *fumigatus* (Ma et al., 2012). However, acid stress has not been studied in *A*. *fumigatus*. Studies showed that low pH-induced structural changes were dependent on *hog1* when yeast cells were cultivated in media in which the pH was shifted from 5.5 to 3.5 in yeast (Bilsland et al., 2004; Lawrence et al., 2004; Sotelo and Rodriguez-Gabriel, 2006; Kapteyn et al., 2010). However, our acid stress test showed that Δ*Achog1* could grow normally. These results showed that *Achog1* mainly participates in the response to alkaline stress.

Pigment is a type of secondary metabolite produced by fungi during their growth and development. On MYA media, compared with the WT strain, the *Achog1* deletion mutant produced less pigment. Fifty-seven genes were annotated to secondary metabolite synthesis according to the RNA-seq data. Among these genes, SI65_05588 (*ayg1*), SI65_05589 (*arp2)*, SI65_05591 (*abr1*) and SI65_05592 (*abr2*) were involved in the regulation of pigment synthesis, and they all were downregulated in Δ*Achog1*, indicating that *Achog1* participates in the regulation of secondary metabolism and may be involved in the regulation of pigment synthesis. *hog1* plays an important role in regulating fungicide resistance. *Os-2* is a gene with high homology to hog1 in *N*. *ceassa*. The deletion mutant of *Os-2* was found to be resistant to N-moncootyl dicycloheptene dimethylphthalide and phenylpyrroliac fungicides (Kojima et al., 2004). However, *A*. *cristatus* is a beneficial fungus, we did not performed the fungicide sensitivity test.

The pull-down mass spectrometry and RNA-seq data were consistent with the phenotypic changes observed in the *Achog1* deletion mutant. In this study, pull-down combined mass spectrometry under normal and hypertonic conditions was performed. The proteins interacting with AcHog1 were quite different under normal stress and hypertonic stress conditions. Proteins associated with conidial development could interact with AcHog1 under hypertonic stress. Among them, AcRodB and AcRodA were verified by yeast two-hybrid assays. We speculate that AcHog1 can regulate them to promote conidia production. We also found proteins Ste20 and Cla4 located upstream of Hog1 and protein Sko1 located downstream of Hog1, and yeast two-hybrid assays showed that all three proteins could interact with AcHog1. The interactions between AcHog1 and them are represented by using solid lines in Figure 10. RNA-seq results showed that the expression of genes related to conidial formation, such as SI65_02778 (*AcbrlA*), SI65_07477 (*AcrodB*), SI65_10255 (*AcrodA*) and other genes, was significantly reduced, indicating that *Achog1* could regulate these genes to control the conidial development of *A*. *cristatus*. Consequently, *Achog1* could respond to H_2_O_2_ stress by regulating the gene SI65_02778 (*Acmsn4*), which regulates the H_2_O_2_ stress response (Hasan et al., 2010). *ypd1* and *ssk1* were located upstream of *hog1*, and *ptp2* and ptp3 were located downstream of *hog1*. When the *Achog1* gene was deleted, the expression of *ypd1*, *ssk1*, and *ptp2*,*3* was downregulated. We speculate that *Achog1* can regulate the expression of these genes to affect conidia production and stress responses in *A*. *cristatus*, which requires further study. Therefore, it is represented by gray dotted lines in Figure 10. The results of GO enrichment analysis and KEGG analysis showed that both differentially expressed genes and differentially expressed proteins were significantly enriched in secondary metabolic pathways, and four genes were related to pigment synthesis, indicating that *Achog1* also participates in secondary metabolism.

## Conclusion

In conclusion, this report is the first to present a functional analysis of *Achog1* encoding a Pkc-like superfamily in *A*. *cristatus*. Our data suggest that *Achog1* plays a key role in positively regulating asexual sporulation, stress response, pH response and pigmentation. Pull-down combined mass spectrometry and RNA-seq data provide a foundation for future genetic and biochemical analyses, which will be required for regulatory mechanisms and links between the control of asexual development, stress sensing and secondary metabolism in *A*. *cristatus*.

## Data availability statement

The original contributions presented in the study are included in the article/Supplementary material, further inquiries can be directed to the corresponding authors.

## Author contributions

LS, YT, and ZL conceived and designed the experiments. LS and SS performed the experiments. YW and YL took part in the data analysis. LS drafted the manuscript. YT and ZL revised the manuscript. All authors read, corrected and approved the final manuscript.

## Funding

This study was supported by the National Natural Science Funds of China (Nos. 31960018 and 31660021) and the Subsidy from NSFC of Guizhou Academy of Agricultural Sciences (Nos. 202104 and 202138).

## Conflict of interest

The authors declare that the research was conducted in the absence of any commercial or financial relationships that could be construed as a potential conflict of interest.

## Publisher’s note

All claims expressed in this article are solely those of the authors and do not necessarily represent those of their affiliated organizations, or those of the publisher, the editors and the reviewers. Any product that may be evaluated in this article, or claim that may be made by its manufacturer, is not guaranteed or endorsed by the publisher.

## Footnotes

^1^ http://primer3.ut.ee/

^2^ http://www.ncbi.nlm.nih.gov/sra/
