# Supplementary material for: Achog1 is required for the asexual sporulation, stress responses and pigmentation of Aspergillus cristatus
Source: Front Microbiol. 2022 Nov 25;13:1003244. doi: 10.3389/fmicb.2022.1003244 (PMC9733950; doi:10.3389/fmicb.2022.1003244)
Supplement: Supplementary file 1 [file Data_Sheet_1.doc]

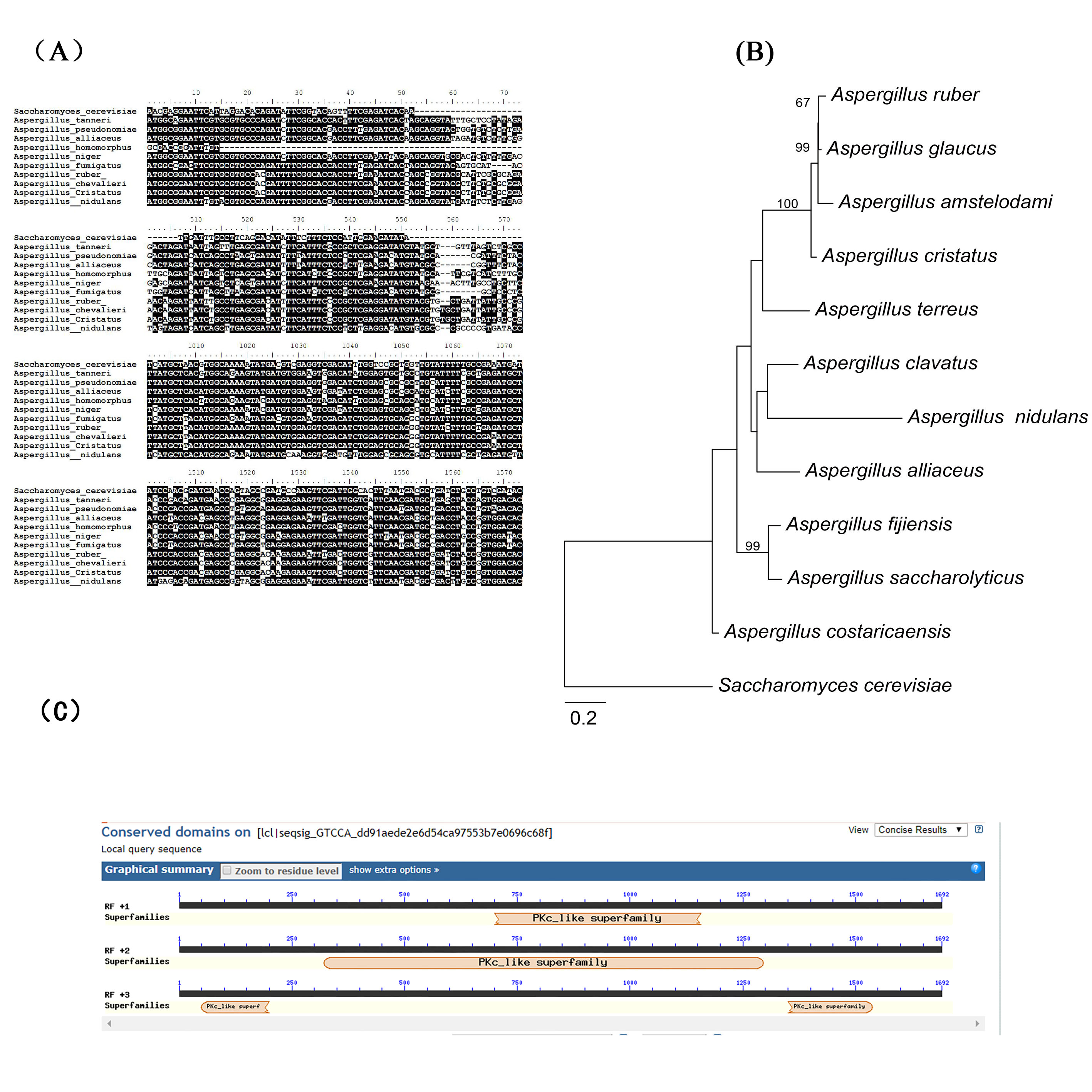


Figure S1. Bioinformatics analysis of the AcHog1*.* (a) Alignment of the putative conserved domains of the amino acid sequence with the sequences. (b) Molecular evolutionary genetics analysis (MEGA) tree showing the phylogenetic relationships of Hog1 fungal homologues. A phylogenetic tree for Hog1 in fungi was created using MEGA6.0. The maximum likelihood method was selected, and the parameters were set at default values. The numbers above the nodes indicated the bootstrap values. (c) The analysis of conserved domain.


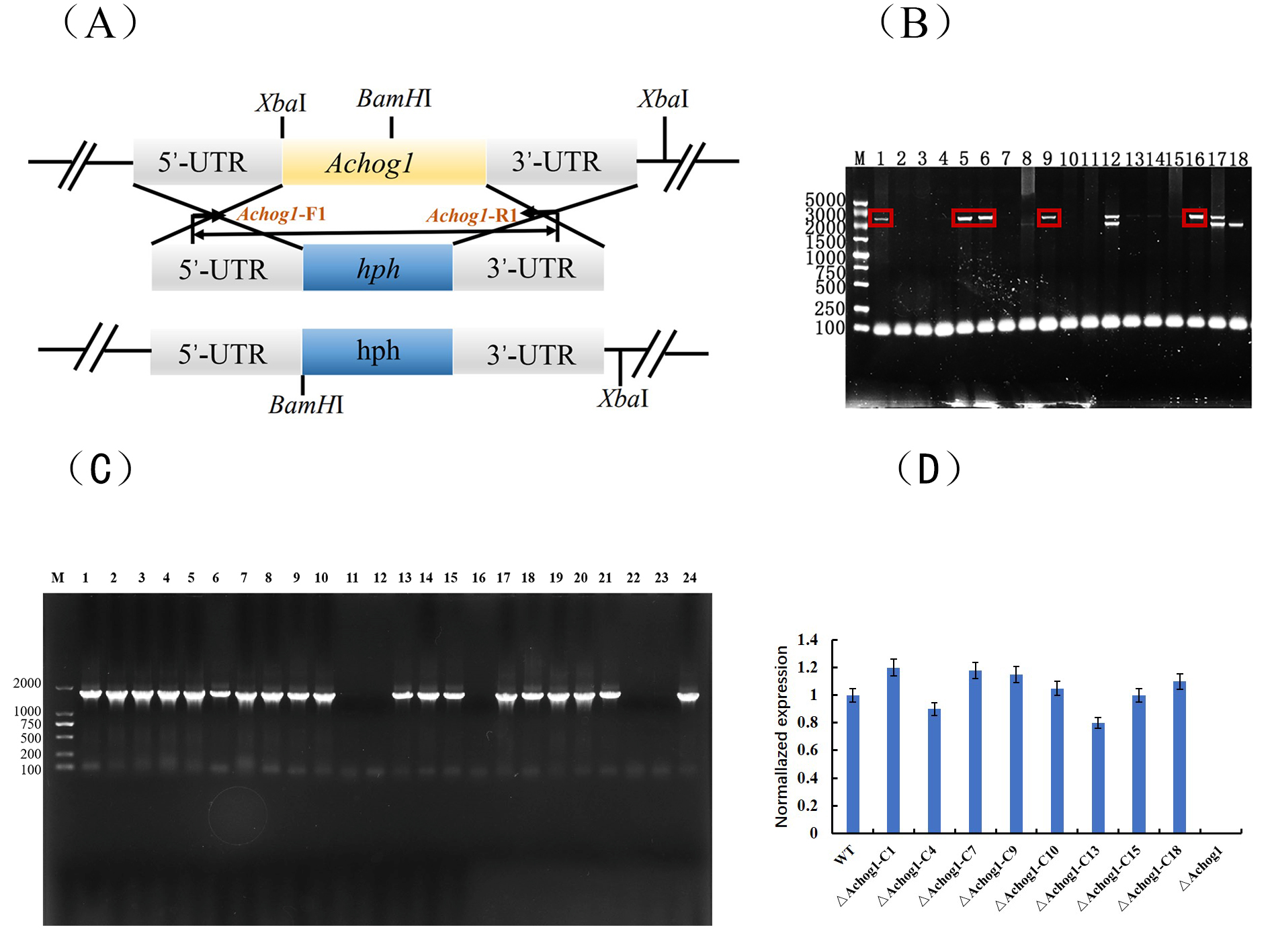


Figure S2. Verification of the Δ*Achog1 and* Δ*Achog1-C* strains (A) Diagram of the gene replacement strategy for the construction of the Δ*Achog1* strain*.* The specific primer used to verify Δ*Achog1* is marked with an arrow. (B) PCR verification of Δ*Achog1.* Lanes 1, 5, 6, 9 and 16 are the amplification results of Δ*Achog1*; lanes 12 and 17 are the amplification results of random insertions; lane 18 is the amplification result of WT. (C) PCR verification of Δ*Achog1-C.* Lane 23 shows the amplification result of Δ*Achog1*; lane 24 shows the wild-type control; (D) RT-qPCR detection of *Achog1* expression levels in Δ*Achog1-C*


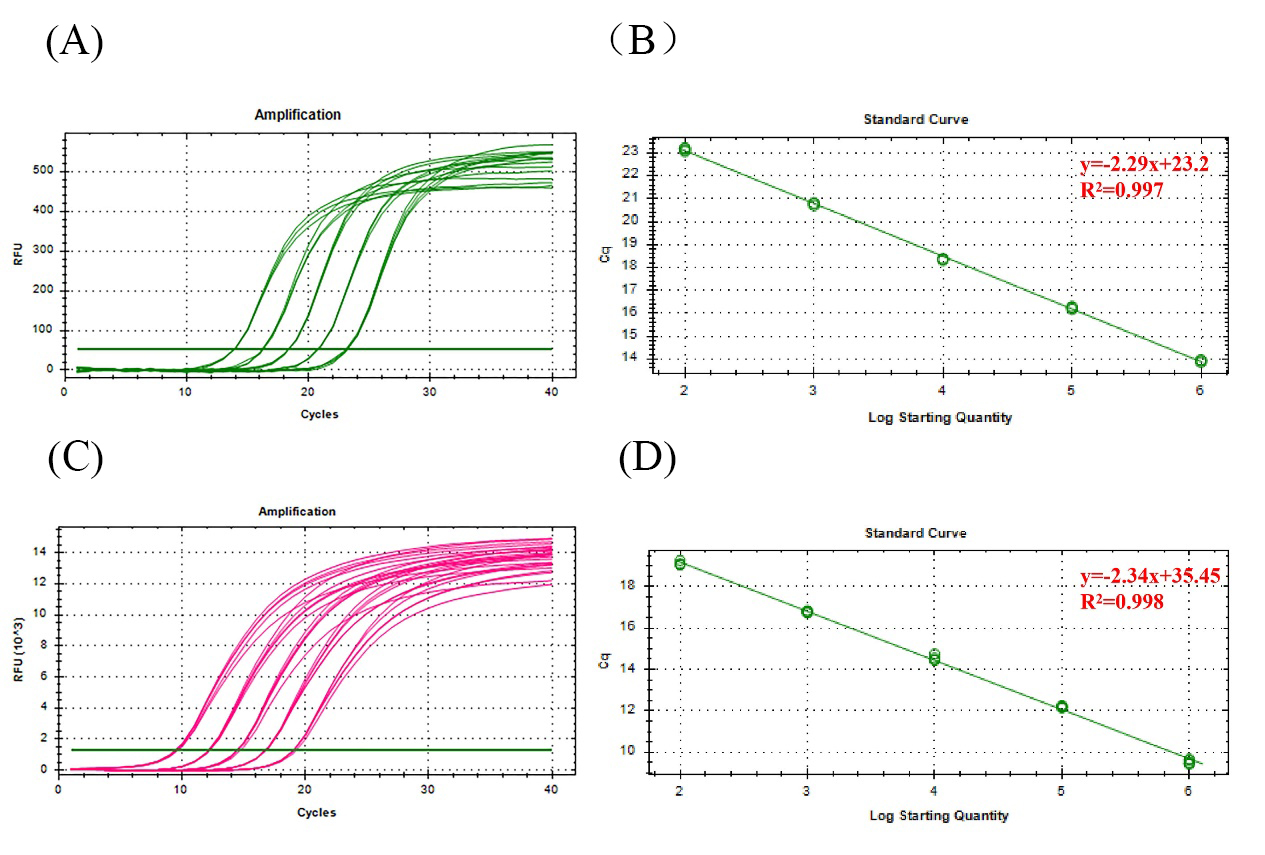


Figure S3. Real-Time PCR detects transgene copy number (A).*GAPDH* gene dynamic curve; (B) *GAPDH* gene standard curve; (C) *hyg* gene dynamic curve; (D) *hyg* gene standard curve.


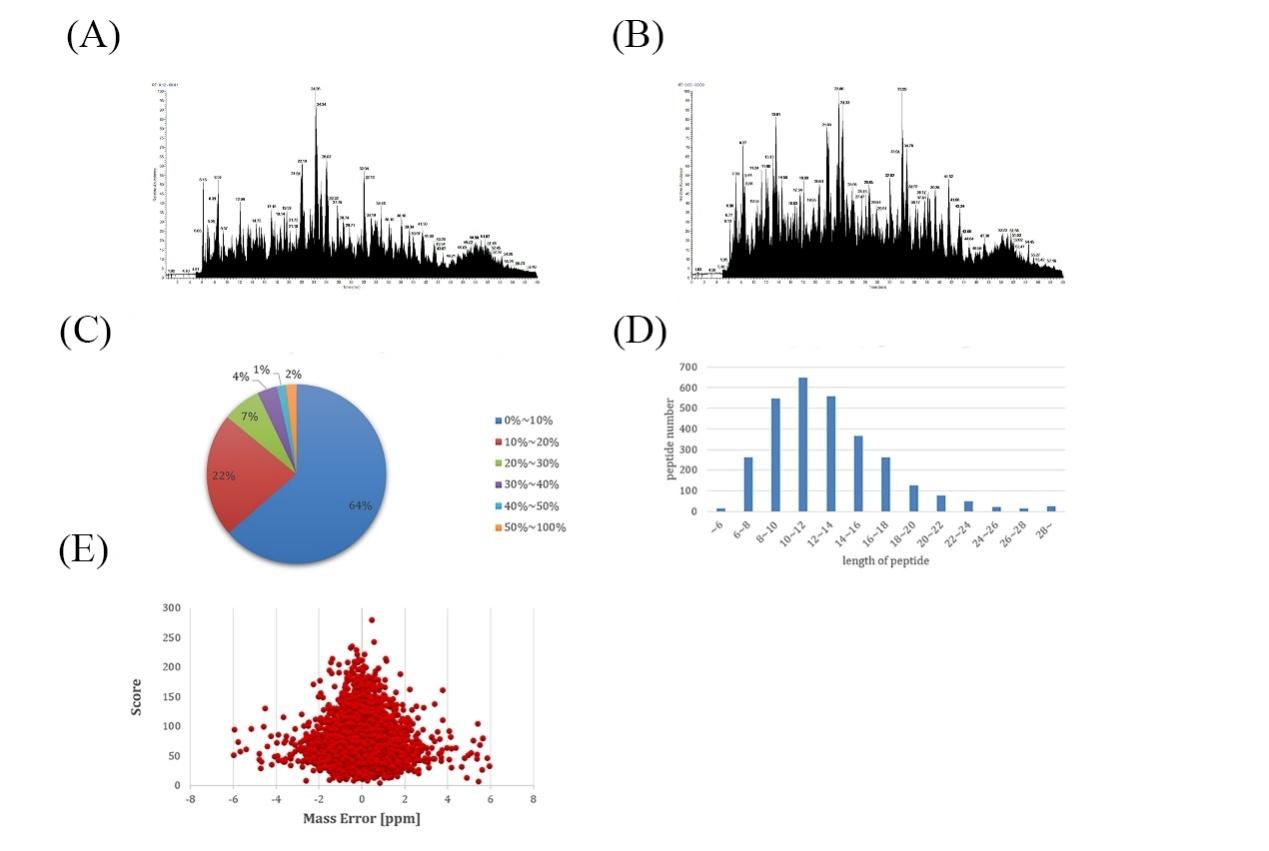


Figure S4. Evaluation of protein identification results.（A-B）The total ion flow chromatogram of protein samples in WT-H and WT-D; (C): Distribution of proteins’s sequence coverage; (D): peptide length distribution; (E): peptide matching error distribution）

Table 1 Primers used in the experiment

| Primer | Sequences（5’-3’） | Length（bp） | Function | Note |
| --- | --- | --- | --- | --- |
| up-*Achog1*-F | CCGCTCGAGCGAGCAGTTTTTCTGGTACTCCG | 32 bp | Amplification *Achog1-*L | The red label is the enzyme site of *Xho*Ⅰ |
| up-*Achog1*-R | CGCGGATCCTCGTGGCACGCACGAATTC | 28 bp | Amplification *Achog1-*L | The red label is the enzyme site of *BamH*Ⅰ |
| down-*Achog1*-F | CGCGGATCCATCCAGAATAGACAGATTTCTCTTTTGC | 37 bp | Amplification *Achog1-*R | The red label is the enzyme site of *Spe*Ⅰ |
| down-*Achog1*-R | GCTCTAGATGGCGGTGGATCGTCAT | 25 bp | Amplifiy *Achog1-*R | The red label is the enzyme site of *Xba*Ⅰ |
| qc-*Achog1*-F | GTCCAGCCTCGCGGTCCTC | 19bp | Amplification the full length of *Achog1* | The red label is the enzyme site of *Kpn*Ⅰ |
| qc-*Achog1*-R | TTAGGCGGCGAATGCCTGC | 19bp | Amplify the full length of *Achog1* | The red label is the enzyme site of *Hind*Ⅲ |
| *Achog1*-F | CGAGCAGTTTTTCTGGTACTCCG | 21 bp | Knockout strain verification |  |
| *Achog1-*R | TGGCGGTGGATCGTCAT | 25 bp |  |
| *Achog1-F1* | TTGTCGTAGATACTCCGTACT |  | Knockout strain verification |  |
| *Achog1-R1* | TGGGAATTGATATCGATGATGGGGT |  |  |  |
| *hyg*-F | TGGCAAACTGTGATGGACGA | 20bp | Knockout strain verification  Detection the copy numbe of hph | |
| *hyg-*R | RGGCGACCTCGTATTGGGAAT | 20bp |
| *GAPDH-*F | CTGCCGTATCGAGAAGGGTG | 20bp |
| *GAPDH-*R | GATGAAGTTGGGTTGAGGG | 19bp |
| SI65_05591-F | CACCAATATCTCATCGTCGCCTGTC | 25bp | RT-PCR |  |
| SI65_05591-R | CTTATCTGGGTTATCGGCACGGAAG | 25bp | RT-PCR |  |
| SI65_05592-F | GCGTGAGTCGGAAGCGTACATC | 24bp | RT-PCR |  |
| SI65_05592-R | GTGGTGGATGAATAGGCGGGAATG | 24bp | RT-PCR |  |
| SI65_05588-F | CCCTCACTCCCCAGACCTTCAC | 22bp | RT-PCR |  |
| SI65_05588-R | GGCGTAGTTGATGGCGACTTGG | 22bp | RT-PCR |  |
| SI65_07763-F | ATCTGTCGTCCCTGGGTCACTTC | 23bp | RT-PCR |  |
| SI65_07763-R | GCTGGATCTTCTCACACGCTTCC | 23bp | RT-PCR |  |
| SI65_10255-F | ACATTGCCTGCTGCGATGCTAC | 22bp | RT-PCR | |
| SI65_10225-R | TGACGAGACCACCCTCCTGTTG | 22bp | RT-PCR | |
| SI65_07477-F | TCAGTGACCAGTGCAAGCAGAAC | 23bp | RT-PCR | |
| SI65-07477-R | GGAAGTGCGACATTGACGAGACC | 23bp | RT-PCR | |

Table 2 Ct and Tm values of internal reference genes and exogenous genes in *△Achog1*

| Strains | GAPDH | | HYG | |
| --- | --- | --- | --- | --- |
|  | Ct value | TM（℃） | Ct value | TM（℃） |
| The deletion mutant of *Achog1* | 17.13±0.05 | 60 | 16.49±0.08 | 60 |
| The deletion mutant of *VeA* | 13.75±0.05 | 60 | 15.78±0.08 | 60 |
| NTC | N/A | N/A | N/A | N/A |
|  |  |  |  |  |

Table 3 The top 20 proteins with higher scores

| Protein IDs | Descriptions | Score | Functions |
| --- | --- | --- | --- |
| A0A1E3BEM7 | **HABP4_PAI-RBP1 domain-containing protein** | 323.31 | RNA binding |
| A0A1E3BMD2 | Uncharacterized protein | 311.02 | Unknown |
| A0A1E3BCJ0 | Uncharacterized protein | 304.57 | Unknown |
| A0A1E3BEX0 | RRM domain-containing protein | 215.19 | RNAbinding |
| A0A1E3B6Q8 | MIF4G-domain-containing protein | 207.27 | RNA/protein binging |
| A0A1E3BSR2 | Uncharacterized protein | 197.31 | Unknown |
| A0A1E3BN38 | **LsmAD-domain-containing protein** | 183.98 | translational regulation |
| A0A1E3B7G9 | Uncharacterized protein | 157.31 | Unknown |
| A0A1E3B5W0 | Uncharacterized protein | 151.69 | Unknown |
| A0A1E3BKS5 | ATP synthase subunit alpha | 145.63 | ATP binding |
| A0A1E3B193 | Multiprotein-bridging factor 1 | 138.84 | DNA binging |
| A0A1E3BPR5 | C2H2-type-domain-containing protein | 135.81 | Related to transcription, translation, mRNA trafficking, cytoskeleton organisation, epithelial development |
| A0A1E3B0L0 | Aldehyde dehydrogenase | 134.77 | [oxidoreductase activity](https://www.ebi.ac.uk/QuickGO/GTerm?id=GO:0016491) |
| A0A1E3BSA9 | 40S ribosomal protein S19 | 128.57 | [structural constituent of ribosome](https://www.ebi.ac.uk/QuickGO/GTerm?id=GO:0003735) |
| A0A1E3B4X0 | Cytochrome c | 125.38 | Heme biding |
| A0A1E3B957 | 6-phosphogluconate dehydrogenase | 124.48 | Create a salt bridge between monomers close to the substrate-binding site |
| A0A1E3B7H4 | RNB domain-containing protein | 123.01 | ribonuclease activity |
| A0A1E3BJ79 | WH1 domain-containing protein | 118.93 | Acting binding |
| A0A1E3BNV8 | D-xylose reductase | 117.03 | [NADP reductase activity](https://www.ebi.ac.uk/QuickGO/term/GO:0032866) |
| A0A1E3BSV0 | Eukaryotic translation initiation factor 3 subunite A | 115.81 | [translation initiation factor activity](https://www.ebi.ac.uk/QuickGO/term/GO:0003743) |

Table 4 Nine proteins validated by Yeast Two-Hybrid

| Proteins IDs | Name | Mol wight [KDa] | LFQ interesity WT-D | LFQ interesity WT-H |
| --- | --- | --- | --- | --- |
| A0A1E3B8J2 | Ste20 | 83.242 | 0 | 18220000 |
| A0A1E3BC73 | Ste7 | 59.268 | 0 | 13623000 |
| A0A1E3B873 | RodA | 13.493 | 0 | 23506000 |
| A0A1E3B0F0 | RodB | 14.322 | 0 | 12638000 |
| A0A1E3B0Z7 | Csp | 8.3708 | 0 | 1272600000 |
| A0A1E3BD42 | Bmh1 | 29.357 | 0 | 32053000 |
| A0A1E3BMS4 | Cla4 | 92.205 | 0 | 80041000 |
| A0A1E3BB62 | PhnA | 29.826 | 0 | 8023500 |
| A0A1E3BER4 | Sko1 | 54.752 | 0 | 19031000 |

Table 5 The quality statistics of filtered reads

| sample | clean_bases  (Gb) | error_rate  (%） | Q20  （%） | Q30  （%） | GC_pct  （%） |
| --- | --- | --- | --- | --- | --- |
| WT_1 | 5.98G | 0.03 | 97.57 | 93.58 | 55.14 |
| WT_2 | 6.15G | 0.03 | 97.5 | 93.44 | 54.68 |
| WT_3 | 6.26G | 0.03 | 97.57 | 93.5 | 54.88 |
| △*Achog1*_1 | 6.33G | 0.03 | 97.3 | 93.03 | 55.02 |
| △*Achog1*_2 | 6.23G | 0.03 | 97.21 | 92.83 | 55.1 |
| △*Achog1*_3 | 6.03G | 0.03 | 97.32 | 93.09 | 54.48 |

Table 6 The top 20 up-regulated genes in △*Achog1* vs wild type

| Top | GeneID | Length | WT | △*Achog1* | Log_2_FC  (△*Achog1*/WT) | | Description |
| --- | --- | --- | --- | --- | --- | --- | --- |
| 1 | gene-SI65_04725 | 207 | 248.38 | 18986.83 | 6.25 | Unknown | |
| 2 | gene-SI65_06822 | 162 | 0 | 6.69 | 5.25 | Unknow | |
| 3 | gene-SI65_05992 | 1122 | 0.30 | 12.25 | 5.16 | Reverse transcriptase (RNA-dependent DNA polymerase) | |
| 4 | gene-SI65_08155 | 780 | 0.304 | 10.64 | 4.96 | Unknown | |
| 5 | gene-SI65_09912 | 468 | 4737.57 | 136724.4 | 4.85 | Unknown | |
| 6 | novel.294 | 963 | 0.91 | 21.129 | 4.491 | Unknow | |
| 7 | novel.522 | 1030 | 2.52 | 44.82 | 4.147 | Unknow | |
| 8 | gene-SI65_01919 | 1617 | 1.21 | 17.38 | 3.79 | Low-affinity glucose transporter HXT3 | |
| 9 | gene-SI65_05766 | 888 | 66.71 | 880.70 | 3.72 | NADH-cytochrome b5 reductase 2 | |
| 10 | gene-SI65_09954 | 1725 | 3.55 | 42.56 | 3.59 | Phosphate-repressible phosphate permease pho-4 | |
| 11 | gene-SI65_05026 | 636 | 2.17 | 23.71 | 3.42 | Unknown | |
| 12 | gene-SI65_04726 | 690 | 1.82 | 19.79 | 3.40 | Unknown | |
| 13 | gene-SI65_06451 | 426 | 1.56 | 15.98 | 3.34 | Unknown | |
| 14 | gene-SI65_04687 | 675 | 21.20 | 208.70 | 3.29 | Unknown | |
| 15 | gene-SI65_05015 | 444 | 14.41 | 136.59 | 3.25 | Unknown | |
| 16 | gene-SI65_00200 | 555 | 8455.94 | 79608.93 | 3.23 | Cell wall protein phiA | |
| 17 | gene-SI65_03401 | 6510 | 7.07 | 66.45 | 3.20 | TPR and ankyrin repeat-containing protein 1 | |
| 18 | gene-SI65_03378 | 807 | 1.86 | 15.80 | 3.06 | FAD dependent oxidoreductase | |
| 19 | gene-SI65_02819 | 210 | 626.94 | 5219.63 | 3.05 | Unknown | |
| 20 | gene-SI65_08227 | 642 | 51.21 | 425.48 | 3.05 | Unknown | |

| Top | Gene ID | Length | WT | △*Achog1* | Log_2_FC  (△*Achog1/*WT) | Decrisption |
| --- | --- | --- | --- | --- | --- | --- |
| 1 | gene-SI65_01353 | 762 | 94.23 | 0 | -8.94 | Rossmann-fold NAD(P)H/NAD(P)(+) binding (NADB) domain. |
| 2 | gene-SI65_00161 | 1548 | 75.61 | 0 | -8.62 | High-affinity glucose transporter |
| 3 | gene-SI65_05422 | 1818 | 3610.94 | 16.19 | -7.802 | Catalase A |
| 4 | gene-SI65_05586 | 948 | 182.67 | 1.02 | -7.46 | Unknown |
| now5 | gene-SI65_02242 | 1089 | 972.97 | 5.89 | -7.37 | Unknown |
| 6 | gene-SI65_06672 | 2205 | 333.05 | 2.44 | -7.09 | Cytochrome P450 monooxygenase mpaDE |
| 7 | gene-SI65_03917 | 1212 | 50.12 | 0.34 | -7.07 | Protein kinase *dsk1* |
| 8 | gene-SI65_07257 | 759 | 3853.03 | 30.74 | -6.97 | SnoaL-like domain |
| 9 | gene-SI65_09338 | 315 | 19012.77 | 153.395 | -6.95 | Stress responsive A/B Barrel Domain |
| 10 | gene-SI65_04459 | 336 | 814.63 | 6.57 | -6.95 | Unknown |
| 11 | gene-SI65_02867 | 1518 | 21.39 | 0 | -6.80 | Vitamin B6 transporter *TPN1* |
| 12 | gene-SI65_00763 | 468 | 2927.17 | 26.60 | -6.78 | Multistep phosphorelay regulator 1 |
| 13 | gene-SI65_00446 | 1161 | 747.21 | 7.73 | -6.61 | Probable beta-glucosidase btgE |
| 14 | gene-SI65_04948 | 558 | 1129.43 | 12.96 | -6.45 | Unknown |
| 15 | gene-SI65_05163 | 1218 | 2166.74 | 24.77 | -6.45 | Unknown |
| 16 | gene-SI65_05592 | 1758 | 981.39 | 11.54 | -6.42 | Laccase *abr2* |
| 17 | gene-SI65_06408 | 1833 | 553.05 | 6.61 | -6.39 | Unknown |
| 18 | gene-SI65_04578 | 333 | 27.16 | 0.34 | -6.17 | Unknown |
| 19 | gene-SI65_05591 | 1455 | 684.41 | 9.56 | -6.16 | Multicopper oxidase *abr1* |
| 20 | gene-SI65_00361 | 780 | 12.56 | 0 | -6.037 | Sulfite efflux pump *SSU1* |

Table 7 The top 20 down-regulated genes in △*Achog1* strain verus wild type

Table S8 All the proteins identified in WT-H

| Protein IDs | Mol. weight [kDa] | Score | Descriptions |
| --- | --- | --- | --- |
| A0A1E3B5N1 | 20.474 | 80.065 | Uncharacterized protein |
| A0A1E3BTD1 | 68.169 | 65.008 | MICOS complex subunit MIC60 |
| A0A1E3BEU9 | 13.132 | 63.969 | Uncharacterized protein |
| A0A1E3BME7 | 27.849 | 62.83 | Probable electron transfer flavoprotein subunit beta |
| A0A1E3B2K0 | 70.737 | 62.17 | Succinate dehydrogenase [ubiquinone] flavoprotein |
| A0A1E3BRE8 | 30.761 | 60.011 | Eukaryotic translation initiation factor 3 subunit J |
| A0A1E3BMS4 | 92.205 | 56.766 | Non-specific serine/threonine protein kinase |
| A0A1E3BDE3 | 164.88 | 55.192 | Uncharacterized protein |
| A0A1E3BEA9 | 91.357 | 53.776 | Uncharacterized protein |
| A0A1E3BPB1 | 176.59 | 53.353 | Uncharacterized protein |
| A0A1E3BUA0 | 17.538 | 52.824 | Eukaryotic translation initiation factor 5A |
| A0A1E3BGD6 | 90.237 | 52.812 | Cysteinyl-tRNA synthetase |
| A0A1E3BHV7 | 619.61 | 52.675 | Uncharacterized protein |
| A0A1E3BG13 | 50.998 | 52.32 | J domain-containing protein |
| A0A1E3B0Z7 | 8.3708 | 49.64 | Conidiation-specific protein |
| A0A1E3BGS4 | 117.06 | 47.968 | Elongation factor 3 |
| A0A1E3BBW0 | 32.398 | 47.798 | Uncharacterized protein |
| A0A1E3BE23 | 103.85 | 45.599 | Uncharacterized protein |
| A0A1E3BJ21 | 31.587 | 45.285 | SAP domain-containing protein |
| A0A1E3BMW0 | 72.835 | 43.775 | Methionyl-tRNA synthetase |
| A0A1E3B4C0 | 145.7 | 43.356 | Uncharacterized protein |
| A0A1E3BFX8 | 71.541 | 38.852 | PWWP domain-containing protein |
| A0A1E3BJQ4 | 9.0397 | 38.497 | Uncharacterized protein |
| A0A1E3B275 | 76.913 | 38.228 | ERF-3 |
| A0A1E3BDB7 | 51.599 | 37.989 | Uncharacterized protein |
| A0A1E3B2M7 | 32.674 | 37.939 | Uncharacterized protein |
| A0A1E3BPN3 | 98.324 | 36.659 | Uncharacterized protein |
| A0A1E3B8C3 | 39.798 | 36.318 | Phospho-2-dehydro-3-deoxyheptonate aldolase |
| A0A1E3B595 | 44.136 | 35.168 | Uncharacterized protein |
| A0A1E3BGJ9 | 24.85 | 34.679 | Uncharacterized protein |
| A0A1E3BG83 | 80.709 | 34.643 | NADH-ubiquinone oxidoreductase |
| A0A1E3B510 | 60.93 | 33.898 | Uncharacterized protein |
| A0A1E3B6T4 | 14.572 | 33.014 | Uncharacterized protein |
| A0A1E3BML9 | 61.999 | 32.738 | Uncharacterized protein |
| A0A1E3B2L6 | 157.82 | 32.149 | Uncharacterized protein |
| A0A1E3BD90 | 23.798 | 32.044 | Endoplasmic reticulum transmembrane protein |
| A0A1E3BL30 | 55.729 | 31.989 | Uncharacterized protein |
| A0A1E3BKP9 | 68.42 | 31.273 | Cytochrome b5 heme-binding domain-containing protein |
| A0A1E3B485 | 59.699 | 31.231 | Peptidylprolyl isomerase |
| A0A1E3B1D0 | 68.897 | 30.919 | Lysophospholipase |
| A0A1E3B5G0 | 39.286 | 30.573 | Uncharacterized protein |
| A0A1E3BAC3 | 72.353 | 30.544 | Heat shock 70 kDa protein |
| A0A1E3BSV2 | 25.052 | 30.342 | Vacuolar-sorting protein snf7 |
| A0A1E3BLP3 | 21.333 | 30.165 | Uncharacterized protein |
| A0A1E3BHM8 | 29.79 | 28.373 | RRF domain-containing protein |
| A0A1E3BRA5 | 54.175 | 28.035 | Putative aldehyde dehydrogenase-like protein |
| A0A1E3BTA9 | 16.106 | 27.492 | Uncharacterized protein |
| A0A1E3BLM7 | 17.947 | 27.023 | mRNA stability protein |
| A0A1E3BIP0 | 53.413 | 26.612 | Alkaline protease 2 |
| A0A1E3BLT7 | 52.204 | 26.277 | Uncharacterized protein |
| A0A1E3B0E2 | 22.869 | 25.958 | Putative altered inheritance of mitochondria protein |
| A0A1E3BPR4 | 38.71 | 25.894 | Fructose-bisphosphatase |
| A0A1E3BN57 | 53.524 | 25.689 | KH domain-containing protein |
| A0A1E3B099 | 27.651 | 25.687 | DLH domain-containing protein |
| A0A1E3BQN1 | 46.979 | 25.446 | Aspartate aminotransferase |
| A0A1E3BN68 | 43.466 | 25.309 | Obg-like ATPase 1 |
| A0A1E3BNW4 | 31.109 | 25.112 | SUZ domain-containing protein |
| A0A1E3BA05 | 37.275 | 25.045 | Uncharacterized protein |
| A0A1E3BJK6 | 60.035 | 25.027 | Uncharacterized protein |
| A0A1E3B8P3 | 17.398 | 24.923 | Uncharacterized protein |
| A0A1E3B9P9 | 15.744 | 24.501 | Uncharacterized protein |
| A0A1E3BRD6 | 22.685 | 24.365 | Uncharacterized protein |
| A0A1E3B7Z5 | 29.903 | 24.294 | Uncharacterized protein |
| A0A1E3B795 | 124.05 | 24.282 | Uncharacterized protein |
| A0A1E3BEI1 | 79.813 | 24.22 | Catalase |
| A0A1E3BVM6 | 55.155 | 24.156 | UBX domain-containing protein |
| A0A1E3BCH8 | 11.435 | 24 | Uncharacterized protein |
| A0A1E3BJN9 | 77.853 | 23.956 | Serine/threonine-protein phosphatase 2A |
| A0A1E3B449 | 72.995 | 23.932 | Enhancer of mRNA-decapping protein |
| A0A1E3BLJ6 | 91.594 | 23.711 | HMG box domain-containing protein |
| A0A1E3BQ37 | 54.738 | 23.521 | But2 domain-containing protein |
| A0A1E3BJY1 | 80.169 | 23.385 | Uncharacterized protein |
| A0A1E3BAD1 | 22.259 | 23.283 | Uncharacterized protein |
| A0A1E3BEW4 | 10.171 | 23.158 | 60S ribosomal protein L43 |
| A0A1E3B6F9 | 31.748 | 22.898 | Putative enoyl-CoA hydratase, mitochondrial |
| A0A1E3B0B1 | 94.626 | 22.491 | Nitrogen regulatory protein areA |
| A0A1E3BHH7 | 28.756 | 22.386 | Proteasome subunit alpha type |
| A0A1E3BL95 | 58.702 | 22.122 | Csm1 domain-containing protein |
| A0A1E3BNJ7 | 30.039 | 21.343 | Mitochondrial outer membrane protein porin |
| A0A1E3BIG1 | 136.24 | 20.808 | Uncharacterized protein |
| A0A1E3BQQ8 | 9.7632 | 20.76 | NADH dehydrogenase [ubiquinone] 1 alpha subcomplex |
| A0A1E3B976 | 54.462 | 20.499 | Dihydrolipoyl dehydrogenase |
| A0A1E3BJE7 | 34.305 | 20.446 | Signal recognition particle receptor subunit beta |
| A0A1E3BCM0 | 272.1 | 20.372 | DUF4045 domain-containing protein |
| A0A1E3BJZ0 | 88.642 | 19.968 | HSF_DOMAIN domain-containing protein |
| A0A1E3BFV4 | 74.623 | 19.964 | CAP-Gly domain-containing protein |
| A0A1E3B4D6 | 24.48 | 19.9 | NADH dehydrogenase [ubiquinone] iron-sulfur protein |
| A0A1E3B9P1 | 32.867 | 19.754 | Uncharacterized protein |
| A0A1E3BDY9 | 89.739 | 19.683 | CID domain-containing protein |
| A0A1E3BK46 | 27.984 | 19.651 | Uncharacterized protein |
| A0A1E3BI18 | 89.914 | 19.606 | Uncharacterized protein |
| A0A1E3BHP2 | 41.564 | 19.431 | Actin, gamma |
| A0A1E3BNQ4 | 17.381 | 19.323 | 60S ribosomal protein L31 |
| A0A1E3BK92 | 37.706 | 19.271 | Transcriptional activator HAP2 |
| A0A1E3B361 | 145.42 | 19.239 | Cytokin_check_N domain-containing protein |
| A0A1E3BAA5 | 88.665 | 19.222 | Uncharacterized protein |
| A0A1E3B6T8 | 80.815 | 19.154 | Uncharacterized protein |
| A0A1E3B9J6 | 88.345 | 19.109 | Rho-GAP domain-containing protein |
| A0A1E3BC73 | 59.268 | 19.029 | Protein kinase domain-containing protein |
| A0A1E3BME4 | 53.148 | 18.982 | V-type proton ATPase subunit H |
| A0A1E3B527 | 151.21 | 18.933 | CAP-Gly domain-containing protein |
| A0A1E3B934 | 57.569 | 18.909 | PH domain-containing protein |
| A0A1E3B1K4 | 25.71 | 18.706 | V-type proton ATPase subunit E |
| A0A1E3B0J0 | 57.135 | 18.471 | RRM domain-containing protein |
| A0A1E3B8V0 | 32.499 | 18.344 | NADH-ubiquinone oxidoreductase |
| A0A1E3B6A5 | 20.816 | 18.338 | Translation machinery-associated protein 22 |
| A0A1E3BQQ7 | 24.402 | 18.331 | Mitochondrial peroxiredoxin PRX1 |
| A0A1E3BL68 | 78.435 | 18.318 | Glycerol-3-phosphate dehydrogenase |
| A0A1E3B9N9 | 27.846 | 18.303 | Uncharacterized protein |
| A0A1E3BGE0 | 13.437 | 18.153 | Uncharacterized protein |
| A0A1E3BCX1 | 26.817 | 18.033 | Uncharacterized protein |
| A0A1E3B1L1 | 53.206 | 18.002 | Aldehyde dehydrogenase |
| A0A1E3B1Z1 | 61.083 | 17.854 | Uncharacterized protein |
| A0A1E3B9S1 | 142.43 | 17.802 | TPR_REGION domain-containing protein |
| A0A1E3B3I5 | 15.475 | 17.792 | Uncharacterized protein |
| A0A1E3B983 | 87.461 | 17.722 | Uncharacterized protein |
| A0A1E3BMF9 | 65.605 | 17.602 | Vacuolar protein sorting-associated protein 17 |
| A0A1E3BMA6 | 116.64 | 17.571 | Non-specific serine/threonine protein kinase |
| A0A1E3BKS1 | 29.073 | 17.565 | 60S ribosomal protein L7 |
| A0A1E3BHQ3 | 49.257 | 17.561 | RRM domain-containing protein |
| A0A1E3BQ13 | 86.94 | 17.54 | RNA helicase |
| A0A1E3BQ56 | 61.178 | 17.404 | Carboxypeptidase |
| A0A1E3BI73 | 72.055 | 17.379 | Fimbrin |
| A0A1E3BCA7 | 68.999 | 17.317 | PCI domain-containing protein |
| A0A1E3BBZ9 | 11.148 | 16.857 | Uncharacterized protein |
| A0A1E3BLR3 | 20.019 | 16.603 | Uncharacterized protein |
| A0A1E3BPP5 | 25.899 | 16.587 | Uncharacterized protein |
| A0A1E3BNH3 | 39.441 | 16.457 | 3-isopropylmalate dehydrogenase |
| A0A1E3BHQ7 | 48.43 | 16.28 | Uncharacterized protein |
| A0A1E3BM23 | 49.398 | 16.075 | Pre-mRNA-splicing factor prp46 |
| A0A1E3BFQ9 | 119.8 | 16.065 | Valyl-tRNA synthetase |
| A0A1E3BG99 | 32.834 | 16.061 | Uncharacterized protein |
| A0A1E3B2I8 | 16.246 | 15.934 | Uncharacterized protein |
| A0A1E3BBN6 | 94.628 | 15.558 | VOC domain-containing protein |
| A0A1E3BNT2 | 10.794 | 15.542 | NADH dehydrogenase [ubiquinone] 1 beta subcomplex |
| A0A1E3BM40 | 79.957 | 15.466 | PUM-HD domain-containing protein |
| A0A1E3B633 | 102.57 | 15.205 | Heat shock protein hsp98 |
| A0A1E3B833 | 42.858 | 15.142 | Putative aryl-alcohol dehydrogenase AAD14 |
| A0A1E3BFJ7 | 42.689 | 15.129 | Pre-mRNA-splicing factor slt11 |
| A0A1E3BBN4 | 28.464 | 15.001 | PP28 domain-containing protein |
| A0A1E3BP07 | 70.377 | 14.858 | Uncharacterized protein |
| A0A1E3B6R0 | 29.837 | 14.855 | Uncharacterized protein |
| A0A1E3BRH5 | 50.163 | 14.831 | Uncharacterized protein |
| A0A1E3BN45 | 52.684 | 14.82 | RRM domain-containing protein |
| A0A1E3BA17 | 39.836 | 14.68 | S-(hydroxymethyl)glutathione dehydrogenase |
| A0A1E3BPL6 | 126.7 | 14.625 | Leucyl-tRNA synthetase |
| A0A1E3BHI8 | 20.487 | 14.604 | Histone H3 |
| A0A1E3B944 | 59.833 | 14.552 | Arf-GAP domain-containing protein |
| A0A1E3BLR7 | 28.892 | 14.429 | Uncharacterized protein |
| A0A1E3BD50 | 11.734 | 14.368 | Complex I-B22 |
| A0A1E3BD97 | 9.993 | 14.217 | 40S ribosomal protein S21 |
| A0A1E3B7E6 | 41.003 | 14.053 | WH2 domain-containing protein |
| A0A1E3BNQ0 | 13.828 | 14.053 | SCP2 domain-containing protein |
| A0A1E3BLH4 | 15.265 | 14.039 | Uncharacterized protein |
| A0A1E3BR17 | 110.96 | 13.954 | Protein kinase domain-containing protein |
| A0A1E3BG60 | 31.785 | 13.942 | Uncharacterized protein |
| A0A1E3B8L3 | 21.833 | 13.938 | Uncharacterized protein |
| A0A1E3BQR8 | 82.825 | 13.89 | Uncharacterized protein |
| A0A1E3BR77 | 74.764 | 13.871 | DUF2013 domain-containing protein |
| A0A1E3BHD4 | 22.72 | 13.828 | Mso1_Sec1_bdg domain-containing protein |
| A0A1E3BPP3 | 158.71 | 13.824 | Uncharacterized protein |
| A0A1E3BPP9 | 228.89 | 13.798 | TPR_MLP1_2 domain-containing protein |
| A0A1E3B4T7 | 79.866 | 13.672 | Uncharacterized protein |
| A0A1E3BGW5 | 14.878 | 13.636 | 60S ribosomal protein L26-1 |
| A0A1E3BNT6 | 24.692 | 13.609 | GTP-binding nuclear protein |
| A0A1E3BS40 | 31.321 | 13.553 | Mannose-P-dolichol utilization defect 1 protein |
| A0A1E3B2W4 | 132.14 | 13.513 | Cytoskeletal adapter protein sagA |
| A0A1E3BCF4 | 13.284 | 13.508 | Mitochondrial zinc maintenance protein 1 |
| A0A1E3B5G5 | 74.638 | 13.462 | Glutamine--tRNA ligase |
| A0A1E3BN34 | 55.941 | 13.442 | Uncharacterized protein |
| A0A1E3BMS3 | 21.008 | 13.283 | Uncharacterized protein |
| A0A1E3B382 | 11.88 | 13.223 | Stress-response A/B barrel domain-containing protein |
| A0A1E3B9J9 | 10.278 | 13.202 | Mitochondrial import inner membrane translocase |
| A0A1E3BJK1 | 70.59 | 13.178 | Uncharacterized protein |
| A0A1E3BSI0 | 22.058 | 13.172 | Uncharacterized protein |
| A0A1E3BNP3 | 17.901 | 13.146 | 40S ribosomal protein S18 |
| A0A1E3BR46 | 43.361 | 13.061 | 3-ketoacyl-CoA thiolase, peroxisomal |
| A0A1E3BH76 | 92.351 | 13.06 | Uncharacterized protein |
| A0A1E3BKG0 | 137.48 | 12.995 | Class I unconventional myosin |
| A0A1E3BIU2 | 16.753 | 12.944 | Nucleoside diphosphate kinase |
| A0A1E3BLH2 | 143.1 | 12.897 | IPPc domain-containing protein |
| A0A1E3B0D7 | 57.655 | 12.851 | zf-C2HC5 domain-containing protein |
| A0A1E3BS22 | 26.99 | 12.742 | Putative oxidoreductase |
| A0A1E3BMM5 | 43.077 | 12.719 | COP9 signalosome complex subunit 6 |
| A0A1E3BIH0 | 43.298 | 12.685 | Septin spn3 |
| A0A1E3BGG2 | 16.815 | 12.666 | 40S ribosomal protein S13 |
| A0A1E3BD37 | 60.346 | 12.661 | Mitochondrial import inner membrane translocase |
| A0A1E3BE40 | 138.72 | 12.637 | Clustered mitochondria protein homolog |
| A0A1E3BPJ2 | 15.802 | 12.62 | NADH dehydrogenase [ubiquinone] 1 alpha subcomplex |
| A0A1E3BK53 | 21.045 | 12.61 | Uncharacterized protein |
| A0A1E3BIV7 | 109.19 | 12.554 | Probable alpha/beta-glucosidase agdC |
| A0A1E3BQS5 | 79.412 | 12.553 | mRNA-capping enzyme subunit beta |
| A0A1E3BMY5 | 97.36 | 12.511 | pH-response regulator protein palA |
| A0A1E3BII4 | 50.748 | 12.503 | DUF2040 domain-containing protein |
| A0A1E3BAU4 | 41.132 | 12.468 | Amidohydro-rel domain-containing protein |
| A0A1E3BMK9 | 76.604 | 12.446 | U3 small nucleolar ribonucleoprotein protein MPP10 |
| A0A1E3BK75 | 59.906 | 12.443 | Uncharacterized protein |
| A0A1E3BKG7 | 8.7215 | 12.372 | Uncharacterized protein |
| A0A1E3BB35 | 119.52 | 12.372 | Nuclear pore complex protein |
| A0A1E3BPH0 | 57.744 | 12.29 | 1,3-beta-glucanosyltransferase |
| A0A1E3BQ09 | 36.732 | 12.288 | Aha1_N domain-containing protein |
| A0A1E3BIJ2 | 140.37 | 12.282 | Uncharacterized protein |
| A0A1E3B9P3 | 58.479 | 12.226 | CCT-epsilon |
| A0A1E3BPL7 | 15.026 | 12.221 | Hit family protein |
| A0A1E3BEC6 | 52.113 | 12.201 | Senescence domain-containing protein |
| A0A1E3BSE0 | 13.185 | 12.15 | Peptidyl-prolyl cis-trans isomerase |
| A0A1E3BGG4 | 31.148 | 12.144 | S-formylglutathione hydrolase |
| A0A1E3BGY9 | 60.709 | 12.138 | Malate synthase |
| A0A1E3BHB6 | 24.775 | 12.123 | cwf21 domain-containing protein |
| A0A1E3B1V4 | 52.081 | 12.09 | Uncharacterized protein |
| A0A1E3B3G6 | 42.58 | 11.945 | Uncharacterized protein |
| A0A1E3BT06 | 100.67 | 11.944 | DNA topoisomerase I |
| A0A1E3BM65 | 53.675 | 11.918 | Protein phosphatase PP2A regulatory subunit B |
| A0A1E3BGJ7 | 36.536 | 11.846 | Probable electron transfer flavoprotein subunit |
| A0A1E3B832 | 165.87 | 11.819 | Structural maintenance of chromosomes protein |
| A0A1E3B9Y5 | 7.7399 | 11.798 | 40S ribosomal protein S28 |
| A0A1E3BET5 | 44.728 | 11.782 | L-type lectin-like domain-containing protein |
| A0A1E3B5Y9 | 17.082 | 11.776 | Jacalin-type lectin domain-containing protein |
| A0A1E3B3F9 | 130.17 | 11.761 | Exocyst complex component Sec8 |
| A0A1E3BAU9 | 28.257 | 11.715 | Glyoxalase II |
| A0A1E3BAW0 | 34.947 | 11.708 | PKS_ER domain-containing protein |
| A0A1E3BK74 | 11.712 | 11.694 | NADH-ubiquinone oxidoreductase 12 kDa subunit |
| A0A1E3BER4 | 54.752 | 11.692 | BZIP domain-containing protein |
| A0A1E3BEL8 | 122.65 | 11.681 | Uncharacterized protein |
| A0A1E3BAT9 | 100.79 | 11.666 | CULLIN_2 domain-containing protein |
| A0A1E3BH09 | 36.272 | 11.625 | Uncharacterized protein |
| A0A1E3BRJ4 | 48.608 | 11.622 | Uncharacterized protein |
| A0A1E3BAI5 | 26.275 | 11.591 | RanBD1 domain-containing protein |
| A0A1E3B366 | 82.936 | 11.586 | Cytochrome c domain-containing protein |
| A0A1E3BQU8 | 69.546 | 11.509 | Lysophospholipase |
| A0A1E3BA91 | 138.05 | 11.363 | NUC173 domain-containing protein |
| A0A1E3BBH6 | 56.047 | 11.361 | RNA helicase |
| A0A1E3B0F4 | 57.289 | 11.323 | Uncharacterized protein |
| A0A1E3BEU4 | 117.44 | 11.321 | Uncharacterized protein |
| A0A1E3B549 | 41.614 | 11.293 | Translocation protein SEC62 |
| A0A1E3BJY3 | 65.274 | 11.265 | Uncharacterized protein |
| A0A1E3BPT3 | 131.69 | 11.236 | Uncharacterized protein |
| A0A1E3BML7 | 31.66 | 11.195 | Proteasome subunit alpha type |
| A0A1E3BSY4 | 35.206 | 11.157 | HMG box domain-containing protein |
| A0A1E3BBL9 | 81.474 | 11.113 | LisH domain-containing protein |
| A0A1E3BB42 | 62.718 | 11.072 | Uncharacterized protein |
| A0A1E3BEJ5 | 59.956 | 11.062 | VWFA domain-containing protein |
| A0A1E3B663 | 57.132 | 11.048 | Coatomer subunit delta |
| A0A1E3BMT6 | 27.501 | 10.957 | 60S ribosomal protein L2 |
| A0A1E3B958 | 22.741 | 10.878 | 60S ribosomal protein L6-B |
| A0A1E3BCT4 | 39.521 | 10.818 | WD_REPEATS_REGION domain-containing protein |
| A0A1E3B8A4 | 69.819 | 10.596 | C2 domain-containing protein |
| A0A1E3BFL1 | 39.819 | 10.569 | Uncharacterized protein |
| A0A1E3B581 | 111.94 | 10.33 | Uncharacterized protein |
| A0A1E3BRZ3 | 11.527 | 10.328 | Uncharacterized protein |
| A0A1E3B093 | 39.01 | 10.294 | TauD domain-containing protein |
| A0A1E3B996 | 58.065 | 10.235 | Putative alanine aminotransferase, mitochondrial |
| A0A1E3BET9 | 50.598 | 9.9514 | Uncharacterized protein |
| A0A1E3BR18 | 69.683 | 9.6017 | Calponin-homology (CH) domain-containing protein |
| A0A1E3B247 | 31.369 | 9.3749 | Uncharacterized protein |
| A0A1E3BC57 | 84.494 | 9.242 | Uncharacterized protein |
| A0A1E3B929 | 71.062 | 9.2343 | Uncharacterized protein |
| A0A1E3B562 | 27.405 | 9.2058 | Uncharacterized protein |
| A0A1E3B285 | 20.578 | 9.0622 | DUF5353 domain-containing protein |
| A0A1E3BIK4 | 62.007 | 9.0171 | Putative methylcrotonoyl-CoA carboxylase beta chain |
| A0A1E3BTE5 | 73.002 | 8.9811 | Uncharacterized protein |
| A0A1E3B6X4 | 99.56 | 8.9021 | Uncharacterized protein |
| A0A1E3B182 | 33.526 | 8.8925 | eIF2B_5 domain-containing protein |
| A0A1E3B8D5 | 213.21 | 8.8617 | Dynamin-binding protein |
| A0A1E3BGB7 | 63.181 | 8.8571 | Xylulose kinase |
| A0A1E3B2D0 | 56.895 | 8.8418 | 1,3-beta-glucanosyltransferase |
| A0A1E3BRZ6 | 58.317 | 8.8353 | Pyruvate kinase |
| A0A1E3BHX7 | 40.19 | 8.8278 | Ribosomal RNA-processing protein |
| A0A1E3BC40 | 18.127 | 8.7728 | 60S ribosomal protein L21-A |
| A0A1E3BNF4 | 78.705 | 8.7583 | Zn(2)-C6 fungal-type domain-containing protein |
| A0A1E3B213 | 61.399 | 8.6839 | Uncharacterized protein |
| A0A1E3BF38 | 200.04 | 8.6131 | Uncharacterized protein |
| A0A1E3BT26 | 21.67 | 8.5365 | Minor allergen Alt a 7 |
| A0A1E3B6R8 | 157.21 | 8.535 | Protein kinase domain-containing protein |
| A0A1E3BDY2 | 28.551 | 8.44 | Uncharacterized protein |
| A0A1E3B6F3 | 7.7875 | 8.3606 | Uncharacterized protein |
| A0A1E3BQ99 | 25.889 | 8.297 | Clathrin light chain |
| A0A1E3B6S9 | 15.837 | 8.2498 | 40S ribosomal protein S16 |
| A0A1E3BR08 | 29.525 | 8.2285 | 40S ribosomal protein S3 |
| A0A1E3B3Y0 | 162.63 | 8.2076 | Uncharacterized protein |
| A0A1E3BLQ7 | 92.075 | 8.1999 | RNA helicase |
| A0A1E3BT24 | 40.947 | 8.1723 | APH domain-containing protein |
| A0A1E3BL01 | 30.273 | 8.1032 | Pyridoxal phosphate homeostasis protein |
| A0A1E3BNU7 | 135.22 | 8.0699 | RanBD1 domain-containing protein |
| A0A1E3B7I4 | 41.656 | 7.9412 | Isocitrate dehydrogenase [NAD] subunit, mitochondrial |
| A0A1E3BMT7 | 85.891 | 7.8784 | Uncharacterized protein |
| A0A1E3BI60 | 51.8 | 7.8546 | Nickel/cobalt efflux system |
| A0A1E3BTZ2 | 11.468 | 7.8344 | Uncharacterized protein |
| A0A1E3BN43 | 30.926 | 7.7994 | t-SNARE coiled-coil homology domain-containing protein |
| A0A1E3B3P7 | 103.24 | 7.7994 | Autophagy-related protein 13 |
| A0A1E3B294 | 50.493 | 7.7522 | C2H2-type domain-containing protein |
| A0A1E3BL31 | 59.921 | 7.6613 | Amidase |
| A0A1E3BIV5 | 27.186 | 7.6444 | Signal peptidase subunit 3 |
| A0A1E3B8H1 | 15.536 | 7.5733 | Mitochondrial import inner membrane translocase subunit TIM16 |
| A0A1E3BDD8 | 43.715 | 7.5563 | Uncharacterized protein |
| A0A1E3BAM8 | 28.242 | 7.5499 | V-type proton ATPase subunit D |
| A0A1E3B857 | 159 | 7.545 | Actin cytoskeleton-regulatory complex protein PAN1 |
| A0A1E3BHJ3 | 43.2 | 7.5347 | Cystathionine gamma-lyase |
| A0A1E3BMS0 | 30.578 | 7.5283 | Uncharacterized protein |
| A0A1E3BR41 | 68.797 | 7.4997 | J domain-containing protein |
| A0A1E3B5M0 | 9.5162 | 7.4649 | Uncharacterized protein |
| A0A1E3BIN9 | 29.185 | 7.4634 | Uncharacterized protein |
| A0A1E3BF58 | 60.035 | 7.3552 | Uncharacterized protein |
| A0A1E3B0B5 | 237.83 | 7.3265 | Uncharacterized protein |
| A0A1E3BNT1 | 48.305 | 7.3058 | Uncharacterized protein |
| A0A1E3BT19 | 50.104 | 7.2182 | Casein kinase I |
| A0A1E3BMQ9 | 84.946 | 7.2118 | Uncharacterized protein |
| A0A1E3BNR2 | 119.41 | 7.2002 | Oxoglutarate dehydrogenase (succinyl-transferring) |
| A0A1E3BNL5 | 12.668 | 7.1406 | SBDS domain-containing protein |
| A0A1E3BQL5 | 97.268 | 7.1258 | Rab-GAP TBC domain-containing protein |
| A0A1E3B2S4 | 36.735 | 7.1032 | Arp2/3 complex 34 kDa subunit |
| A0A1E3BQJ6 | 77.389 | 7.0992 | Uncharacterized protein |
| A0A1E3BL39 | 56.319 | 7.092 | Omp85 domain-containing protein |
| A0A1E3BJ19 | 81.705 | 7.0879 | Glutamine-dependent NAD(+) synthetase |
| A0A1E3BBF9 | 66.414 | 7.0816 | FAD-binding PCMH-type domain-containing protein |
| A0A1E3BFZ0 | 107.62 | 7.0636 | Alanine--tRNA ligase |
| A0A1E3BEC3 | 33.199 | 7.0456 | Uncharacterized protein |
| A0A1E3BJV7 | 18.181 | 7.023 | Uncharacterized protein |
| A0A1E3BAN8 | 118.47 | 7.0138 | Uncharacterized protein |
| A0A1E3BSE4 | 18.201 | 7.0118 | Dolichyl-diphosphooligosaccharide--protein glycosyltransferase subunit OST2 |
| A0A1E3BNW6 | 60.632 | 7.0027 | Carboxylic ester hydrolase |
| A0A1E3BEX5 | 49.463 | 6.981 | Uncharacterized protein |
| A0A1E3BCS2 | 26.934 | 6.9795 | Uncharacterized protein |
| A0A1E3B4D5 | 36.246 | 6.9749 | Deoxyhypusine hydroxylase |
| A0A1E3BFT8 | 143.19 | 6.974 | Transcription initiation factor TFIID subunit 2 |
| A0A1E3BPM8 | 103.01 | 6.9616 | Vac14_Fig4_bd domain-containing protein |
| A0A1E3BNE4 | 201.03 | 6.9267 | DNA topoisomerase 2 |
| A0A1E3B4M1 | 50.06 | 6.9069 | SHNi-TPR domain-containing protein |
| A0A1E3BK58 | 27.398 | 6.8771 | Ribulose-phosphate 3-epimerase |
| A0A1E3B846 | 125.01 | 6.8699 | Uncharacterized protein |
| A0A1E3BKR0 | 99.87 | 6.8679 | Protein kinase domain-containing protein |
| A0A1E3BPE8 | 126.58 | 6.8628 | ISWI chromatin-remodeling complex ATPase ISW2 |
| A0A1E3B848 | 56.579 | 6.8619 | Ribosomal RNA-processing protein |
| A0A1E3BP20 | 58.814 | 6.8465 | PH domain-containing protein |
| A0A1E3B3S2 | 76.369 | 6.8436 | DUF4110 domain-containing protein |
| A0A1E3BDN3 | 13.133 | 6.8399 | Uncharacterized protein |
| A0A1E3B1J1 | 20.926 | 6.8336 | NEDD8-conjugating enzyme UBC12 |
| A0A1E3BJM8 | 10.673 | 6.8169 | Uncharacterized protein |
| A0A1E3BF54 | 73.336 | 6.8131 | TGc domain-containing protein |
| A0A1E3BI82 | 32.264 | 6.806 | Uncharacterized protein |
| A0A1E3BLA3 | 84.866 | 6.8019 | Uncharacterized protein |
| A0A1E3BTD2 | 65.932 | 6.7996 | RNA-binding protein VTS1 |
| A0A1E3B837 | 101.6 | 6.7905 | Kinesin-like protein |
| A0A1E3B6K5 | 66.881 | 6.7693 | Plus3 domain-containing protein |
| A0A1E3B2Z8 | 59.224 | 6.754 | Uncharacterized protein |
| A0A1E3B8Q3 | 40.811 | 6.7125 | 3(2),5-bisphosphate nucleotidase |
| A0A1E3BQ96 | 39.833 | 6.6963 | UDG domain-containing protein |
| A0A1E3BB72 | 15.923 | 6.6953 | Uncharacterized protein |
| A0A1E3BLT4 | 44.664 | 6.6902 | Uncharacterized protein |
| A0A1E3B5G2 | 81.905 | 6.6869 | U6 snRNA-associated Sm-like protein LSm6 |
| A0A1E3B3H8 | 68.125 | 6.6865 | Aminoacylproline aminopeptidase |
| A0A1E3B637 | 14.668 | 6.6714 | Uncharacterized protein |
| A0A1E3BM28 | 43.556 | 6.6501 | V-type proton ATPase subunit C |
| A0A1E3BIS1 | 31.367 | 6.6442 | Uncharacterized protein |
| A0A1E3BGS6 | 45.559 | 6.6395 | Phosphatidylinositol transfer protein SFH5 |
| A0A1E3BL33 | 120.68 | 6.6309 | Uncharacterized protein |
| A0A1E3BDB1 | 13.107 | 6.6166 | 60S ribosomal protein L34-B |
| A0A1E3B3Y8 | 9.9704 | 6.608 | Sm protein F |
| A0A1E3BC81 | 90.135 | 6.6069 | Uncharacterized protein |
| A0A1E3BH91 | 205.27 | 6.5897 | Protein transport protein sec16 |
| A0A1E3BMB4 | 63.951 | 6.5813 | Uncharacterized protein |
| A0A1E3BC43 | 16.776 | 6.572 | 60S ribosomal protein L27a |
| A0A1E3BLK4 | 44.715 | 6.5703 | Phosphoglycerate kinase |
| A0A1E3BPR3 | 33.294 | 6.5653 | Transcription initiation factor IIE subunit beta |
| A0A1E3B8P6 | 39.003 | 6.5609 | WLM domain-containing protein |
| A0A1E3B1Z0 | 80.781 | 6.5601 | Uncharacterized protein |
| A0A1E3BIC5 | 49.997 | 6.5566 | Uncharacterized protein |
| A0A1E3BL64 | 47.573 | 6.5554 | Succinate--CoA ligase [ADP-forming] subunit beta, mitochondrial |
| A0A1E3BFD1 | 55.001 | 6.529 | Cytochrome b2, mitochondrial |
| A0A1E3B0Y0 | 51.281 | 6.527 | Acetyltransferase component of pyruvate dehydrogenase complex |
| A0A1E3B236 | 33.62 | 6.5197 | Uncharacterized protein |
| A0A1E3BQM6 | 23.992 | 6.5186 | Uncharacterized protein |
| A0A1E3BRC2 | 75.035 | 6.5183 | Uncharacterized protein |
| A0A1E3BK42 | 32.898 | 6.5167 | Glutaredoxin domain-containing protein |
| A0A1E3BTU0 | 20.009 | 6.516 | 60S ribosomal protein L11 |
| A0A1E3BMU3 | 54.216 | 6.5119 | Uncharacterized protein |
| A0A1E3BCW6 | 111.94 | 6.5069 | Protein kinase domain-containing protein |
| A0A1E3B4L1 | 74.047 | 6.4973 | Transcription activator of gluconeogenesis acuK |
| A0A1E3B273 | 87.05 | 6.4845 | Tr-type G domain-containing protein |
| A0A1E3B9S4 | 21.312 | 6.48 | ER membrane protein complex subunit 4 |
| A0A1E3BRL2 | 20.552 | 6.4788 | Inosine triphosphate pyrophosphatase |
| A0A1E3BBX2 | 8.9385 | 6.4723 | 40S ribosomal protein S27 |
| A0A1E3BSQ6 | 35.864 | 6.4614 | NADH-cytochrome b5 reductase |
| A0A1E3B7P6 | 13.022 | 6.4558 | 40S ribosomal protein S20 |
| A0A1E3BMH7 | 61.618 | 6.4541 | Uncharacterized protein |
| A0A1E3BSW1 | 58.412 | 6.447 | Uncharacterized protein |
| A0A1E3BQN7 | 42.96 | 6.4424 | Uncharacterized protein |
| A0A1E3BRG8 | 44.608 | 6.4226 | J domain-containing protein |
| A0A1E3BIT1 | 65.187 | 6.4216 | Putative dihydroxy-acid dehydratase, mitochondrial |
| A0A1E3BJZ8 | 65.823 | 6.4151 | Uncharacterized protein |
| A0A1E3BPE4 | 55.848 | 6.4112 | Succinyl-CoA:3-ketoacid-coenzyme A transferase |
| A0A1E3B765 | 48.601 | 6.4093 | Aspartate transaminase |
| A0A1E3BAQ6 | 38.309 | 6.4073 | Fe2OG dioxygenase domain-containing protein |
| A0A1E3BD69 | 61.185 | 6.4009 | Uncharacterized protein |
| A0A1E3BL17 | 64.445 | 6.4008 | HSF_DOMAIN domain-containing protein |
| A0A1E3BNL8 | 35.788 | 6.3925 | CN hydrolase domain-containing protein |
| A0A1E3AZY4 | 36.054 | 6.3875 | CCHC-type domain-containing protein |
| A0A1E3BPE3 | 65.08 | 6.3813 | Uncharacterized protein |
| A0A1E3BSE3 | 45.113 | 6.3689 | Uncharacterized protein |
| A0A1E3B271 | 79.954 | 6.3685 | Uncharacterized protein |
| A0A1E3B6G8 | 52.266 | 6.3628 | Rab GDP dissociation inhibitor |
| A0A1E3BQX3 | 89.14 | 6.3614 | DUF3074 domain-containing protein |
| A0A1E3BM48 | 23.806 | 6.3513 | Adenylyl-sulfate kinase |
| A0A1E3BJW1 | 76.154 | 6.3489 | H(+)-transporting two-sector ATPase |
| A0A1E3BKV9 | 12.869 | 6.3418 | Peptidylprolyl isomerase |
| A0A1E3B9V2 | 55.573 | 6.3387 | Protein arginine methyltransferase NDUFAF7 |
| A0A1E3BJI8 | 122.25 | 6.336 | Uncharacterized protein |
| A0A1E3B3D1 | 20.493 | 6.3254 | Putative glutathione-dependent formaldehyde-activating enzyme |
| A0A1E3B787 | 48.27 | 6.3252 | Elongation factor Tu |
| A0A1E3B3Y7 | 41.584 | 6.3227 | RRM domain-containing protein |
| A0A1E3B4X7 | 64.628 | 6.3194 | Uncharacterized protein |
| A0A1E3B6K8 | 41.697 | 6.317 | KRR1 small subunit processome component |
| A0A1E3BN89 | 31.357 | 6.3154 | 37S ribosomal protein S25, mitochondrial |
| A0A1E3BBE9 | 13.635 | 6.3033 | Uncharacterized protein |
| A0A1E3BUJ3 | 39.648 | 6.2915 | Proliferating cell nuclear antigen |
| A0A1E3BQ16 | 98.408 | 6.2889 | Uncharacterized protein |
| A0A1E3B756 | 145.42 | 6.2722 | Uncharacterized protein |
| A0A1E3BEF2 | 192.73 | 6.2597 | Carrier domain-containing protein |
| A0A1E3B8N3 | 23.853 | 6.258 | Uridylate kinase |
| A0A1E3BBI3 | 27.671 | 6.2564 | Uncharacterized protein |
| A0A1E3B8T0 | 72.342 | 6.2496 | Rho-GAP domain-containing protein |
| A0A1E3BBL3 | 37.503 | 6.2417 | Uncharacterized protein |
| A0A1E3BSQ3 | 20.255 | 6.2362 | 60S ribosomal protein |
| A0A1E3B9Y9 | 28.973 | 6.2347 | NAD(P)-bd_dom domain-containing protein |
| A0A1E3B859 | 92.927 | 6.2327 | Condensin complex subunit 2 |
| A0A1E3B873 | 13.493 | 6.2239 | Hydrophobin |
| A0A1E3B564 | 40.246 | 6.2225 | PAPA-1 domain-containing protein |
| A0A1E3B8J2 | 83.242 | 6.2001 | Protein kinase domain-containing protein |
| A0A1E3B8Z6 | 46.733 | 6.1992 | IU_nuc_hydro domain-containing protein |
| A0A1E3BGC4 | 99.395 | 6.1974 | UBA domain-containing protein |
| A0A1E3BU12 | 68.63 | 6.1862 | ENTH domain-containing protein |
| A0A1E3B232 | 26.98 | 6.1824 | Uncharacterized protein |
| A0A1E3BAP3 | 44.695 | 6.1746 | SH3 domain-containing protein |
| A0A1E3BRF7 | 18.798 | 6.1614 | PRA1 family protein |
| A0A1E3BI61 | 108.23 | 6.1539 | DNA helicase |
| A0A1E3B4M2 | 37.744 | 6.1537 | Uncharacterized protein |
| A0A1E3BA41 | 81.575 | 6.148 | Uncharacterized protein |
| A0A1E3BPD7 | 34.973 | 6.1376 | Guanine nucleotide-binding protein subunit beta-like protein |
| A0A1E3BA44 | 25.229 | 6.1338 | Cytochrome b-c1 complex subunit Rieske |
| A0A1E3BTR9 | 26.927 | 6.1216 | Uncharacterized protein |
| A0A1E3BB62 | 29.826 | 6.1154 | Phosducin domain-containing protein |
| A0A1E3BK09 | 29.776 | 6.1096 | Proteasome subunit alpha type |
| A0A1E3B473 | 116.03 | 6.0993 | Chitinase |
| A0A1E3BMG2 | 14.372 | 6.0826 | Cytochrome b5 heme-binding domain-containing protein |
| A0A1E3BN35 | 28.199 | 6.0706 | SAP domain-containing protein |
| A0A1E3B613 | 192.69 | 6.0677 | Uncharacterized protein |
| A0A1E3BDX8 | 50.144 | 6.0629 | Uncharacterized protein |
| A0A1E3BMH1 | 51.369 | 6.0613 | 26S protease regulatory subunit 4 |
| A0A1E3BJQ8 | 72.242 | 6.0585 | Uncharacterized protein |
| A0A1E3B1E7 | 81.849 | 6.0558 | Zn(2)-C6 fungal-type domain-containing protein |
| A0A1E3B531 | 29.228 | 6.0534 | Putative urease accessory protein ureG |
| A0A1E3BP96 | 24.585 | 6.0464 | Uncharacterized protein |
| A0A1E3BLU9 | 126.57 | 6.0457 | CBF domain-containing protein |
| A0A1E3BAT2 | 28.987 | 6.0371 | Putative oxidoreductase |
| A0A1E3B2W9 | 72.88 | 6.0341 | Fungal_trans domain-containing protein |
| A0A1E3BAS0 | 52.793 | 6.0329 | 3-hydroxyisobutyryl-CoA hydrolase, mitochondrial |
| A0A1E3B877 | 101.38 | 6.0284 | Uncharacterized protein |
| A0A1E3B6M1 | 54.401 | 6.025 | Adenylosuccinate lyase |
| A0A1E3BJD1 | 122.63 | 6.0236 | Lipase_3 domain-containing protein |
| A0A1E3BKA2 | 85.594 | 6.0221 | Uncharacterized protein |
| A0A1E3BSV5 | 33.467 | 6.0208 | 60S acidic ribosomal protein P0 |
| A0A1E3BTT9 | 152.52 | 6.0197 | Vacuolar import and degradation protein 21 |
| A0A1E3BR60 | 50.158 | 6.0105 | Brix domain-containing protein |
| A0A1E3BSM5 | 72.198 | 6.0081 | Uncharacterized protein |
| A0A1E3BDD3 | 62.174 | 6.0073 | Aspartyl-tRNA synthetase |
| A0A1E3BN17 | 13.743 | 6.0038 | Vacuolar transporter chaperone 1 |
| A0A1E3BLK2 | 56.579 | 5.9989 | Nop domain-containing protein |
| A0A1E3B730 | 70.189 | 5.9956 | Uncharacterized protein |
| A0A1E3B1K7 | 37.682 | 5.9902 | NAD(P)-bd_dom domain-containing protein |
| A0A1E3BEG9 | 114.58 | 5.99 | Zn(2)-C6 fungal-type domain-containing protein |
| A0A1E3BAH2 | 36.532 | 5.9885 | Hydroxymethylbilane synthase |
| A0A1E3B3N8 | 17.984 | 5.9858 | Uncharacterized protein |
| A0A1E3BF49 | 165.58 | 5.985 | Uncharacterized protein |
| A0A1E3BNX3 | 94.629 | 5.9786 | Uncharacterized protein |
| A0A1E3B1G9 | 44.589 | 5.9559 | Ketol-acid reductoisomerase, mitochondrial |
| A0A1E3B8Y6 | 61.021 | 5.9546 | RED_N domain-containing protein |
| A0A1E3B8R6 | 43.518 | 5.9535 | Uncharacterized protein |
| A0A1E3B9U7 | 145.76 | 5.948 | Uncharacterized protein |
| A0A1E3BMC8 | 37.44 | 5.9467 | Uncharacterized protein |
| A0A1E3B318 | 83.443 | 5.9466 | RING-type E3 ubiquitin transferase |
| A0A1E3BP53 | 92.781 | 5.9399 | PHD domain-containing protein |
| A0A1E3BFY1 | 39.055 | 5.9344 | Uncharacterized protein |
| A0A1E3BR37 | 59.86 | 5.931 | Uncharacterized protein |
| A0A1E3BJV8 | 34.142 | 5.9044 | NADH-ubiquinone oxidoreductase 24 kDa subunit |
| A0A1E3BG23 | 35.381 | 5.903 | Uncharacterized protein |
| A0A1E3BG12 | 33.626 | 5.8947 | Mitochondrial DNA replication protein YHM2 |
| A0A1E3BRR4 | 163.84 | 5.8911 | Uncharacterized protein |
| A0A1E3B953 | 44.255 | 5.8691 | Tr-type G domain-containing protein |
| A0A1E3BLN3 | 8.7239 | 5.8562 | Complex I-B15 |
| A0A1E3B450 | 17.101 | 5.8514 | Uncharacterized protein |
| A0A1E3BGW2 | 30.839 | 5.8446 | D-ribose-5-phosphate ketol-isomerase |
| A0A1E3BPS9 | 42.985 | 5.8441 | Uncharacterized protein |
| A0A1E3BAG4 | 76.153 | 5.8411 | Protein-tyrosine-phosphatase |
| A0A1E3B5D1 | 545.47 | 5.8364 | Midasin |
| A0A1E3B6N0 | 19.287 | 5.8339 | Phosphatidylglycerol/phosphatidylinositol transfer protein |
| A0A1E3B1F8 | 73.322 | 5.8335 | GMC_OxRdtase_N domain-containing protein |
| A0A1E3BTH8 | 9.8368 | 5.827 | Uncharacterized protein |
| A0A1E3B0F0 | 14.322 | 5.82 | Hydrophobin |
| A0A1E3B986 | 83.606 | 5.8149 | RFX-type winged-helix domain-containing protein |
| A0A1E3BHW5 | 39.359 | 5.8134 | Uncharacterized protein |
| A0A1E3BVL5 | 58.933 | 5.8126 | Pre-mRNA-splicing factor spp2 |
| A0A1E3BCN3 | 199.54 | 5.8104 | AAA_16 domain-containing protein |
| A0A1B0THQ4 | 42.804 | 5.8096 | Mating type protein MAT1-1 |

Table S9 All the proteins identified in WT-D

| Protein IDs | Mol. weight [kDa] | Score | Descriptions |
| --- | --- | --- | --- |
| A0A1E3BKC2 | 101.71 | 11.281 | C2H2-type domain-containing protein |
| A0A1E3BHC9 | 93.169 | 11.024 | CCHC-type domain-containing protein |
| A0A1E3BGK0 | 129.15 | 6.5526 | Uncharacterized protein |
| A0A1E3BSC0 | 74.078 | 6.1485 | Acetyl-coenzyme A synthetase |
| A0A1E3BSS8 | 37.193 | 11.582 | Serine/threonine-protein phosphatase |
| A0A1E3BL07 | 113.54 | 12.648 | Uncharacterized protein |
| A0A1E3BK49 | 14.734 | 6.0094 | Histone H2A |
| A0A1E3BGV2 | 32.304 | 6.1601 | Uncharacterized protein |
| A0A1E3BBH2 | 17.364 | 11.061 | Uncharacterized protein |
| A0A1E3BN24 | 48.485 | 11.803 | Methionine aminopeptidase 2 |
| A0A1E3BNL0 | 87.773 | 7.2375 | Uncharacterized protein |
| A0A1E3BA79 | 23.872 | 13.616 | Ras-like protein |
| A0A1E3BN13 | 14.419 | 12.318 | Cytochrome b-c1 complex subunit 7 |
| A0A1E3BL93 | 48.545 | 6.036 | Protein phosphatase 2C |
| A0A1E3B856 | 100.09 | 6.1696 | AP-3 complex subunit delta |
| A0A1E3B8X3 | 52.207 | 5.9763 | Uncharacterized protein |
| A0A1E3BN86 | 76.677 | 6.4149 | Zn(2)-C6 fungal-type domain-containing protein |
| A0A1E3B8X9 | 50.292 | 6.1846 | 2-oxoisovalerate dehydrogenase subunit alpha |
| A0A1E3BBT8 | 118.78 | 11.527 | Uncharacterized protein |
| A0A1E3BHX5 | 57.049 | 6.6957 | Cystathionine beta-synthase |
| A0A1E3BDJ2 | 52.017 | 12.41 | Serine hydroxymethyltransferase |
| A0A1E3BH40 | 21.912 | 5.8477 | Alpha-NAC |
| A0A1E3BJQ5 | 120.57 | 6.8363 | Uncharacterized protein |
| A0A1E3BBE0 | 42.292 | 6.2539 | CRAL-TRIO domain-containing protein |
| A0A1E3B330 | 13.112 | 7.4224 | Uncharacterized protein |
| A0A1E3B5W2 | 26.817 | 5.8871 | Uncharacterized protein |
| A0A1E3B860 | 46.227 | 6.1835 | PKS_ER domain-containing protein |
| A0A1E3B9E4 | 36.893 | 10.901 | Epimerase domain-containing protein |
| A0A1E3BPD6 | 30.23 | 6.0991 | Protein hob3 |
| A0A1E3BNM3 | 37.674 | 5.9434 | Uncharacterized protein |
| A0A1E3BDK3 | 36.564 | 12.895 | Uncharacterized protein |
| A0A1E3BC91 | 38.211 | 6.7955 | t-SNARE coiled-coil homology domain-containing protein |
| A0A1E3B384 | 39.906 | 13.003 | Uncharacterized protein |
| A0A1E3B8K7 | 32.062 | 7.2492 | Tudor domain-containing protein |
| A0A1E3BHM0 | 38.138 | 6.3095 | Mitochondrial import receptor subunit tom-40 |
| A0A1E3BFB7 | 50.857 | 7.0363 | Aldedh domain-containing protein |
| A0A1E3BFU0 | 10.246 | 11.94 | ATP synthase subunit e |
| A0A1E3BEE2 | 35.652 | 5.929 | Arginase |
| A0A1E3B9J2 | 35.446 | 12.206 | Uncharacterized protein |
| A0A1E3BRR9 | 36.545 | 11.463 | Uncharacterized protein |
| A0A1E3B7Y1 | 50.845 | 5.8852 | Uncharacterized protein |
| A0A1E3B4A5 | 97.578 | 6.0132 | MINDY_DUB domain-containing protein |
| A0A1E3BKB7 | 31.39 | 12.961 | WSC domain-containing protein |
| A0A1E3BRG3 | 121.27 | 5.8736 | Uncharacterized protein |
| A0A1E3BM73 | 41.276 | 11.844 | Allantoicase |
| A0A1E3BS74 | 53.224 | 6.3407 | Succinate-semialdehyde dehydrogenase |
| A0A1E3BAN2 | 53.092 | 6.7451 | Phosphomevalonate kinase |
| A0A1E3B6B9 | 34.678 | 12.267 | Uricase |
| A0A1E3B876 | 34.704 | 5.8129 | Replication termination factor 2 |
| A0A1E3BRI5 | 50.831 | 6.5812 | Phosphoglycerate dehydrogenase |
| A0A1E3B980 | 24.863 | 6.2073 | Proteasome subunit beta |
| A0A1E3B9W2 | 130.18 | 11.462 | Uncharacterized protein |
| A0A1E3B999 | 16.155 | 6.3927 | 40S ribosomal protein S17 |
| A0A1E3BQY7 | 69.093 | 6.3993 | Uncharacterized protein |
| A0A1E3BDU4 | 108.92 | 16.382 | Plasma membrane ATPase |
| A0A1E3BCR6 | 47.39 | 6.1363 | Short chain specific acyl-CoA dehydrogenase, |
| A0A1E3B563 | 166.92 | 6.0772 | Carboxypeptidase Y receptor |
| A0A1E3B3M0 | 30.442 | 6.696 | Uncharacterized protein |
| A0A1E3BIE6 | 48.737 | 12.226 | Condensation domain-containing protein |
| A0A1E3BCC5 | 30.666 | 11.175 | Prohibitin |
| A0A1E3BA24 | 76.851 | 7.1944 | AMP-binding domain-containing protein |
| A0A1E3BCJ5 | 74.438 | 6.2026 | H/ACA ribonucleoprotein complex non-core subunit NAF1 |
| A0A1E3BJ63 | 6.9342 | 5.9144 | 40S ribosomal protein S30 |
| A0A1E3B1E2 | 52.998 | 12.097 | Aspartyl aminopeptidase |
| A0A1E3BE18 | 22.78 | 18.269 | 40S ribosomal protein S7 |
| A0A1E3BS05 | 89.075 | 6.1452 | Uncharacterized protein |
| A0A1E3BUB0 | 131.35 | 12.519 | Pyruvate carboxylase |
| A0A1E3BI27 | 254.42 | 11.572 | Biotin carboxylase |
| A0A1E3BGV5 | 87.067 | 6.1604 | Double-strand break repair protein |
| A0A1E3BI25 | 83.504 | 12.149 | Uncharacterized protein |
| A0A1E3BLW4 | 86.395 | 11.75 | Uncharacterized protein |
| A0A1E3B8I3 | 40.516 | 6.8506 | Pyruvate dehydrogenase E1 component subunit beta |
| A0A1E3B0U2 | 19.752 | 8.7526 | Altered inheritance of mitochondria protein 41 |
| A0A1E3BJD6 | 62.681 | 21.545 | Ubiquitin carboxyl-terminal hydrolase |
| A0A1E3BAN5 | 90.995 | 6.9735 | Nudix hydrolase domain-containing protein |
| A0A1E3BD65 | 17.096 | 7.314 | Uncharacterized protein |
| A0A1E3BBZ8 | 41.376 | 25.983 | RRM domain-containing protein |
| A0A1E3BM89 | 87.371 | 13.135 | Uncharacterized protein |
| A0A1E3B0U6 | 108.17 | 6.3592 | Importin N-terminal domain-containing protein |
| A0A1E3BSE2 | 27.472 | 23.144 | GST N-terminal domain-containing protein |
| A0A1E3B7T6 | 85.467 | 17.084 | Uncharacterized protein |
| A0A1E3BBR6 | 77.727 | 23.69 | HUN domain-containing protein |
| A0A1E3BSY9 | 73.36 | 17.206 | Uncharacterized protein |
| A0A1E3BRR6 | 29.221 | 11.833 | 40S ribosomal protein S1 |
| A0A1E3BQ79 | 33.362 | 12.588 | Uncharacterized protein |
| A0A1E3B5Y4 | 83.737 | 6.4339 | NUC153 domain-containing protein |
| A0A1E3B268 | 107.49 | 7.0104 | Fe-ADH domain-containing protein |
| A0A1E3BK80 | 54.253 | 8.1187 | BHLH domain-containing protein |
| A0A1E3BHF6 | 48.897 | 7.5365 | Uncharacterized protein |
| A0A1E3BH67 | 19.65 | 19.562 | Uncharacterized protein |
| A0A1E3BA49 | 62.259 | 6.4041 | Uncharacterized protein |
| A0A1E3BE41 | 90.914 | 6.1691 | Non-specific serine/threonine protein kinase |
| A0A1E3BNC0 | 122.49 | 13.535 | Kinesin heavy chain |
| A0A1E3BJL0 | 46.894 | 6.3776 | W2 domain-containing protein |
| A0A1E3B7R5 | 19.768 | 11.296 | Uncharacterized protein |
| A0A1E3B2M3 | 26.754 | 6.2046 | Uncharacterized protein |
| A0A1E3BDY6 | 98.722 | 23.935 | Vacuolar protein sorting-associated protein 35 |
| A0A1E3BD85 | 36.938 | 17.507 | Uncharacterized protein |
| A0A1E3BF41 | 87.241 | 5.9254 | Uncharacterized protein |
| A0A1E3B246 | 46.157 | 11.608 | Citrulline--aspartate ligase |
| A0A1E3BDC8 | 44.693 | 5.8318 | Flap endonuclease 1 |
| A0A1E3B1N6 | 54.606 | 12.916 | Uncharacterized protein |
| A0A1E3BH33 | 122.57 | 6.77 | Uncharacterized protein |
| A0A1E3B2W0 | 33.979 | 11.798 | Alpha-galactosidase |
| A0A1E3BME8 | 26.668 | 13.978 | Uncharacterized protein |
| A0A1E3BC06 | 71.357 | 16.91 | Uncharacterized protein |
| A0A1E3BRN4 | 13.738 | 6.2123 | Autophagy-related protein |
| A0A1E3B761 | 80.445 | 23.368 | Heat shock protein Hsp88 |
| A0A1E3BF24 | 24.731 | 12.11 | Uncharacterized protein |
| A0A1E3BFE8 | 45.273 | 11.934 | cAMP-dependent protein kinase regulatory subunit |
| A0A1E3BPG8 | 53.182 | 13.237 | Uncharacterized protein |
| A0A1E3BN82 | 13.708 | 18.767 | Uncharacterized protein |
| A0A1E3B7K5 | 75.08 | 19.926 | CUE domain-containing protein |
| A0A1E3B3B3 | 57.094 | 12.202 | CCT-beta |
| A0A1E3BIA2 | 9.3475 | 24.624 | Putative cytochrome b5 |
| A0A1E3BML8 | 21.767 | 14.186 | 6,7-dimethyl-8-ribityllumazine synthase |
| A0A1E3BI35 | 35.711 | 12.904 | Endonuclease |
| A0A1E3B5V8 | 37.695 | 7.1518 | PKS_ER domain-containing protein |
| A0A1E3B4W0 | 55.967 | 12.439 | Vacuolar proton pump subunit B |
| A0A1E3BIQ2 | 63.453 | 11.801 | Serine/threonine-protein phosphatase |
| A0A1E3B4J8 | 29.429 | 6.8109 | Protein FYV10 |
| A0A1E3BRE7 | 12.871 | 57.478 | Uncharacterized protein |
| A0A1E3BEK9 | 53.158 | 6.4251 | Uncharacterized protein |
| A0A1E3B9I8 | 37.967 | 6.1356 | PALP domain-containing protein |
| A0A1E3B693 | 67.803 | 11.575 | Uncharacterized protein |
| A0A1E3BQ57 | 48.689 | 27.918 | Ornithine aminotransferase |
| A0A1E3BNX9 | 46.638 | 17.727 | Uncharacterized protein |
| A0A1E3BMK4 | 69.119 | 6.5432 | Carboxypeptidase |
| A0A1E3BP91 | 63.584 | 18.257 | Methylmalonate-semialdehyde dehydrogenase (CoA acylating) |
| A0A1E3B7T1 | 145.69 | 33.448 | Uncharacterized protein |
| A0A1E3B906 | 52.469 | 20.96 | FK506-binding protein |
| A0A1E3BU83 | 55.976 | 17.431 | Uncharacterized protein |
| A0A1E3BDW8 | 39.705 | 11.492 | Peroxin-14 |
| A0A1E3BJW4 | 23.682 | 7.2464 | Lactobacillus shifted protein |
| A0A1E3BJC1 | 81.654 | 7.0648 | Uncharacterized protein |
| A0A1E3BD15 | 56.815 | 14.224 | ENTH domain-containing protein |
| A0A1E3BQG9 | 68.42 | 15.895 | Chromatin modification-related protein |
| A0A1E3BKR8 | 53.456 | 19.579 | NADH-ubiquinone oxidoreductase 49 kDa subunit, |
| A0A1E3BCQ0 | 67.498 | 16.471 | Uncharacterized protein |
| A0A1E3BAS7 | 54.732 | 13.709 | MFAP1 domain-containing protein |
| A0A1E3BDN7 | 101.22 | 6.8148 | Uncharacterized protein |
| A0A1E3BEC4 | 27.838 | 18.79 | Proteasome subunit alpha type |
| A0A1E3BMT1 | 160.53 | 12.829 | Uncharacterized protein |
| A0A1E3BQR7 | 120.94 | 11.895 | Linoleate 8R-lipoxygenase |
| A0A1E3BMG5 | 14.22 | 25.079 | 60S ribosomal protein L22 |
| A0A1E3BF22 | 66.202 | 18.605 | Prolyl-tRNA synthetase |
| A0A1E3B4S0 | 71.571 | 12.082 | Uncharacterized protein |
| A0A1E3BSX0 | 34.079 | 18.654 | ADP/ATP translocase |
| A0A1E3BAD0 | 47.452 | 17.412 | Actin-related protein 3 |
| A0A1E3BK13 | 42.167 | 19.054 | Fe2OG dioxygenase domain-containing protein |
| A0A1E3BQJ4 | 96.946 | 19.276 | Drf_GBD domain-containing protein |
| A0A1E3B9W0 | 51 | 107.07 | Arf-GAP domain-containing protein |
| A0A1E3B8J6 | 37.006 | 27 | D-xylose reductase |
| A0A1E3BUJ4 | 101.76 | 14.781 | Glyco_transf_20 domain-containing protein |
| A0A1E3B3A1 | 77.082 | 26.179 | Amine oxidase |
| A0A1E3BPW7 | 49.178 | 12.027 | Saccharopine dehydrogenase [NADP(+), L-glutamate-forming] |
| A0A1E3BHX9 | 12.198 | 11.221 | ribosomal protein L44 |
| A0A1E3BSE8 | 84.93 | 17.967 | Uncharacterized protein |
| A0A1E3BH64 | 61.624 | 32.05 | 2-methylcitrate dehydratase |
| A0A1E3BJ71 | 18.532 | 22.997 | Putative peroxiredoxin pmp20 |
| A0A1E3BQL6 | 25.764 | 19.121 | t-SNARE coiled-coil homology domain-containing protein |
| A0A1E3BA67 | 40.352 | 17.748 | UDP-glucose 4-epimerase |
| A0A1E3BLY4 | 46.948 | 26.296 | Uncharacterized protein |
| A0A1E3B8W4 | 61.272 | 18.058 | Multifunctional fusion protein |
| A0A1E3BLE2 | 57.826 | 23.882 | BHLH domain-containing protein |
| A0A1E3B8X6 | 126.71 | 55.605 | Uncharacterized protein |
| A0A1E3BLI6 | 48.73 | 37.8 | Adenosylhomocysteinase |
| A0A1E3BAK5 | 38.968 | 37.655 | Guanine nucleotide-binding protein subunit beta |
| A0A1E3BBS9 | 80.818 | 21.586 | TPR_REGION domain-containing protein |
| A0A1E3BDJ6 | 57.188 | 13.037 | Phosphoglycerate mutase (2,3-diphosphoglycerate-independent) |
| A0A1E3B853 | 30.46 | 27.139 | Uncharacterized protein |
| A0A1E3BBE6 | 82.596 | 31.878 | Uncharacterized protein |
| A0A1E3BE14 | 31.151 | 24.449 | Actin-related protein 2/3 complex subunit 5 |
| A0A1E3BTK9 | 50.587 | 6.244 | Uncharacterized protein |
| A0A1E3BMU4 | 6.2392 | 13.218 | 60S ribosomal protein L39 |
| A0A1E3BPF4 | 34.531 | 34.405 | Succinate--CoA ligase [ADP-forming] subunit alpha, mitochondrial |
| A0A1E3BHR2 | 40.003 | 12.434 | Formate dehydrogenase |
| A0A1E3B4V0 | 55.378 | 32.214 | Protein disulfide-isomerase |
| A0A1E3B4V2 | 160.95 | 56.565 | GYF domain-containing protein |
| A0A1E3BLR4 | 14.584 | 16.813 | 60S ribosomal protein L35 |
| A0A1E3BG09 | 98.826 | 24.215 | Aminopeptidase |
| A0A1E3BJ97 | 21.012 | 14.259 | Glucosamine 6-phosphate N-acetyltransferase |
| A0A1E3BBI5 | 52.842 | 12.251 | Cys-Gly metallodipeptidase DUG1 |
| A0A1E3B7L8 | 24.953 | 26.257 | Elongation factor 1-beta |
| A0A1E3BTA1 | 23.143 | 48.735 | Peptidyl-prolyl cis-trans isomerase |
| A0A1E3B3U1 | 72.982 | 5.858 | ANK_REP_REGION domain-containing protein |
| A0A1E3B561 | 50.065 | 24.229 | Acetylornithine transaminase |
| A0A1E3BNG4 | 51.312 | 44.761 | Uncharacterized protein |
| A0A1E3B6J0 | 56.022 | 17.827 | Uncharacterized protein |
| A0A1E3BL96 | 52.622 | 14.067 | Glutaredoxin domain-containing protein |
| A0A1E3BQ62 | 44.153 | 43.616 | Altered inheritance of mitochondria protein 24, |
| A0A1E3BA58 | 22.215 | 11.875 | Cytochrome c oxidase polypeptide 5, mitochondrial |
| A0A1E3BDN4 | 58.084 | 24.931 | Nucleolar protein 58 |
| A0A1E3BKH8 | 49.083 | 13.214 | Uncharacterized protein |
| A0A1E3BT92 | 71.132 | 58.972 | RNA helicase |
| A0A1E3B9A2 | 23.4 | 20.908 | AD domain-containing protein |
| A0A1E3BD91 | 31.233 | 21.406 | D-xylose reductase |
| A0A1E3BIZ8 | 44.557 | 15.451 | Septin spn4 |
| A0A1E3BRH7 | 53.949 | 21.447 | Seryl-tRNA synthetase |
| A0A1E3BP64 | 62.4 | 12.836 | PX domain-containing protein |
| A0A1E3B0I4 | 123.57 | 32.785 | Alpha-mannosidase |
| A0A1E3BRF8 | 47.301 | 90.093 | 2-phosphoglycerate dehydratase |
| A0A1E3B2Y6 | 23.603 | 17.285 | Ras-related protein Rab-11A |
| A0A1E3B472 | 26.849 | 49.873 | Triosephosphate isomerase |
| A0A1E3BM20 | 35.457 | 36.224 | Aldo_ket_red domain-containing protein |
| A0A1E3BSV6 | 25.021 | 6.4176 | Uncharacterized protein |
| A0A1E3BU92 | 39.443 | 21.403 | Sphingolipid long chain base-responsive protein PIL1 |
| A0A1E3BGF0 | 34.812 | 16.726 | RRM domain-containing protein |
| A0A1E3B8V8 | 26.049 | 50.399 | Mago-bind domain-containing protein |
| A0A1E3BQ02 | 39.99 | 20.742 | Peroxidase |
| A0A1E3BNV6 | 23.921 | 15.579 | CFEM domain-containing protein |
| A0A1E3B866 | 50.579 | 39.609 | Eukaryotic translation initiation factor 3 subunit M |
| A0A1E3BMV6 | 30.138 | 26.75 | Phosphomannomutase |
| A0A1E3B5B5 | 72.709 | 15.048 | Carn_acyltransf domain-containing protein |
| A0A1E3B0R2 | 44.736 | 21.076 | RNA helicase |
| A0A1E3B2H7 | 42.58 | 6.1241 | GRF-type domain-containing protein |
| A0A1E3BRL0 | 28.766 | 19.846 | Adenylate kinase |
| A0A1E3BHG8 | 59.183 | 26.729 | Amine oxidase |
| A0A1E3BDW4 | 77.242 | 24.723 | Transketolase |
| A0A1E3B621 | 40.697 | 23.449 | Peptidyl-prolyl cis-trans isomerase D |
| A0A1E3B0G7 | 105.96 | 5.8861 | Uncharacterized protein |
| A0A1E3BGX2 | 71.425 | 11.02 | ATP citrate synthase |
| A0A1E3BBY3 | 10.532 | 6.1997 | Ribosomal protein L37 |
| A0A1E3BF55 | 64.247 | 17.054 | Uncharacterized protein |
| A0A1E3BBX6 | 69.884 | 58.526 | Eukaryotic translation initiation factor 2A |
| A0A1E3B304 | 40.301 | 19.341 | Phospho-2-dehydro-3-deoxyheptonate aldolase |
| A0A1E3BT07 | 32.407 | 21.798 | ATP synthase subunit gamma |
| A0A1E3B4G5 | 27.161 | 13.025 | Ubiquitin carboxyl-terminal hydrolase |
| A0A1E3BBW4 | 16.575 | 22.669 | Uncharacterized protein |
| A0A1E3B2E2 | 13.503 | 17.564 | Small nuclear ribonucleoprotein Sm D1 |
| A0A1E3BLR5 | 96.524 | 22.592 | Uncharacterized protein |
| A0A1E3BFW9 | 88.565 | 23.591 | Protein SIP5 |
| A0A1E3BFR1 | 62.076 | 14.546 | Branchpoint-bridging protein |
| A0A1E3BMJ9 | 25.469 | 13.387 | NADH-ubiquinone oxidoreductase 19.3 kDa subunit |
| A0A1E3BMC0 | 264.44 | 52.139 | THO complex subunit 2 |
| A0A1E3BQN0 | 54.4 | 13.552 | NADH dehydrogenase [ubiquinone] flavoprotein 1 |
| A0A1E3BSY5 | 49.94 | 22.155 | Thioredoxin domain-containing protein |
| A0A1E3BPB9 | 29.346 | 13.298 | Putative phosphatase |
| A0A1E3B844 | 78.416 | 41.562 | Transcription initiation factor IIF subunit alpha |
| A0A1E3B4I1 | 54.138 | 32.682 | Phosphotransferase |
| A0A1E3B0T7 | 49.484 | 24.007 | Uncharacterized protein |
| A0A1E3BF51 | 114.53 | 17.55 | Ubiquitin-activating enzyme E1 1 |
| A0A1E3BN10 | 41.427 | 30.649 | Protein ecm33 |
| A0A1E3BF65 | 60.444 | 18.78 | Importin subunit alpha |
| A0A1E3BHE4 | 27.824 | 11.477 | SNRNP27 domain-containing protein |
| A0A1E3B2W3 | 39.653 | 42.665 | Uncharacterized protein |
| A0A1E3B790 | 28.845 | 18.36 | Uncharacterized protein |
| A0A1E3B8C5 | 6.1223 | 29.293 | SMP domain-containing protein |
| A0A1E3BQH4 | 91.243 | 25.275 | Uncharacterized protein |
| A0A1E3B9M4 | 58.022 | 30.732 | Inositol-3-phosphate synthase |
| A0A1E3BB69 | 57.87 | 30.253 | UTP--glucose-1-phosphate uridylyltransferase |
| A0A1E3BQP5 | 61.135 | 17.921 | Cytochrome b5 heme-binding domain-containing protein |
| A0A1E3BNI0 | 58.833 | 100.24 | Glutamate decarboxylase |
| A0A1E3BBC6 | 34.135 | 29.825 | t-SNARE coiled-coil homology domain-containing protein |
| A0A1E3B9Z4 | 49.054 | 37.223 | Uracil-regulated protein 1 |
| A0A1E3B8Y2 | 24.152 | 40.59 | Uncharacterized protein |
| A0A1E3BDM3 | 33.955 | 87.062 | Uncharacterized protein |
| A0A1E3BNA7 | 19.223 | 27.27 | Protein YOP1 |
| A0A1E3BLY3 | 10.853 | 6.7808 | Ubiquitin-like protein SMT3 |
| A0A1E3BAZ3 | 59.459 | 47.984 | Uncharacterized protein |
| A0A1E3BRL6 | 18.225 | 48.28 | Uncharacterized protein |
| A0A1E3BA21 | 17.625 | 41.938 | Actin-depolymerizing factor 1 |
| A0A1E3BG47 | 66.92 | 25.486 | Heat shock protein SSB1 |
| A0A1E3BS97 | 16.443 | 29.352 | 40S ribosomal protein S12 |
| A0A1E3BP98 | 39.829 | 24.71 | Glutamine synthetase |
| A0A1E3BBP9 | 49.827 | 22.952 | Uncharacterized protein |
| A0A1E3B5Z3 | 46.972 | 19.507 | Uncharacterized protein |
| A0A1E3BN19 | 37.575 | 20.195 | Adenosine kinase |
| A0A1E3BRT9 | 50.479 | 21.491 | Uncharacterized protein |
| A0A1E3B9V5 | 17.728 | 14.329 | 40S ribosomal protein S15 |
| A0A1E3B1R9 | 21.83 | 20.072 | GLTP domain-containing protein |
| A0A1E3BHB9 | 8.8143 | 6.1944 | Small nuclear ribonucleoprotein G |
| A0A1E3B957 | 54.101 | 124.48 | 6-phosphogluconate dehydrogenase, decarboxylating |
| A0A1E3BD42 | 29.357 | 25.306 | 14-3-3 protein |
| A0A1E3BGH5 | 58.176 | 24.227 | Cell division control protein 3 |
| A0A1E3BCK8 | 15.333 | 18.291 | Histone H3 |
| A0A1E3B8U6 | 41.95 | 30.513 | Uncharacterized protein |
| A0A1E3BP75 | 25.1 | 24.587 | Uncharacterized protein |
| A0A1E3B507 | 9.514 | 66.681 | Uncharacterized protein |
| A0A1E3B5W4 | 76.277 | 55.556 | Terpene cyclase/mutase family member |
| A0A1E3B2H6 | 76.039 | 20.418 | Uncharacterized protein |
| A0A1E3BMW9 | 80.935 | 44.572 | Uncharacterized protein |
| A0A1E3B749 | 58.833 | 51.098 | Uncharacterized protein |
| A0A1E3BQS2 | 34.923 | 15 | EVE domain-containing protein |
| A0A1E3BQW4 | 12.867 | 18.108 | Small nuclear ribonucleoprotein Sm D3 |
| A0A1E3BGG7 | 41.043 | 26.056 | Acetyl-CoA acetyltransferase IB |
| A0A1E3BQE7 | 21.271 | 25.685 | Uncharacterized protein OS |
| A0A1E3B7K2 | 34.551 | 25.273 | Autophagy-related protein 27 |
| A0A1E3B7A8 | 65.016 | 12.443 | Uncharacterized protein |
| A0A1E3BAX3 | 15.868 | 42.835 | 40S ribosomal protein S14 |
| A0A1E3BBF8 | 101.8 | 50.14 | Uncharacterized protein |
| A0A1E3BSZ7 | 7.5516 | 11.301 | 60S ribosomal protein L29 |
| A0A1E3BG63 | 53.501 | 54.838 | Uncharacterized protein |
| A0A1E3BBF1 | 13.016 | 25.778 | Uncharacterized protein |
| A0A1E3BRL4 | 39.421 | 32.15 | 26S proteasome regulatory subunit rpn-8 |
| A0A1E3BM80 | 99.525 | 40.603 | Uncharacterized protein |
| A0A1E3BKS0 | 26.525 | 29.334 | ATP synthase subunit 4 |
| A0A1E3BQ07 | 22.975 | 19.874 | Uncharacterized protein |
| A0A1E3BGP1 | 18.586 | 50.699 | Mitochondrial import receptor subunit tom-20 |
| A0A1E3BEK5 | 27.165 | 15.28 | Uncharacterized protein |
| A0A1E3BJ67 | 16.531 | 32.492 | 60S ribosomal protein L |
| A0A1E3BTG7 | 9.2348 | 22.087 | 60S ribosomal protein L38 |
| A0A1E3BQ14 | 106.16 | 84.476 | Aminopeptidase |
| A0A1E3BGU6 | 52.818 | 37.272 | ATP citrate synthase |
| A0A1E3BRV2 | 21.59 | 51.979 | GTP-binding protein rhoA |
| A0A1E3BPR5 | 34.646 | 135.81 | C2H2-type domain-containing protein |
| A0A1E3BH08 | 51.075 | 25.441 | Citrate synthase |
| A0A1E3BN41 | 30.567 | 19.314 | MSP domain-containing protein |
| A0A1E3BRX4 | 59.663 | 89.998 | Uncharacterized protein |
| A0A1E3B1R6 | 47.04 | 53.994 | Uncharacterized protein |
| A0A1E3BP32 | 94.56 | 47.575 | Uncharacterized protein |
| A0A1E3BC17 | 53.621 | 35.385 | Aldedh domain-containing protein |
| A0A1E3BSU6 | 10.298 | 12.685 | Guanine nucleotide-binding protein subunit gamma |
| A0A1E3BNY7 | 51.342 | 54.229 | Uncharacterized protein |
| A0A1E3BRX5 | 30.413 | 56.425 | 14-3-3 protein |
| A0A1E3BSJ1 | 79.32 | 59.29 | Polyadenylate-binding protein |
| A0A1E3BR91 | 19.675 | 10.705 | Uncharacterized protein |
| A0A1E3BF08 | 44.478 | 71.24 | Peptidase_M24 domain-containing protein |
| A0A1E3B735 | 10.478 | 29.168 | MICOS complex subunit MIC10 |
| A0A1E3BAU1 | 27.507 | 81.802 | Uncharacterized protein |
| A0A1E3BFL8 | 65.688 | 12.916 | Tripeptidyl-peptidase sed2 |
| A0A1E3BL22 | 57.469 | 62.693 | Uncharacterized protein |
| A0A1E3B520 | 137.63 | 17.83 | Protein transport protein SEC31 |
| A0A1E3BIL6 | 74.466 | 36.014 | Uncharacterized protein |
| A0A1E3B267 | 140.13 | 43.654 | Uncharacterized protein |
| A0A1E3BIM2 | 35.389 | 59.932 | Transaldolase |
| A0A1E3BPJ4 | 51.7 | 32.599 | Uncharacterized protein |
| A0A1E3BL55 | 25.442 | 69.781 | Uncharacterized protein |
| A0A1E3BCA6 | 123.97 | 58.967 | Isoleucyl-tRNA synthetase |
| A0A1E3BJ86 | 32.019 | 34.082 | Uncharacterized protein |
| A0A1E3BFR0 | 113.26 | 75.334 | Uncharacterized protein |
| A0A1E3B3J2 | 32.247 | 34.858 | Pyridoxal 5-phosphate synthase (glutamine hydrolyzing) |
| A0A1E3BMJ5 | 54.877 | 36.637 | 4-aminobutyrate aminotransferase |
| A0A1E3B2S5 | 86.87 | 105.4 | 5-methyltetrahydropteroyltriglutamate--homocysteine S-methyltransferase |
| A0A1E3BDA1 | 21.405 | 14.783 | Uncharacterized protein |
| A0A1E3BCE9 | 25.138 | 68.875 | Ribosomal_L7Ae domain-containing protein |
| A0A1E3BGL0 | 57.2 | 44.368 | Uncharacterized protein |
| A0A1E3B777 | 42.487 | 41.697 | Uncharacterized protein |
| A0A1E3B1Z6 | 63.949 | 65.885 | Nucleolar protein 58 |
| A0A1E3BM00 | 264.93 | 84.537 | Carrier domain-containing protein |
| A0A1E3BLC6 | 20.022 | 18.789 | Uncharacterized protein |
| A0A1E3BPQ6 | 85.43 | 64.765 | Aconitate hydratase, mitochondrial |
| A0A1E3BGI3 | 135.92 | 78.742 | Phospholipase D |
| A0A1E3BSM0 | 78.251 | 56.987 | Ubiquitin |
| A0A1E3BJC4 | 17.017 | 56.935 | Nascent polypeptide-associated complex subunit beta |
| A0A1E3B6G7 | 17.215 | 114.95 | Putative ribose 5-phosphate isomerase |
| A0A1E3BSV0 | 118.09 | 115.81 | Eukaryotic translation initiation factor 3 subunit A |
| A0A1E3B589 | 36.339 | 58.044 | NmrA domain-containing protein |
| A0A1E3B4U5 | 93.549 | 72.391 | Elongation factor 2 |
| A0A1E3BM63 | 56.818 | 46.998 | Amino_oxidase domain-containing protein |
| A0A1E3BT27 | 118.83 | 66.489 | Eukaryotic translation initiation factor 5B |
| A0A1E3BAB8 | 55.143 | 104.98 | ATP synthase subunit beta |
| A0A1E3B7H4 | 151.06 | 123.01 | RNB domain-containing protein |
| A0A1E3BD66 | 66.079 | 57.008 | Alkaline phosphatase |
| A0A1E3BCR3 | 54.154 | 34.728 | Uncharacterized protein |
| A0A1E3BG22 | 80.034 | 80.494 | Heat shock protein |
| A0A1E3BR33 | 49.95 | 50.243 | Elongation factor 1-alpha |
| A0A1E3B3M4 | 36.51 | 49.131 | RRM domain-containing protein |
| A0A1E3BTS1 | 11.937 | 31.252 | 60S ribosomal protein L36 |
| A0A1E3BN60 | 42.913 | 50.648 | Diphosphomevalonate decarboxylase |
| A0A1E3B9D4 | 55.28 | 77.002 | zf-LYAR domain-containing protein |
| A0A1E3BGF3 | 63.793 | 31.685 | U2 snRNP auxiliary factor large subunit |
| A0A1E3BN38 | 113.44 | 183.98 | LsmAD domain-containing protein |
| A0A1E3BBH4 | 28.351 | 49.672 | RRM domain-containing protein |
| A0A1E3BAV9 | 13.094 | 21.666 | Small nuclear ribonucleoprotein Sm D2 |
| A0A1E3BKS5 | 59.636 | 145.63 | ATP synthase subunit alpha |
| A0A1E3BSZ6 | 17.869 | 38.012 | 60S ribosomal protein L12 |
| A0A1E3BB21 | 62.807 | 80.491 | Uncharacterized protein |
| A0A1E3BKR7 | 97.65 | 94.524 | Eukaryotic translation initiation factor 3 subunit C |
| A0A1E3BRH1 | 16.932 | 70.349 | 60S ribosomal protein L25 |
| A0A1E3BLQ3 | 19.986 | 36.854 | Translationally-controlled tumor protein homolog |
| A0A1E3BK91 | 51.308 | 56.068 | eIF-5a domain-containing protein |
| A0A1E3BSA9 | 16.435 | 128.57 | 40S ribosomal protein S19 |
| A0A1E3B590 | 24.12 | 52.347 | ATP synthase subunit 5, mitochondrial |
| A0A1E3B2I5 | 73.022 | 79.938 | Endoplasmic reticulum chaperone BiP |
| A0A1E3BN75 | 33.179 | 60.255 | D-xylose reductase |
| A0A1E3BIZ3 | 40.364 | 72.609 | 60S ribosomal protein L4-B |
| A0A1E3B6U6 | 43.164 | 81.171 | Uncharacterized protein |
| A0A1E3BSR2 | 12.283 | 197.31 | Uncharacterized protein |
| A0A1E3B544 | 10.896 | 68.961 | Cytochrome c oxidase subunit 6B |
| A0A1E3B7D1 | 24.113 | 49.197 | Ribosomal protein |
| A0A1E3BSN1 | 37.633 | 34.977 | PKS_ER domain-containing protein |
| A0A1E3BJ81 | 58.601 | 69.404 | Fumarate hydratase |
| A0A1E3BU69 | 21.549 | 45.766 | U6 snRNA-associated Sm-like protein LSm6 |
| A0A1E3B004 | 32.474 | 66.462 | Inorganic diphosphatase |
| A0A1E3B193 | 16.655 | 138.84 | Multiprotein-bridging factor 1 |
| A0A1E3BLF3 | 33.89 | 90.764 | Uncharacterized protein |
| A0A1E3BIT5 | 19.318 | 80.22 | ATP synthase subunit d, mitochondrial |
| A0A1E3B3K8 | 27.23 | 25.438 | Uncharacterized protein |
| A0A1E3BCJ0 | 56.642 | 304.57 | Uncharacterized protein |
| A0A1E3BF47 | 47.314 | 67.707 | tRNA-binding domain-containing protein |
| A0A1E3B728 | 34.602 | 7.5277 | 60S ribosomal protein L5 |
| A0A1E3BBR1 | 23.38 | 98.142 | Histone H1 |
| A0A1E3BG33 | 51.747 | 56.288 | Citrate synthase |
| A0A1E3B6Q8 | 153.11 | 207.27 | MIF4G domain-containing protein |
| A0A1E3BN58 | 77.1 | 101.81 | HTH La-type RNA-binding domain-containing protein |
| A0A1E3BG15 | 61.998 | 95.807 | Carboxypeptidase |
| A0A1E3BPC9 | 61.31 | 75.808 | Calnexin |
| A0A1E3BPN7 | 52.99 | 28.939 | Uncharacterized protein |
| A0A1E3BAM6 | 40.366 | 71.453 | Protein disulfide-isomerase tigA |
| A0A1E3BRM2 | 69.362 | 85.22 | Heat shock 70 kDa protein |
| A0A1E3BM22 | 49.684 | 76.542 | Uncharacterized protein |
| A0A1E3BA65 | 33.966 | 97.689 | Succinate dehydrogenase [ubiquinone] iron-sulfur subunit, mitochondrial |
| A0A1E3BCS8 | 14.224 | 72.741 | U6 snRNA-associated Sm-like protein LSm6 |
| A0A1E3B0S1 | 46.023 | 87.071 | Uncharacterized protein |
| A0A1E3B0X1 | 35.721 | 37.846 | Malate dehydrogenase |
| A0A1E3BM19 | 22.187 | 100.68 | Uncharacterized protein |
| A0A1E3B419 | 25.75 | 63.924 | RRM domain-containing protein |
| A0A1E3BPL0 | 18.001 | 32.816 | 40S ribosomal protein S10-B |
| A0A1E3BHN4 | 16.991 | 111.45 | Ribosomal_L28e domain-containing protein |
| A0A1E3BL41 | 61.665 | 81.187 | Heat shock protein 60 |
| A0A1E3BI52 | 34.425 | 99.642 | Malate dehydrogenase |
| A0A1E3BEX0 | 47.187 | 215.19 | RRM domain-containing protein |
| A0A1E3BCG8 | 11.356 | 26.747 | Histone H4 |
| A0A1E3B1Z29 | 92.62 | 75.586 | Aldo_ket_red domain-containing protein |
| A0A1E3BN70 | 49.558 | 112.71 | Glutamate dehydrogenase |
| A0A1E3BD08 | 18.705 | 47.731 | Peptidyl-prolyl cis-trans isomerase |
| A0A1E3BBH7 | 14.076 | 6.8884 | Histone H2A |
| A0A1E3B7G9 | 41.325 | 157.31 | Uncharacterized protein |
| A0A1E3B0L0 | 53.888 | 134.77 | Aldehyde dehydrogenase |
| A0A1E3BJ79 | 67.803 | 118.93 | WH1 domain-containing protein |
| A0A1E3B759 | 9.9194 | 82.892 | 40S ribosomal protein S25 |
| A0A1E3B6H6 | 59.708 | 18.773 | RNA helicase |
| A0A1E3BEB7 | 12.474 | 58.463 | Non-histone chromosomal protein 6 |
| A0A1E3BNV8 | 36.886 | 117.03 | D-xylose reductase |
| A0A1E3B7Z0 | 42.498 | 79.633 | Mannitol-1-phosphate 5-dehydrogenase |
| A0A1E3BFM7 | 7.2633 | 66.691 | Coiled-coil-containing protein 72 |
| A0A1E3B8L2 | 74.331 | 58.438 | DUF2433 domain-containing protein |
| A0A1E3BIV9 | 14.762 | 103.06 | Uncharacterized protein |
| A0A1E3B5W0 | 24.385 | 151.69 | Uncharacterized protein |
| A0A1E3BBK3 | 14.955 | 59.182 | Histone H2B |
| A0A1E3BRR2 | 53.738 | 17.786 | Uncharacterized protein |
| A0A1E3B4X0 | 12.13 | 125.38 | Cytochrome c |
| A0A1E3BLL0 | 40.389 | 75.829 | Glyceraldehyde-3-phosphate dehydrogenase |
| A0A1E3BMD2 | 36.161 | 311.02 | Uncharacterized protein |
| A0A1E3BEM7 | 32.867 | 323.31 | HABP4_PAI-RBP1 domain-containing protein |

Table S10 All differentially expressedgenes in △*Achog1* vs wild type

| GeneID | Gene_Length | | Log_2_FC  (△*Achog1*/WT) | Description |  |
| --- | --- | --- | --- | --- | --- |
| gene-SI65_04725 | 207 | 6.255 | | hypothetical protein |  |
| gene-SI65_06822 | 162 | 5.250 | | hypothetical protein |  |
| gene-SI65_05992 | 1122 | 5.165 | | Reverse transcriptase (RNA-dependent DNA polymerase) |  |
| gene-SI65_08155 | 780 | 4.962 | | hypothetical protein |  |
| gene-SI65_09912 | 468 | 4.850 | | hypothetical protein |  |
| novel.294 | 963 | 4.491 | | unknown |  |
| novel.522 | 1030 | 4.147 | | unknown |  |
| gene-SI65_01919 | 1617 | 3.795 | | Low-affinity glucose transporter HXT3 |  |
| gene-SI65_05766 | 888 | 3.722 | | NADH-cytochrome b5 reductase 2 |  |
| gene-SI65_09954 | 1725 | 3.595 | | Phosphate-repressible phosphate permease pho-4 |  |
| gene-SI65_05026 | 636 | 3.428 | | hypothetical protein |  |
| gene-SI65_04726 | 690 | 3.405 | | hypothetical protein |  |
| gene-SI65_06451 | 426 | 3.341 | | hypothetical protein |  |
| gene-SI65_04687 | 675 | 3.297 | | hypothetical protein |  |
| gene-SI65_05015 | 444 | 3.250 | | hypothetical protein |  |
| gene-SI65_00200 | 555 | 3.234 | | Cell wall protein phiA |  |
| gene-SI65_03401 | 6510 | 3.209 | | TPR and ankyrin repeat-containing protein 1 |  |
| gene-SI65_03378 | 807 | 3.065 | | FAD dependent oxidoreductase |  |
| gene-SI65_02819 | 210 | 3.056 | | hypothetical protein |  |
| gene-SI65_08227 | 642 | 3.050 | | hypothetical protein |  |
| gene-SI65_07943 | 1482 | 2.997 | | Pyoverdine/dityrosine biosynthesis protein |  |
| gene-SI65_07300 | 975 | 2.909 | | putative pectate lyase A |  |
| gene-SI65_10330 | 1824 | 2.898 | | Fungal specific transcription factor domain |  |
| gene-SI65_04337 | 471 | 2.876 | | Antigenic thaumatin-like protein ARB_01932 |  |
| gene-SI65_05459 | 663 | 2.854 | | Acetyltransferase (GNAT) family |  |
| gene-SI65_02169 | 306 | 2.8451 | | hypothetical protein |  |
| gene-SI65_07161 | 708 | 2.832 | | UPF0619 GPI-anchored membrane protein AFUA_3G00880 |  |
| novel.339 | 1087 | 2.826 | | unknown |  |
| novel.418 | 894 | 2.817 | | unknown |  |
| gene-SI65_01457 | 1287 | 2.783 | | Protein of unknown function (DUF4243) |  |
| novel.367 | 1267 | 2.728 | | unknown |  |
| gene-SI65_09028 | 363 | 2.717 | | hypothetical protein |  |
| gene-SI65_07728 | 1848 | 2.714 | | Dehydrogenase patE |  |
| gene-SI65_07298 | 4026 | 2.713 | | Transposon Ty3-I Gag-Pol polyprotein; AltName |  |
| novel.501 | 3137 | 2.706 | | unknown |  |
| gene-SI65_06766 | 189 | 2.664 | | hypothetical protein |  |
| novel.1 | 1395 | 2.658 | | unknown |  |
| gene-SI65_03164 | 1482 | 2.648 | | protein sll1024 |  |
| gene-SI65_05458 | 417 | 2.642 | | hypothetical protein |  |
| gene-SI65_08716 | 1128 | 2.633 | | Isopullulanase |  |
| gene-SI65_09909 | 660 | 2.6208 | | Cutinase 3 |  |
| gene-SI65_02720 | 216 | 2.612 | | hypothetical protein |  |
| novel.173 | 980 | 2.602 | | unknown |  |
| gene-SI65_06121 | 738 | 2.590 | | hypothetical protein |  |
| gene-SI65_03577 | 288 | 2.557 | | hypothetical protein |  |
| gene-SI65_04116 | 1398 | 2.545 | | Uncharacterized protein AFUA_6G02800 |  |
| gene-SI65_04398 | 951 | 2.530 | | ugar transporter STL1 |  |
| gene-SI65_07944 | 1809 | 2.494 | | Laccase-2 |  |
| gene-SI65_04538 | 345 | 2.491 | | hypothetical protein |  |
| gene-SI65_02967 | 1266 | 2.467 | | hypothetical protein |  |
| novel.218 | 532 | 2.464 | | unknown |  |
| gene-SI65_04663 | 390 | 2.443 | | Synembryn-like protein C3E7.04c |  |
| novel.343 | 860 | 2.422 | | unknown |  |
| gene-SI65_04044 | 2073 | 2.404 | | FAD-binding monooxygenase ausC |  |
| gene-SI65_08957 | 471 | 2.390 | | hypothetical protein |  |
| gene-SI65_00869 | 1707 | 2.386 | | Probable beta-glucosidase M |  |
| gene-SI65_09298 | 846 | 2.379 | | Phosphotransferase enzyme family |  |
| gene-SI65_00935 | 459 | 2.359 | | Uracil phosphoribosyltransferase |  |
| gene-SI65_09950 | 2451 | 2.357 | | hypothetical proteinPF00884:Sulfatase |  |
| gene-SI65_03283 | 576 | 2.330 | | Uncharacterized secreted protein ARB_07637 |  |
| gene-SI65_05927 | 396 | 2.326 | | hypothetical protein |  |
| novel.236 | 2414 | 2.322 | | unknown |  |
| gene-SI65_05465 | 483 | 2.317 | | hypothetical protein |  |
| novel.71 | 1049 | 2.316 | | unknown |  |
| gene-SI65_10267 | 1608 | 2.309 | | Uncharacterized transporter YIL166C |  |
| gene-SI65_08554 | 456 | 2.308 | | hypothetical protein |  |
| gene-SI65_04404 | 471 | 2.287 | | Ribonuclease III domain |  |
| novel.403 | 2528 | 2.285 | | Glutathione-dependent formaldehyde-activating enzyme |  |
| gene-SI65_04151 | 549 | 2.281 | | GTP cyclohydrolase 1 |  |
| gene-SI65_03326 | 1506 | 2.275 | | Sphingolipid C9-methyltransferase 1 |  |
| gene-SI65_05309 | 741 | 2.268 | | Protein of unknown function (DUF2990) |  |
| gene-SI65_09913 | 1500 | 2.267 | | Trichodiene oxygenase |  |
| gene-SI65_01490 | 1779 | 2.265 | | Pyranose dehydrogenase 2 |  |
| gene-SI65_06936 | 2328 | 2.250 | | FAD-dependent monooxygenase asqG |  |
| gene-SI65_09907 | 1356 | 2.250 | | Non-specific phospholipase C2 |  |
| gene-SI65_00405 | 1680 | 2.239 | | hypothetical protein |  |
| gene-SI65_02864 | 915 | 2.239 | | hypothetical protein |  |
| gene-SI65_07110 | 324 | 2.238 | | hypothetical protein |  |
| gene-SI65_08176 | 918 | 2.229 | | putative voltage-gated potassium channel subunit beta |  |
| gene-SI65_01466 | 1455 | 2.225 | | Uncharacterized MFS-type transporter C947.06c |  |
| gene-SI65_10099 | 414 | 2.222 | | hypothetical protein |  |
| gene-SI65_02762 | 873 | 2.221 | | hypothetical protein |  |
| gene-SI65_02372 | 540 | 2.217 | | hypothetical protein |  |
| gene-SI65_06915 | 2808 | 2.216 | | Uncharacterized protein YetA |  |
| gene-SI65_07160 | 1569 | 2.215 | | hypothetical protein |  |
| gene-SI65_00732 | 195 | 2.209 | | hypothetical protein |  |
| gene-SI65_08992 | 1485 | 2.205 | | Alpha-1,2-mannosyltransferase MNN24 |  |
| gene-SI65_05954 | 348 | 2.204 | | hypothetical protein |  |
| gene-SI65_09251 | 321 | 2.188 | | Cyanovirin-N |  |
| gene-SI65_04489 | 186 | 2.169 | | hypothetical protein |  |
| gene-SI65_05065 | 999 | 2.169 | | Lysophospholipase |  |
| gene-SI65_10240 | 1116 | 2.167 | | hypothetical protein |  |
| gene-SI65_07036 | 531 | 2.160 | | Ankyrin repeats (3 copies) |  |
| gene-SI65_09914 | 153 | 2.155 | | hypothetical protein |  |
| gene-SI65_04354 | 711 | 2.154 | | hypothetical protein |  |
| gene-SI65_07799 | 660 | 2.154 | | Eukaryotic initiation factor 4E |  |
| gene-SI65_09816 | 1701 | 2.149 | | hypothetical protein |  |
| gene-SI65_05923 | 1251 | 2.148 | | Choline monooxygenase, chloroplastic |  |
| gene-SI65_02453 | 990 | 2.145 | | SRSF protein kinase 3 |  |
| gene-SI65_02763 | 501 | 2.13 | | Cupin domain |  |
| gene-SI65_07348 | 408 | 2.120 | | hypothetical protein |  |
| gene-SI65_07267 | 393 | 2.118 | | hypothetical protein |  |
| novel.453 | 1612 | 2.118 | | Ribonuclease mitogillin |  |
| gene-SI65_01085 | 996 | 2.117 | | hypothetical protein |  |
| gene-SI65_05955 | 588 | 2.113 | | hypothetical protein |  |
| gene-SI65_05244 | 666 | 2.110 | | Probable endo-1,4-beta-xylanase B |  |
| novel.330 | 1238 | 2.097 | | unknown |  |
| gene-SI65_08844 | 900 | 2.089 | | 5'-nucleotidase SurE |  |
| gene-SI65_00936 | 1557 | 2.031 | | hypothetical protein |  |
| gene-SI65_09245 | 615 | 2.023 | | hypothetical protein |  |
| gene-SI65_08442 | 642 | 2.011 | | hypothetical protein |  |
| gene-SI65_01467 | 924 | 1.983 | | Succinate-semialdehyde dehydrogenase [NADP(+)] |  |
| novel.151 | 339 | 1.975 | | unknown |  |
| novel.116 | 1635 | 1.975 | | unknown |  |
| gene-SI65_04314 | 882 | 1.968 | | Lipase |  |
| gene-SI65_03307 | 804 | 1.968 | | hypothetical protein |  |
| gene-SI65_00113 | 1083 | 1.950 | | Endo-1,4-beta-xylanase D |  |
| novel.285 | 3589 | 1.942 | | unknown |  |
| gene-SI65_09644 | 1059 | 1.938 | | Dioxygenase |  |
| gene-SI65_00457 | 1071 | 1.920 | | Low specificity L-threonine aldolase |  |
| gene-SI65_08170 | 1635 | 1.910 | | Uncharacterized MFS-type transporter |  |
| gene-SI65_08928 | 804 | 1.909 | | hypothetical protein |  |
| gene-SI65_08630 | 1569 | 1.907 | | Efflux pump FUB11 |  |
| gene-SI65_10096 | 891 | 1.906 | | Mono- and diacylglycerol lipase |  |
| gene-SI65_04400 | 1758 | 1.901 | | FGGY family of carbohydrate kinases |  |
| gene-SI65_03369 | 744 | 1.898 | | hypothetical protein |  |
| novel.499 | 441 | 1.895 | | unknown |  |
| gene-SI65_01752 | 4341 | 1.894 | | Ran-binding protein 10 |  |
| gene-SI65_10239 | 774 | 1.891 | | Tryprostatin B 6-hydroxylase |  |
| gene-SI65_03926 | 336 | 1.885 | | Putative oxalocrotonate tautomerase enzyme |  |
| gene-SI65_02694 | 435 | 1.879 | | hypothetical protein |  |
| gene-SI65_10153 | 2853 | 1.879 | | hypothetical protein |  |
| gene-SI65_00685 | 1977 | 1.878 | | Fungal specific transcription factor domain |  |
| gene-SI65_00499 | 663 | 1.873 | | Chromo (CHRromatin Organisation MOdifier) domain |  |
| gene-SI65_03321 | 5739 | 1.865 | | NFX1-type zinc finger-containing protein 1 |  |
| gene-SI65_07171 | 1500 | 1.861 | | CorA-like Mg2+ transporter protein |  |
| gene-SI65_03322 | 513 | 1.854 | | hypothetical protein |  |
| novel.163 | 587 | 1.851 | | unknown |  |
| gene-SI65_08264 | 2598 | 1.847 | | Probable alpha/beta-glucosidase agdC |  |
| gene-SI65_10129 | 417 | 1.843 | | hypothetical protein |  |
| gene-SI65_06032 | 678 | 1.835 | | GTP cyclohydrolase |  |
| novel.292 | 1463 | 1.830 | | unknown |  |
| gene-SI65_06108 | 507 | 1.825 | | hypothetical protein |  |
| novel.369 | 1112 | 1.823 | | unknown |  |
| gene-SI65_09397 | 1350 | 1.811 | | hypothetical protein |  |
| gene-SI65_05980 | 9426 | 1.810 | | Protein of unknown function (DUF3638) |  |
| gene-SI65_04328 | 972 | 1.803 | | putative alpha-L-arabinofuranosidase axhA-1 |  |
| novel.274 | 715 | 1.797 | | unknown |  |
| gene-SI65_02863 | 1353 | 1.793 | | Uncharacterized protein conserved in bacteria (DUF2252) |  |
| gene-SI65_07458 | 294 | 1.792 | | hypothetical protein |  |
| gene-SI65_02098 | 654 | 1.789 | | Small nuclear ribonucleoprotein-associated protein B |  |
| gene-SI65_02708 | 825 | 1.789 | | hypothetical protein |  |
| gene-SI65_09362 | 1305 | 1.787 | | hypothetical protein |  |
| gene-SI65_08847 | 1179 | 1.785 | | Endoglucanase-4 |  |
| gene-SI65_07035 | 1422 | 1.783 | | Ankyrin repeats (many copies) |  |
| gene-SI65_01103 | 396 | 1.783 | | hypothetical protein |  |
| gene-SI65_03332 | 606 | 1.778 | | hypothetical protein |  |
| gene-SI65_08631 | 1986 | 1.772 | | Fungal specific transcription factor domain |  |
| gene-SI65_02113 | 633 | 1.771 | | Peptidyl-prolyl cis-trans isomerase B |  |
| gene-SI65_07483 | 783 | 1.768 | | Acetate permease A |  |
| gene-SI65_00163 | 1038 | 1.762 | | Protein of unknown function (DUF3712) |  |
| gene-SI65_06593 | 1143 | 1.754 | | Pectin lyase A |  |
| gene-SI65_07725 | 1314 | 1.752 | | SMP-30/Gluconolaconase/LRE-like region |  |
| novel.280 | 251 | 1.752 | | unknown |  |
| gene-SI65_09937 | 1773 | 1.751 | | Protein of unknown function (DUF3176) |  |
| gene-SI65_05069 | 804 | 1.750 | | BTB/POZ domain |  |
| gene-SI65_04623 | 1104 | 1.750 | | hypothetical protein |  |
| gene-SI65_10264 | 1332 | 1.743 | | Putative lysine N-acyltransferase C17G9.06c |  |
| gene-SI65_06114 | 1485 | 1.742 | | Uncharacterized FAD-linked oxidoreductase ARB_02372 |  |
| gene-SI65_03890 | 396 | 1.737 | | Protein of unknown function (DUF3759) |  |
| gene-SI65_04298 | 1332 | 1.737 | | Acyl-CoA desaturase |  |
| gene-SI65_04932 | 1347 | 1.733 | | Zinc-type alcohol dehydrogenase-like protein |  |
| gene-SI65_10100 | 354 | 1.729 | | hypothetical protein |  |
| gene-SI65_09758 | 480 | 1.726 | | hypothetical protein |  |
| novel.201 | 1091 | 1.725 | | unknown |  |
| gene-SI65_08574 | 699 | 1.720 | | ncharacterized short-chain type dehydrogenase/reductase |  |
| gene-SI65_06025 | 486 | 1.720 | | hypothetical protein |  |
| novel.393 | 2158 | 1.717 | | unknown |  |
| gene-SI65_10136 | 699 | 1.709 | | 3-beta-hydroxysteroid-Delta(8),Delta(7)-isomerase |  |
| gene-SI65_07454 | 162 | 1.708 | | Antifungal peptide |  |
| gene-SI65_02917 | 864 | 1.706 | | Glycosyl transferase family 90 |  |
| gene-SI65_03340 | 498 | 1.704 | | Copia protein |  |
| gene-SI65_06247 | 609 | 1.704 | | Fungal specific transcription factor domain |  |
| gene-SI65_07811 | 588 | 1.700 | | hypothetical protein |  |
| gene-SI65_09964 | 873 | 1.691 | | Protein of unknown function (DUF3632) |  |
| gene-SI65_05278 | 1983 | 1.689 | | Uncharacterized MFS-type transporter C1399.02 |  |
| gene-SI65_01781 | 528 | 1.688 | | 60S ribosomal protein L11 |  |
| gene-SI65_07773 | 684 | 1.684 | | hypothetical protein |  |
| gene-SI65_07118 | 531 | 1.682 | | hypothetical protein |  |
| gene-SI65_02412 | 1785 | 1.673 | | L-sorbose 1-dehydrogenase |  |
| gene-SI65_04727 | 1053 | 1.672 | | Alcohol dehydrogenase |  |
| gene-SI65_04958 | 1020 | 1.671 | | Monoamine oxidase N |  |
| novel.191 | 812 | 1.661 | | unknown |  |
| gene-SI65_02451 | 594 | 1.659 | | hypothetical protein |  |
| gene-SI65_09149 | 1464 | 1.658 | | UNC93-like protein C922.05c |  |
| gene-SI65_07724 | 1635 | 1.650 | | Efflux pump patC |  |
| gene-SI65_08253 | 2229 | 1.647 | | ATP-binding cassette sub-family F member 3 |  |
| novel.232 | 658 | 1.647 | | unknown |  |
| gene-SI65_04327 | 984 | 1.645 | | Probable endo-1,4-beta-xylanase C |  |
| novel.279 | 889 | 1.644 | | unknown |  |
| gene-SI65_00456 | 696 | 1.641 | | hypothetical protein |  |
| gene-SI65_09295 | 519 | 1.640 | | Hydrophobic surface binding protein A |  |
| gene-SI65_04316 | 963 | 1.638 | | hypothetical protein |  |
| gene-SI65_06245 | 693 | 1.629 | | Putative acetyltransferase |  |
| novel.115 | 1764 | 1.628 | | unknown |  |
| novel.33 | 1219 | 1.622 | | unknown |  |
| gene-SI65_02187 | 2373 | 1.620 | | Sexual differentiation process protein isp4 |  |
| gene-SI65_09330 | 1374 | 1.619 | | Probable pectin lyase F |  |
| gene-SI65_00028 | 315 | 1.616 | | hypothetical protein |  |
| novel.61 | 527 | 1.615 | | unknown |  |
| gene-SI65_03050 | 1620 | 1.615 | | DDE superfamily |  |
| gene-SI65_00922 | 501 | 1.606 | | SnoaL-like polyketide cyclase |  |
| gene-SI65_09407 | 519 | 1.603 | | hypothetical protein |  |
| gene-SI65_00180 | 1485 | 1.603 | | Glucoamylase |  |
| gene-SI65_08110 | 1290 | 1.602 | | Amino-acid acetyltransferase |  |
| gene-SI65_08729 | 564 | 1.600 | | Signal peptidase complex catalytic subunit SEC11 |  |
| gene-SI65_05765 | 2085 | 1.600 | | C6 finger domain transcription factor nscR |  |
| gene-SI65_07470 | 3213 | 1.598 | | Acid trehalase |  |
| gene-SI65_07708 | 348 | 1.597 | | Domain of unknown function (DUF718) |  |
| gene-SI65_09745 | 960 | 1.591 | | Probable arabinan endo-1,5-alpha-L-arabinosidase A |  |
| gene-SI65_08325 | 606 | 1.591 | | hypothetical protein |  |
| novel.175 | 664 | 1.587 | | unknown |  |
| novel.238 | 4966 | 1.578 | | unknown |  |
| gene-SI65_01471 | 1875 | 1.568 | | Beta-1,2-xylosyltransferase 1 |  |
| gene-SI65_08275 | 843 | 1.566 | | Acyl-protein thioesterase 1 |  |
| gene-SI65_02166 | 1482 | 1.566 | | FAD-linked oxidoreductase azaL |  |
| gene-SI65_00210 | 2094 | 1.565 | | hypothetical protein |  |
| novel.47 | 594 | 1.559 | | unknown |  |
| novel.397 | 1085 | 1.554 | | unknown |  |
| gene-SI65_00041 | 801 | 1.553 | | Putative sterigmatocystin biosynthesis peroxidase stcC |  |
| gene-SI65_00209 | 2160 | 1.550 | | Kelch repeat-containing protein ARB_01230 |  |
| gene-SI65_03334 | 639 | 1.548 | | Lipopolysaccharide kinase (Kdo/WaaP) family |  |
| gene-SI65_01036 | 1383 | 1.540 | | 3-beta hydroxysteroid dehydrogenase/isomerase family |  |
| gene-SI65_03314 | 600 | 1.531 | | hypothetical protein |  |
| novel.467 | 1689 | 1.530 | | O-methyltransferase domain |  |
| gene-SI65_07165 | 1113 | 1.524 | | hypothetical protein |  |
| gene-SI65_10274 | 1236 | 1.521 | | Quinate repressor protein |  |
| novel.155 | 2401 | 1.518 | | unknown |  |
| gene-SI65_02421 | 519 | 1.517 | | hypothetical protein |  |
| gene-SI65_07121 | 243 | 1.514 | | hypothetical protein |  |
| gene-SI65_04701 | 1758 | 1.508 | | Thiamine pyrophosphate enzyme, N-terminal TPP binding domain |  |
| gene-SI65_04344 | 999 | 1.507 | | Putative succinate-semialdehyde dehydrogenase C1002.12c [NADP(+)] |  |
| gene-SI65_00923 | 549 | 1.505 | | Bifunctional transcriptional activator/DNA repair enzyme Ada |  |
| gene-SI65_03921 | 1206 | 1.504 | | Acyl-CoA dehydrogenase, C-terminal domain |  |
| gene-SI65_08988 | 528 | 1.502 | | Lipopolysaccharide kinase (Kdo/WaaP) family |  |
| gene-SI65_09511 | 891 | 1.501 | | Methyltransferase domain |  |
| gene-SI65_08189 | 555 | 1.500 | | hypothetical protein |  |
| gene-SI65_03289 | 1398 | 1.496 | | Cytochrome b2 |  |
| novel.65 | 1320 | 1.493 | | unknown |  |
| gene-SI65_00346 | 339 | 1.488 | | hypothetical protein |  |
| gene-SI65_09820 | 795 | 1.484 | | Probable endo-beta-1,4-glucanase D |  |
| gene-SI65_10285 | 765 | 1.483 | | hypothetical protein |  |
| gene-SI65_08418 | 963 | 1.478 | | hypothetical protein |  |
| gene-SI65_10137 | 1386 | 1.478 | | 6-hydroxynicotinate 3-monooxygenase |  |
| novel.23 | 2615 | 1.475 | | unknown |  |
| gene-SI65_06017 | 1809 | 1.474 | | Est1 DNA/RNA binding domain |  |
| gene-SI65_09494 | 933 | 1.465 | | Nitrogen metabolite repression protein nmr |  |
| gene-SI65_07245 | 855 | 1.462 | | Uncharacterized oxidoreductase C162.03 |  |
| novel.275 | 2211 | 1.460 | | BTB/POZ domain |  |
| gene-SI65_07283 | 1632 | 1.456 | | Cycloheximide resistance protein |  |
| novel.264 | 2147 | 1.455 | | unknown |  |
| novel.359 | 886 | 1.452 | | unknown |  |
| novel.157 | 1752 | 1.449 | | unknown |  |
| gene-SI65_08587 | 423 | 1.448 | | hypothetical protein |  |
| gene-SI65_03984 | 1248 | 1.445 | | hypothetical protein |  |
| gene-SI65_04237 | 216 | 1.442 | | hypothetical protein |  |
| novel.143 | 1044 | 1.440 | | unknown |  |
| gene-SI65_01527 | 984 | 1.437 | | Alcohol dehydrogenase 3 |  |
| novel.146 | 1695 | 1.433 | | unknown |  |
| gene-SI65_07726 | 759 | 1.431 | | C-factor |  |
| gene-SI65_05969 | 987 | 1.429 | | hypothetical protein |  |
| gene-SI65_00701 | 2214 | 1.425 | | FAD-dependent monooxygenase asqG |  |
| gene-SI65_08162 | 288 | 1.422 | | hypothetical protein |  |
| gene-SI65_01089 | 1056 | 1.422 | | Full=Arabinogalactan endo-beta-1,4-galactanase A |  |
| novel.321 | 1178 | 1.417 | | unknown |  |
| gene-SI65_01841 | 483 | 1.409 | | 40S ribosomal protein S11-B |  |
| gene-SI65_03078 | 714 | 1.407 | | Ankyrin repeats |  |
| gene-SI65_04033 | 849 | 1.406 | | hypothetical protein |  |
| gene-SI65_09916 | 1104 | 1.406 | | hypothetical protein |  |
| gene-SI65_09072 | 474 | 1.406 | | Dihydrolipoyllysine-residue succinyltransferase component of 2-oxoglutarate dehydrogenase complex |  |
| gene-SI65_00184 | 1470 | 1.404 | | hypothetical protein |  |
| gene-SI65_03053 | 837 | 1.401 | | DDE superfamily endonuclease |  |
| gene-SI65_03656 | 1794 | 1.401 | | Caspase domain |  |
| gene-SI65_10280 | 444 | 1.397 | | Uncharacterized protein |  |
| gene-SI65_05040 | 1692 | 1.397 | | hypothetical protein |  |
| gene-SI65_05075 | 606 | 1.394 | | hypothetical protein |  |
| gene-SI65_07289 | 606 | 1.391 | | Phosphatidylethanolamine-binding protein |  |
| novel.283 | 6089 | 1.391 | | RNase H |  |
| gene-SI65_07179 | 630 | 1.390 | | hypothetical protein |  |
| gene-SI65_00565 | 243 | 1.390 | | hypothetical protein |  |
| gene-SI65_00019 | 384 | 1.390 | | Drug resistance protein |  |
| gene-SI65_02733 | 1089 | 1.385 | | Fungal specific transcription factor domain |  |
| gene-SI65_05972 | 2877 | 1.384 | | Alpha-glucosidase |  |
| gene-SI65_06461 | 525 | 1.380 | | hypothetical protein |  |
| gene-SI65_08492 | 1629 | 1.379 | | Isoflavone 7-O-glucosyltransferase 1 |  |
| novel.117 | 1298 | 1.375 | | unknown |  |
| gene-SI65_07643 | 570 | 1.369 | | hypothetical protein |  |
| novel.502 | 1026 | 1.363 | | unknown |  |
| novel.376 | 227 | 1.359 | | unknown |  |
| gene-SI65_01291 | 423 | 1.356 | | hypothetical protein |  |
| gene-SI65_02754 | 279 | 1.352 | | Domain of unknown function (DUF1857) |  |
| gene-SI65_08410 | 1623 | 1.351 | | Uncharacterized MFS-type transporter |  |
| gene-SI65_07293 | 771 | 1.348 | | Copper fist DNA binding domain |  |
| gene-SI65_08163 | 705 | 1.348 | | Thiopurine S-methyltransferase (TPMT) |  |
| gene-SI65_00899 | 1332 | 1.345 | | hypothetical protein |  |
| gene-SI65_06038 | 516 | 1.340 | | hypothetical protein |  |
| gene-SI65_09131 | 675 | 1.339 | | Tryptophan synthase |  |
| gene-SI65_07023 | 1581 | 1.338 | | putative feruloyl esterase B-2 |  |
| gene-SI65_09435 | 2178 | 1.337 | | hypothetical protein - |  |
| gene-SI65_02396 | 1275 | 1.336 | | Serine/threonine-protein kinase SRPK |  |
| gene-SI65_04566 | 1281 | 1.336 | | putative aspergillopepsin A-like aspartic endopeptidase |  |
| gene-SI65_07995 | 987 | 1.335 | | Probable inactive dehydrogenase EasA |  |
| gene-SI65_08511 | 474 | 1.334 | | Allergen Asp f 15 |  |
| gene-SI65_05514 | 879 | 1.333 | | hypothetical protein |  |
| gene-SI65_09304 | 1149 | 1.330 | | hypothetical protein |  |
| novel.462 | 3224 | 1.330 | | unknown |  |
| gene-SI65_04060 | 498 | 1.325 | | Bifunctional solanapyrone synthase; |  |
| gene-SI65_01367 | 1095 | 1.325 | | D-alanine--D-alanine ligase |  |
| gene-SI65_04402 | 939 | 1.319 | | L-pipecolate oxidase |  |
| gene-SI65_06931 | 1128 | 1.318 | | 1-alkyl-2-acetylglycerophosphocholine esterase |  |
| gene-SI65_08228 | 1008 | 1.318 | | SET domain-containing protein 5 |  |
| gene-SI65_08934 | 1350 | 1.317 | | Aspartic protease pep1 |  |
| gene-SI65_02571 | 1788 | 1.315 | | hypothetical protein |  |
| novel.427 | 1687 | 1.314 | | DDE superfamily endonuclease |  |
| gene-SI65_06026 | 330 | 1.313 | | hypothetical protein |  |
| gene-SI65_07288 | 1092 | 1.312 | | hypothetical protein |  |
| gene-SI65_00620 | 1590 | 1.312 | | MFS-type transporter YusP |  |
| gene-SI65_01474 | 849 | 1.311 | | Ser-Thr-rich glycosyl-phosphatidyl-inositol-anchored membrane family |  |
| gene-SI65_00662 | 2115 | 1.310 | | Fungal specific transcription factor domain |  |
| gene-SI65_08191 | 933 | 1.310 | | hypothetical protein |  |
| gene-SI65_01133 | 1032 | 1.304 | | hypothetical protein |  |
| gene-SI65_06283 | 1458 | 1.298 | | RNA recognition motif. (a.k.a. RRM, RBD, or RNP domain) |  |
| gene-SI65_08732 | 918 | 1.298 | | Cis-2,3-dihydrobiphenyl-2,3-diol dehydrogenase |  |
| gene-SI65_08662 | 903 | 1.295 | | Mono- and diacylglycerol lipase |  |
| gene-SI65_07607 | 783 | 1.293 | | hypothetical protein |  |
| gene-SI65_02167 | 780 | 1.290 | | Beta-ketoacyl-ACP reductase |  |
| novel.54 | 1842 | 1.288 | | Thiamine pyrophosphate enzyme, central domain |  |
| gene-SI65_00205 | 1488 | 1.288 | | Quinidine resistance protein 2 |  |
| gene-SI65_07731 | 207 | 1.284 | | Antifungal peptide |  |
| gene-SI65_02149 | 3501 | 1.283 | | Transposon Tf2-1 poly protein |  |
| gene-SI65_08538 | 2868 | 1.282 | | Meiosis protein SPO22/ZIP4 like |  |
| gene-SI65_05703 | 1056 | 1.276 | | hypothetical protein |  |
| gene-SI65_04214 | 765 | 1.27 | | Cytochrome P450 |  |
| gene-SI65_03740 | 1344 | 1.273 | | Nonribosomal peptide synthetase 8 |  |
| novel.327 | 1458 | 1.271 | | unknown |  |
| gene-SI65_06555 | 984 | 1.268 | | Inositol oxygenase 1 |  |
| gene-SI65_10284 | 735 | 1.268 | | Ctr copper transporter family |  |
| gene-SI65_04684 | 1362 | 1.265 | | hypothetical protein |  |
| gene-SI65_07062 | 1701 | 1.263 | | Delta-1-pyrroline-5-carboxylate dehydrogenase |  |
| gene-SI65_06482 | 1908 | 1.259 | | hypothetical protein |  |
| novel.451 | 1448 | 1.259 | | unknown |  |
| gene-SI65_06747 | 894 | 1.257 | | Full=Acyl-protein thioesterase 1 |  |
| gene-SI65_07266 | 327 | 1.255 | | hypothetical protein |  |
| gene-SI65_04220 | 642 | 1.252 | | hypothetical protein |  |
| gene-SI65_03603 | 1188 | 1.246 | | Taurine catabolism dioxygenase TauD, TfdA family |  |
| gene-SI65_03171 | 3192 | 1.245 | | NACHT domain |  |
| gene-SI65_09436 | 4455 | 1.242 | | Ankyrin repeats (3 copies) |  |
| gene-SI65_08857 | 996 | 1.240 | | hypothetical protein |  |
| novel.377 | 803 | 1.235 | | unknown |  |
| gene-SI65_01558 | 738 | 1.232 | | Xyloglucan-specific endo-beta-1,4-glucanase A |  |
| gene-SI65_04605 | 1173 | 1.23 | | hypothetical protein |  |
| gene-SI65_08405 | 3120 | 1.228 | | Non-canonical non-ribosomal peptide synthetase FUB8 |  |
| gene-SI65_03333 | 711 | 1.226 | | Phosphotransferase enzyme family |  |
| gene-SI65_05420 | 1110 | 1.224 | | Ankyrin repeats (3 copies) |  |
| gene-SI65_04662 | 396 | 1.217 | | Guanyl-specific ribonuclease T1 |  |
| novel.118 | 445 | 1.216 | | unknown |  |
| gene-SI65_01485 | 1026 | 1.210 | | Sugar transporter STL1 |  |
| gene-SI65_07455 | 483 | 1.207 | | hypothetical protein |  |
| gene-SI65_10243 | 1854 | 1.205 | | Phenol 2-monooxygenase |  |
| gene-SI65_10278 | 786 | 1.204 | | Quinate dehydrogenase |  |
| gene-SI65_06020 | 780 | 1.201 | | hypothetical protein |  |
| novel.309 | 1050 | 1.197 | | Thioesterase superfamily |  |
| gene-SI65_05462 | 1464 | 1.197 | | hypothetical protein |  |
| novel.409 | 557 | 1.195 | | unknown |  |
| novel.407 | 1469 | 1.195 | | unknown |  |
| gene-SI65_07302 | 264 | 1.1942 | | hypothetical protein |  |
| gene-SI65_01297 | 708 | 1.191 | | Acetyltransferase (GNAT) family |  |
| gene-SI65_05914 | 981 | 1.188 | | Arginase |  |
| gene-SI65_02152 | 330 | 1.185 | | hypothetical protein |  |
| novel.2 | 1589 | 1.184 | | unknown |  |
| gene-SI65_07870 | 1044 | 1.183 | | Quinate dehydrogenase |  |
| gene-SI65_08477 | 3177 | 1.182 | | Regulatory protein AfsR |  |
| gene-SI65_00619 | 1443 | 1.180 | | Cytochrome b2 |  |
| gene-SI65_10317 | 768 | 1.177 | | hypothetical protein |  |
| novel.249 | 1045 | 1.177 | | unknown |  |
| gene-SI65_07774 | 1035 | 1.176 | | Polysaccharide monooxygenase Cel61a |  |
| gene-SI65_10113 | 1107 | 1.176 | | Endopolygalacturonase I |  |
| gene-SI65_00932 | 1002 | 1.175 | | hypothetical protein |  |
| gene-SI65_08277 | 1137 | 1.166 | | Probable glycosidase crf1 |  |
| gene-SI65_00042 | 1713 | 1.157 | | Low-affinity glucose transporter HXT3 |  |
| gene-SI65_08088 | 7299 | 1.156 | | Cell wall alpha-1,3-glucan synthase ags1 |  |
| gene-SI65_07782 | 753 | 1.154 | | Alpha N-terminal protein methyltransferase 1 |  |
| gene-SI65_02014 | 318 | 1.154 | | 60S ribosomal protein L36 |  |
| novel.354 | 1258 | 1.152 | | -DDE superfamily endonuclease |  |
| gene-SI65_09867 | 345 | 1.152 | | Glycine-rich RNA-binding protein 2, |  |
| gene-SI65_04113 | 654 | 1.151 | | hypothetical protein |  |
| gene-SI65_02862 | 1068 | 1.149 | | S-(hydroxymethyl)glutathione dehydrogenase |  |
| gene-SI65_01050 | 195 | 1.145 | | hypothetical protein |  |
| gene-SI65_10149 | 2250 | 1.145 | | putative alpha-galactosidase C |  |
| gene-SI65_01458 | 777 | 1.144 | | Oxidoreductase AflX |  |
| gene-SI65_06949 | 1935 | 1.143 | | Oxygen-dependent choline dehydrogenase |  |
| gene-SI65_03881 | 1533 | 1.139 | | Methylenomycin A resistance protein |  |
| gene-SI65_05022 | 3114 | 1.136 | | Psi-producing oxygenase A |  |
| gene-SI65_04324 | 396 | 1.129 | | hypothetical protein |  |
| gene-SI65_06590 | 1302 | 1.128 | | Uncharacterized oxidoreductase |  |
| gene-SI65_09809 | 1293 | 1.127 | | hypothetical protein |  |
| gene-SI65_04391 | 672 | 1.126 | | FAD-linked oxidoreductase patO |  |
| gene-SI65_06016 | 699 | 1.126 | | hypothetical protein |  |
| gene-SI65_03956 | 216 | 1.125 | | hypothetical protein |  |
| novel.421 | 586 | 1.121 | | unknown |  |
| gene-SI65_05728 | 885 | 1.121 | | Protein CCC1 |  |
| novel.517 | 1503 | 1.120 | | unknown |  |
| novel.425 | 1316 | 1.120 | | unknown |  |
| gene-SI65_00924 | 429 | 1.118 | | Methylated-DNA--protein-cysteine methyltransferase |  |
| gene-SI65_07371 | 1887 | 1.111 | | 3-(3-hydroxy-phenyl)propionate/3-hydroxycinnamic acid hydroxylase |  |
| gene-SI65_05628 | 1095 | 1.110 | | Cytochrome P450 |  |
| gene-SI65_00885 | 774 | 1.108 | | hypothetical protein |  |
| novel.452 | 1593 | 1.107 | | unknown |  |
| novel.209 | 1786 | 1.107 | | helix-turn-helix, Psq domain |  |
| gene-SI65_07961 | 1008 | 1.105 | | Probable phosphoglycerate mutase ARB_03491 |  |
| gene-SI65_04433 | 726 | 1.104 | | Uncharacterized protein n |  |
| novel.192 | 1504 | 1.101 | | unknown |  |
| gene-SI65_02367 | 1587 | 1.101 | | Probable rhamnogalacturonate lyase A |  |
| novel.341 | 1720 | 1.099 | | unknown |  |
| gene-SI65_07144 | 1044 | 1.096 | | Alcohol dehydrogenase 2 |  |
| gene-SI65_01656 | 1215 | 1.095 | | Alpha/beta hydrolase family |  |
| gene-SI65_09728 | 3789 | 1.093 | | Copper-transporting ATPase RAN1 |  |
| gene-SI65_06933 | 852 | 1.088 | | Domain of unknown function (DUF3328) |  |
| gene-SI65_07260 | 720 | 1.087 | | Versiconal hemiacetal acetate reductasey |  |
| gene-SI65_01339 | 930 | 1.084 | | hypothetical protein |  |
| gene-SI65_03170 | 768 | 1.084 | | hypothetical protein |  |
| gene-SI65_02485 | 813 | 1.083 | | hypothetical protein |  |
| gene-SI65_05184 | 552 | 1.083 | | GPI-anchored CFEM domain protein B |  |
| gene-SI65_03736 | 927 | 1.082 | | Oxidoreductase FAD-binding domain |  |
| gene-SI65_09418 | 813 | 1.081 | | Protein of unknown function |  |
| gene-SI65_09607 | 1119 | 1.081 | | hypothetical protein |  |
| gene-SI65_05945 | 1062 | 1.080 | | hypothetical protein |  |
| gene-SI65_09540 | 999 | 1.079 | | Ankyrin repeats (3 copies) |  |
| gene-SI65_09493 | 639 | 1.074 | | Glutathione S-transferase-like protein gedE |  |
| gene-SI65_01250 | 849 | 1.074 | | Ser-Thr-rich glycosyl-phosphatidyl-inositol-anchored membrane family |  |
| gene-SI65_05329 | 1905 | 1.072 | | Ferric reductase transmembrane component 3 |  |
| gene-SI65_07761 | 1020 | 1.072 | | Putative cyclase |  |
| gene-SI65_04931 | 1248 | 1.067 | | Endo-1,3-alpha-glucanase |  |
| gene-SI65_04114 | 831 | 1.066 | | Ankyrin repeats (3 copies) |  |
| gene-SI65_04037 | 432 | 1.064 | | hypothetical protein |  |
| gene-SI65_09305 | 1233 | 1.063 | | N-Acetylglucosaminyltransferase-IV (GnT-IV) conserved region |  |
| gene-SI65_07952 | 435 | 1.060 | | hypothetical protein |  |
| gene-SI65_08025 | 1995 | 1.060 | | Putative inorganic phosphate transporter C1683.01 |  |
| gene-SI65_02508 | 1080 | 1.059 | | DDE superfamily endonuclease |  |
| gene-SI65_10210 | 2007 | 1.058 | | hypothetical protein |  |
| novel.485 | 2440 | 1.056 | | unknown |  |
| novel.505 | 1659 | 1.053 | | unknown |  |
| gene-SI65_00489 | 474 | 1.053 | | 40S ribosomal protein S10-B |  |
| novel.362 | 3048 | 1.050 | | unknown |  |
| gene-SI65_01047 | 567 | 1.049 | | hypothetical protein |  |
| gene-SI65_08939 | 1080 | 1.048 | | Sodium/calcium exchanger protein |  |
| gene-SI65_05990 | 3198 | 1.044 | | Phosphoglycerate mutase-like protein; S |  |
| gene-SI65_02815 | 240 | 1.044 | | Cx9C motif-containing protein 4 |  |
| gene-SI65_06937 | 831 | 1.042 | | hypothetical protein |  |
| gene-SI65_03096 | 906 | 1.041 | | hypothetical protein |  |
| gene-SI65_01296 | 429 | 1.038 | | hypothetical protein |  |
| gene-SI65_05867 | 2706 | 1.036 | | Cell cycle control protein ago1; |  |
| gene-SI65_09541 | 420 | 1.033 | | hypothetical protein |  |
| gene-SI65_05817 | 924 | 1.032 | | Haemolysin-III related |  |
| novel.448 | 331 | 1.031 | | unknown |  |
| gene-SI65_02279 | 1341 | 1.030 | | Secretory lipase |  |
| gene-SI65_04365 | 858 | 1.030 | | Alcohol dehydrogenase GroES-like domain |  |
| gene-SI65_10092 | 957 | 1.028 | | Dioxygenase |  |
| gene-SI65_04382 | 375 | 1.024 | | hypothetical protein |  |
| novel.104 | 1030 | 1.023 | | unknown |  |
| gene-SI65_05768 | 5337 | 1.023 | | transacylase in aflatoxin biosynthesis |  |
| novel.370 | 2581 | 1.018 | | unknown |  |
| gene-SI65_02097 | 381 | 1.010 | | Mitochondrial pyruvate carrier 1 |  |
| gene-SI65_05827 | 1935 | 1.000 | | hypothetical protein |  |
| gene-SI65_09053 | 1461 | 1.000 | | Ankyrin repeats (3 copies) |  |
| gene-SI65_07132 | 2073 | 1.004 | | Glycosyl hydrolases family 15 |  |
| gene-SI65_05851 | 1743 | 1.003 | | Ferric reductase like transmembrane component |  |
| gene-SI65_10067 | 318 | 1.000 | | hypothetical protein |  |
| gene-SI65_05071 | 3489 | 1.000 | | Adenine phosphoribosyltransferase; |  |
| gene-SI65_02129 | 936 | 1.000 | | hypothetical protein |  |
| gene-SI65_09196 | 936 | -1.000 | | Acetyltransferase (GNAT) domain | |
| gene-SI65_04765 | 1596 | -1.000 | | Amino-acid permease BAT1permease | |
| gene-SI65_05707 | 1650 | -1.000 | | Acetamidase | |
| gene-SI65_01206 | 273 | -1.000 | | hypothetical protein | |
| gene-SI65_04799 | 1158 | -1.000 | | Malic acid transport protein | |
| gene-SI65_00417 | 1629 | -1.008 | | MFS antiporter QDR3 | |
| gene-SI65_02585 | 2928 | -1.010 | | hypothetical protein | |
| gene-SI65_02513 | 2388 | -1.011 | | Receptor-type tyrosine-protein phosphatase V | |
| gene-SI65_04291 | 945 | -1.012 | | Methionine adenosyltransferase 2 subunit beta | |
| gene-SI65_07101 | 1407 | -1.013 | | Inositol phosphosphingolipids phospholipase C | |
| gene-SI65_07431 | 258 | -1.016 | | hypothetical protein | |
| gene-SI65_04858 | 1641 | -1.018 | | Choline oxidase | |
| gene-SI65_03783 | 1974 | -1.020 | | Probable endo-1,3(4)-beta-glucanase AFLA_105200 | |
| gene-SI65_03190 | 3594 | -1.020 | | Ras guanine nucleotide exchange factor A | |
| gene-SI65_05939 | 1566 | -1.021 | | hypothetical protein | |
| gene-SI65_04731 | 1842 | -1.023 | | Putative 2-hydroxyacyl-CoA lyase | |
| gene-SI65_09227 | 4530 | -1.023 | | Pumilio domain-containing protein C6G9.14 | |
| gene-SI65_01714 | 3300 | -1.024 | | hypothetical protein | |
| gene-SI65_02348 | 480 | -1.024 | | FAS1 domain-containing protein AFUA_8G05360 | |
| gene-SI65_00383 | 1746 | -1.025 | | Developmental regulatory protein wetA | |
| gene-SI65_00667 | 1044 | -1.028 | | Protein TOXD | |
| gene-SI65_04492 | 453 | -1.030 | | Protein of unknown function (DUF3317) | |
| gene-SI65_07522 | 3732 | -1.032 | | Cortical actin cytoskeleton protein asp1superfamily (branch 2) | |
| gene-SI65_10188 | 906 | -1.032 | | Probable formate transporter | |
| gene-SI65_09901 | 1164 | -1.033 | | 2-oxoisovalerate dehydrogenase subunit beta 1 | |
| gene-SI65_00886 | 1212 | -1.035 | | Mannose-6-phosphate isomerase | |
| gene-SI65_02987 | 1815 | -1.035 | | SH3 domain-containing protein PJ696.02 | |
| gene-SI65_08460 | 1875 | -1.039 | | hypothetical protein | |
| gene-SI65_07873 | 1275 | -1.041 | | Ethanolamine kinase 1 | |
| gene-SI65_02322 | 3480 | -1.041 | | Chitin synthase D | |
| gene-SI65_08585 | 1245 | -1.042 | | hypothetical protein | |
| novel.441 | 658 | -1.043 | | Unknown | |
| gene-SI65_03722 | 8706 | -1.043 | | Highly reducing polyketide synthase azaB | |
| gene-SI65_05888 | 1257 | -1.045 | | Ribonuclease T2-like protein | |
| gene-SI65_05132 | 2748 | -1.045 | | DENN (AEX-3) domain | |
| gene-SI65_04603 | 774 | -1.046 | | hypothetical protein | |
| novel.63 | 1066 | -1.046 | | PF00098:Zinc knuckle | |
| gene-SI65_05198 | 243 | -1.048 | | hypothetical protein | |
| gene-SI65_01329 | 342 | -1.048 | | hypothetical protein | |
| gene-SI65_03177 | 1377 | -1.051 | | Non-specific lipid-transfer protein | |
| gene-SI65_03258 | 1854 | -1.052 | | Protein SOK1 | |
| gene-SI65_05857 | 2496 | -1.055 | | Probable glutaminase A | |
| gene-SI65_03429 | 1269 | -1.056 | | Indoleamine 2,3-dioxygenase | |
| gene-SI65_08133 | 3021 | -1.059 | | Uncharacterized MscS family protein | |
| gene-SI65_04287 | 2478 | -1.061 | | Low-affinity potassium transport protein | |
| gene-SI65_08093 | 2592 | -1.061 | | hypothetical protein | |
| gene-SI65_05754 | 1317 | -1.063 | | RNA polymerase II transcription elongation factor | |
| gene-SI65_10199 | 2634 | -1.064 | | Nitrogen regulatory protein areA | |
| gene-SI65_04544 | 1893 | -1.067 | | G2/mitotic-specific cyclin-4 | |
| gene-SI65_09047 | 1413 | -1.068 | | hypothetical protein | |
| gene-SI65_01641 | 1341 | -1.068 | | 6-methylsalicylic acid decarboxylase atA | |
| gene-SI65_05934 | 3837 | -1.075 | | Autophagy-related protein 11 | |
| gene-SI65_04516 | 498 | -1.075 | | hypothetical protein | |
| gene-SI65_04564 | 2910 | -1.076 | | Phosphatidylethanolamine N-methyltransferase | |
| gene-SI65_00673 | 1401 | -1.076 | | Uncharacterized transporter C417.10 | |
| gene-SI65_06733 | 2031 | -1.077 | | Dual specificity protein phosphatase PPS1 | |
| gene-SI65_00955 | 1494 | -1.078 | | Uncharacterized glycosyl hydrolase YBR056W | |
| gene-SI65_06543 | 1104 | -1.079 | | ATP-dependent (S)-NAD(P)H-hydrate dehydratase | |
| gene-SI65_04244 | 1614 | -1.079 | | Uncharacterized MFS-type transporter SPBC409.08 | |
| gene-SI65_00151 | 633 | -1.080 | | hypothetical protein | |
| gene-SI65_06284 | 4764 | -1.080 | | Mediator of RNA polymerase II transcription subunit 12 | |
| gene-SI65_08961 | 1680 | -1.086 | | 4-coumarate--CoA ligase-like 1 | |
| gene-SI65_08824 | 1062 | -1.086 | | hypothetical protein | |
| gene-SI65_00548 | 3663 | -1.088 | | Integrase core domain | |
| gene-SI65_02424 | 1047 | -1.090 | | Mitochondrial 18 KDa protein (MTP18) | |
| gene-SI65_07700 | 2139 | -1.094 | | ATP-binding cassette sub-family D member 2 | |
| gene-SI65_00902 | 1722 | -1.095 | | Fusaric acid biosynthesis protein 11 | |
| gene-SI65_03262 | 690 | -1.095 | | hypothetical protein | |
| gene-SI65_09980 | 1440 | -1.097 | | hypothetical protein | |
| gene-SI65_04657 | 591 | -1.097 | | hypothetical protein | |
| gene-SI65_09484 | 1344 | -1.099 | | Homogentisate 1,2-dioxygenase | |
| gene-SI65_07386 | 2955 | -1.100 | | Uncharacterized secreted protein ARB_04696 | |
| gene-SI65_05195 | 1488 | -1.100 | | hypothetical protein | |
| gene-SI65_00301 | 1656 | -1.104 | | Pleckstrin homology domain | |
| gene-SI65_02289 | 663 | -1.106 | | Domain of unknown function (DUF202) | |
| gene-SI65_05199 | 1065 | -1.106 | | Rho1 guanine nucleotide exchange factor 1 | |
| gene-SI65_05645 | 4587 | -1.106 | | fungal STAND N-terminal Goodbye domain | |
| gene-SI65_04929 | 1074 | -1.108 | | hypothetical protein | |
| novel.304 | 1232 | -1.109 | | Unknown | |
| gene-SI65_07703 | 3546 | -1.110 | | hypothetical protein | |
| gene-SI65_02909 | 543 | -1.111 | | Uncharacterized conserved protein | |
| gene-SI65_00369 | 1077 | -1.111 | | Glutamine synthetase | |
| gene-SI65_03990 | 717 | -1.119 | | Syntaxin-10 | |
| gene-SI65_05650 | 4122 | -1.123 | | Sterol 3-beta-glucosyltransferase | |
| gene-SI65_05822 | 1053 | -1.126 | | Transcription initiation factor IIB | |
| gene-SI65_05437 | 2751 | -1.131 | | Protein SPA2 | |
| gene-SI65_09738 | 1410 | -1.131 | | Uncharacterized transporter C1683.12 | |
| gene-SI65_06445 | 708 | -1.132 | | LysM domain-containing protein ARB_05157 | |
| gene-SI65_07327 | 1056 | -1.135 | | Alcohol dehydrogenase | |
| gene-SI65_02906 | 1152 | -1.138 | | Allantoicase | |
| gene-SI65_07821 | 1971 | -1.139 | | Meiotically up-regulated gene 80 protein | |
| gene-SI65_00414 | 2208 | -1.140 | | Neutral trehalase | |
| gene-SI65_06562 | 1467 | -1.142 | | L-pipecolate oxidase | |
| gene-SI65_02813 | 648 | -1.145 | | hypothetical protein | |
| gene-SI65_04471 | 1467 | -1.146 | | Peroxisome assembly protein 12 n | |
| gene-SI65_07882 | 1992 | -1.149 | | Endopolyphosphatase | |
| gene-SI65_06214 | 5295 | -1.152 | | Phospholipase D1 | |
| gene-SI65_05157 | 1539 | -1.153 | | Long-chain specific acyl-CoA dehydrogenase | |
| gene-SI65_00859 | 1098 | -1.157 | | hypothetical protein | |
| gene-SI65_05283 | 1812 | -1.159 | | Efflux pump FUBT | |
| gene-SI65_07425 | 2049 | -1.160 | | Transcriptional activator protein acu-15 | |
| gene-SI65_06169 | 1317 | -1.167 | | Glycosyltransferase like family 2 | |
| gene-SI65_09736 | 1851 | -1.167 | | Dehydrogenase patE | |
| gene-SI65_08963 | 489 | -1.169 | | putative peroxiredoxin pmp20 | |
| gene-SI65_05708 | 921 | -1.169 | | Meiotically up-regulated gene 14 protein | |
| gene-SI65_06838 | 1518 | -1.171 | | hypothetical protein | |
| gene-SI65_05828 | 1818 | -1.172 | | L-lysine 6-monooxygenase (NADPH-requiring) | |
| gene-SI65_07388 | 2934 | -1.176 | | hypothetical protein | |
| gene-SI65_08943 | 1695 | -1.176 | | Protein phosphatase 2C homolog 1 | |
| gene-SI65_03813 | 822 | -1.178 | | Domain of unknown function (DUF4336) | |
| gene-SI65_09695 | 774 | -1.180 | | short chain dehydrogenase | |
| gene-SI65_06139 | 984 | -1.180 | | Hydroxymethylglutaryl-CoA lyase | |
| gene-SI65_05732 | 1689 | -1.181 | | Glycogen recognition site of AMP-activated protein kinase | |
| gene-SI65_03953 | 1329 | -1.181 | | Questin oxidase | |
| gene-SI65_07622 | 2247 | -1.181 | | TBC1 domain family member 14 | |
| gene-SI65_01318 | 7365 | -1.188 | | 1-phosphatidylinositol 3-phosphate 5-kinase fab1 | |
| gene-SI65_03074 | 2226 | -1.194 | | hypothetical protein | |
| gene-SI65_01360 | 5199 | -1.196 | | Rap1-interacting factor 1 N terminal | |
| gene-SI65_09829 | 3441 | -1.200 | | Protein efr3 | |
| gene-SI65_07443 | 1224 | -1.209 | | Sterol 24-C-methyltransferase erg6 | |
| gene-SI65_05889 | 2325 | -1.209 | | Cell wall transcription factor ACE2 | |
| gene-SI65_03989 | 1062 | -1.211 | | hypothetical protein | |
| gene-SI65_01091 | 3747 | -1.211 | | Serine/threonine-protein kinase ssp1 | |
| gene-SI65_03364 | 3747 | -1.211 | | hypothetical protein | |
| gene-SI65_00475 | 942 | -1.213 | | L-carnitine dehydrogenase | |
| gene-SI65_02941 | 1473 | -1.215 | | Carboxypeptidase Y homolog ARB_05721 | |
| gene-SI65_08997 | 1410 | -1.215 | | hypothetical protein | |
| gene-SI65_04904 | 2154 | -1.221 | | Phosphorus acquisition-controlling protein | |
| gene-SI65_00540 | 1728 | -1.223 | | Adenosine deaminase CECR1-A | |
| gene-SI65_05316 | 1428 | -1.223 | | Periostin | |
| gene-SI65_08370 | 1806 | -1.226 | | Laccase abr2 | |
| gene-SI65_05293 | 747 | -1.230 | | hypothetical protein | |
| gene-SI65_01188 | 1857 | -1.232 | | UPF0061 protein C20G4.05c | |
| novel.108 | 610 | -1.233 | | Unknown | |
| gene-SI65_06656 | 1818 | -1.234 | | Multidrug resistance protein fnx1 | |
| gene-SI65_08047 | 252 | -1.23 | | hypothetical protein | |
| gene-SI65_03896 | 2619 | -1.236 | | hypothetical protein | |
| gene-SI65_00183 | 1452 | -1.236 | | Zinc knuckle | |
| gene-SI65_02328 | 1575 | -1.239 | | Acetyl-CoA hydrolase n | |
| gene-SI65_04673 | 2187 | -1.243 | | Vacuolar calcium ion transporter | |
| gene-SI65_05266 | 1992 | -1.246 | | Arylsulfotransferase (ASST) | |
| gene-SI65_07886 | 927 | -1.246 | | Glucan 1,3-beta-glucosidase ARB_02797 | |
| gene-SI65_04835 | 3450 | -1.247 | | hypothetical protein | |
| gene-SI65_10111 | 1230 | -1.251 | | Peptidase M20 domain-containing protein 2 | |
| gene-SI65_05574 | 936 | -1.254 | | Protein-lysine N-methyltransferase EFM4 | |
| gene-SI65_02676 | 1350 | -1.257 | | Adenosylhomocysteinase | |
| gene-SI65_00572 | 1293 | -1.258 | | hypothetical protein | |
| gene-SI65_03849 | 915 | -1.260 | | WSC domain | |
| gene-SI65_04347 | 1635 | -1.261 | | Heterokaryon incompatibility protein (HET) | |
| gene-SI65_06728 | 1161 | -1.270 | | P-type cation-transporting ATPase | |
| gene-SI65_05265 | 858 | -1.272 | | Sulfotransferase family | |
| gene-SI65_03441 | 924 | -1.273 | | Uncharacterized urease accessory protein UreF-like | |
| gene-SI65_08501 | 579 | -1.274 | | Glu-tRNAGln amidotransferase C subunit | |
| gene-SI65_04266 | 1017 | -1.276 | | hypothetical protein | |
| gene-SI65_01702 | 990 | -1.278 | | hypothetical protein | |
| gene-SI65_06170 | 1023 | -1.279 | | hypothetical protein | |
| gene-SI65_09450 | 180 | -1.281 | | hypothetical protein | |
| gene-SI65_01647 | 3231 | -1.285 | | 3',5'-cyclic-nucleotide phosphodiesterase regA | |
| gene-SI65_09354 | 273 | -1.288 | | hypothetical protein | |
| gene-SI65_03125 | 603 | -1.290 | | hypothetical protein | |
| gene-SI65_04319 | 3369 | -1.294 | | Putative zinc protease mug138 | |
| gene-SI65_04722 | 546 | -1.296 | | Carboxypeptidase Y inhibitor | |
| gene-SI65_09147 | 2610 | -1.296 | | Response regulator mcs4 | |
| gene-SI65_06648 | 2142 | -1.298 | | Golgi apyrase | |
| gene-SI65_03209 | 1146 | -1.299 | | hypothetical protein | |
| gene-SI65_00418 | 3027 | -1.301 | | hypothetical protein | |
| gene-SI65_04800 | 741 | -1.305 | | Co-chaperone protein HscB homolog | |
| gene-SI65_02810 | 1164 | -1.306 | | S-adenosylmethionine synthase | |
| gene-SI65_00413 | 1617 | -1.306 | | Isocitrate lyase | |
| gene-SI65_00060 | 2169 | -1.306 | | Phenylalanine ammonia-lyase | |
| gene-SI65_10143 | 2112 | -1.308 | | Transcriptional activator protein DAL81 | |
| gene-SI65_02363 | 1653 | -1.309 | | Probable guanine deaminase | |
| gene-SI65_02171 | 2712 | -1.310 | | Berberine bridge enzyme-like 26 | |
| gene-SI65_02608 | 2286 | -1.317 | | hypothetical protein | |
| gene-SI65_07088 | 1575 | -1.317 | | Cytochrome P450 52A4 | |
| gene-SI65_09470 | 1431 | -1.317 | | hypothetical protein | |
| gene-SI65_05141 | 1233 | -1.319 | | hypothetical protein | |
| gene-SI65_02829 | 1521 | -1.325 | | Flavin containing amine oxidoreductase | |
| gene-SI65_04950 | 1011 | -1.327 | | AB hydrolase superfamily protein C1039.03 | |
| gene-SI65_07532 | 2841 | -1.327 | | Kinesin-related protein 11 | |
| gene-SI65_06076 | 1563 | -1.329 | | putative UTP--glucose-1-phosphate uridylyltransferase | |
| gene-SI65_09616 | 1551 | -1.329 | | hypothetical protein | |
| gene-SI65_02939 | 2016 | -1.330 | | Fungal Zn(2)-Cys(6) binuclear cluster domain | |
| gene-SI65_07689 | 1845 | -1.335 | | hypothetical protein | |
| gene-SI65_07123 | 2004 | -1.340 | | hypothetical protein | |
| gene-SI65_04502 | 1365 | -1.346 | | Uncharacterized MFS-type transporter C18.02 | |
| gene-SI65_02461 | 1641 | -1.347 | | Putative amidase C550.07 | |
| novel.438 | 1343 | -1.351 | | Unknown | |
| gene-SI65_08572 | 849 | -1.358 | | hypothetical protein | |
| gene-SI65_00768 | 1368 | -1.3608 | | Mitochondrial calcium uniporter | |
| gene-SI65_02436 | 1290 | -1.361 | | hypothetical protein | |
| gene-SI65_02673 | 885 | -1.364 | | L-xylulose reductase | |
| gene-SI65_09667 | 2013 | -1.369 | | Protein kinase domain | |
| gene-SI65_04580 | 1293 | -1.370 | | L-saccharopine oxidase | |
| gene-SI65_09649 | 1344 | -1.371 | | Protein EPD1 | |
| gene-SI65_06222 | 4230 | -1.374 | | PH and SEC7 domain-containing protein C11E3.11c | |
| gene-SI65_09111 | 510 | -1.378 | | hypothetical protein | |
| gene-SI65_09424 | 384 | -1.379 | | hypothetical protein | |
| gene-SI65_04795 | 2064 | -1.382 | | CAP-Gly domain | |
| gene-SI65_06584 | 495 | -1.383 | | hypothetical protein | |
| gene-SI65_01165 | 1575 | -1.383 | | Probable aspartokinase | |
| gene-SI65_05365 | 1560 | -1.395 | | Uncharacterized transporter C757.13 | |
| gene-SI65_09793 | 810 | -1.398 | | hypothetical protein | |
| gene-SI65_06503 | 2025 | -1.398 | | Beta-glucan synthesis-associated protein KRE6 | |
| gene-SI65_07412 | 1980 | -1.400 | | Putative hydrolase Mb2248c | |
| gene-SI65_07105 | 873 | -1.401 | | Sphingolipid long chain base-responsive protein LSP1 | |
| gene-SI65_03730 | 1806 | -1.421 | | ABC transporter G family member 11 | |
| gene-SI65_01221 | 2460 | -1.425 | | Mitochondrial escape protein 2 | |
| gene-SI65_08874 | 360 | -1.426 | | hypothetical protein | |
| gene-SI65_01218 | 6381 | -1.431 | | putative glutamate synthase [NADPH] | |
| gene-SI65_01576 | 2514 | -1.437 | | Urease | |
| gene-SI65_06899 | 1542 | -1.443 | | Protein HOL1 | |
| gene-SI65_02538 | 1557 | -1.443 | | 3-hydroxyphenylacetate 6-hydroxylase | |
| gene-SI65_08586 | 1803 | -1.447 | | Phosphatidylinositol 3,4,5-trisphosphate 3-phosphatase and dual-specificity protein phosphatase PTEN | |
| gene-SI65_06982 | 2886 | -1.454 | | AB hydrolase superfamily protein C4A8.06c | |
| gene-SI65_02165 | 1131 | -1.459 | | Gibberellin 2-beta-dioxygenase | |
| gene-SI65_07989 | 528 | -1.462 | | Membrane-associating domain | |
| gene-SI65_01623 | 2556 | -1.465 | | Sulfate permease 2 | |
| gene-SI65_07504 | 1320 | -1.465 | | Transcriptional activator hacA | |
| gene-SI65_08213 | 474 | -1.467 | | Domain of unknown function (DUF4470) | |
| gene-SI65_02460 | 1065 | -1.474 | | Zinc/cadmium resistance protein | |
| gene-SI65_07616 | 3018 | -1.475 | | Uncharacterized protein YJR098C | |
| gene-SI65_00612 | 984 | -1.480 | | hypothetical protein | |
| gene-SI65_09996 | 4386 | -1.480 | | ABC transporter ATP-binding protein/permease PDR18 | |
| gene-SI65_02663 | 237 | -1.488 | | Peptidase inhibitor I78 family | |
| gene-SI65_00102 | 627 | -1.488 | | hypothetical protein | |
| gene-SI65_08776 | 3603 | -1.489 | | Reverse transcriptase (RNA-dependent DNA polymerase) | |
| gene-SI65_03212 | 957 | -1.493 | | Alcohol dehydrogenase | |
| gene-SI65_08826 | 1338 | -1.493 | | Mannan endo-1,6-alpha-mannosidase DCW1 | |
| gene-SI65_03417 | 2691 | -1.502 | | Pumilio homolog 3 | |
| gene-SI65_05380 | 1503 | -1.502 | | Probable mannosyl-oligosaccharide alpha-1,2-mannosidase 1B | |
| gene-SI65_03129 | 1284 | -1.504 | | Uncharacterized protein C19C2.10 | |
| gene-SI65_02476 | 1311 | -1.507 | | Fungal specific transcription factor domain | |
| gene-SI65_08379 | 5262 | -1.510 | | hypothetical protein | |
| gene-SI65_03460 | 1176 | -1.510 | | hypothetical protein | |
| gene-SI65_07772 | 168 | -1.510 | | hypothetical protein | |
| gene-SI65_09799 | 375 | -1.511 | | hypothetical protein | |
| gene-SI65_02927 | 1158 | -1.511 | | Uncharacterized CDP-alcohol phosphatidyltransferase class-I family protein C22A12.08c | |
| gene-SI65_01199 | 879 | -1.514 | | Myb-like DNA-binding domain | |
| gene-SI65_07385 | 582 | -1.518 | | hypothetical protein | |
| gene-SI65_02283 | 1359 | -1.521 | | Oxalate decarboxylase OxdC | |
| novel.99 | 590 | -1.525 | | Unknown | |
| gene-SI65_00820 | 912 | -1.5290 | | Nicotinamide riboside kinase | |
| gene-SI65_09296 | 540 | -1.542 | | Hydrophobic surface binding protein A | |
| gene-SI65_09202 | 552 | -1.545 | | Anthrone oxygenase encC | |
| gene-SI65_06343 | 1161 | -1.545 | | Protein NipSnap homolog 1 | |
| gene-SI65_10249 | 1515 | -1.547 | | Uncharacterized transporter C36.01c | |
| gene-SI65_01258 | 831 | -1.549 | | Glutathione S-transferase U17 | |
| gene-SI65_01398 | 963 | -1.550 | | Sulfotransferase 1C2 | |
| gene-SI65_00300 | 3276 | -1.550 | | Uncharacterized protein C24H6.11c | |
| gene-SI65_00264 | 690 | -1.566 | | Probable gluconokinase | |
| gene-SI65_02949 | 3381 | -1.573 | | Linoleate 10R-lipoxygenase | |
| gene-SI65_04049 | 318 | -1.578 | | hypothetical protein | |
| gene-SI65_09681 | 690 | -1.589 | | hypothetical protein | |
| gene-SI65_05426 | 1440 | -1.592 | | Cytochrome P450 monooxygenase azaI | |
| gene-SI65_02674 | 834 | -1.594 | | Uncharacterized protein y4oV | |
| gene-SI65_03193 | 1380 | -1.596 | | NADP-specific glutamate dehydrogenase | |
| gene-SI65_10028 | 1164 | -1.597 | | Alpha-ketoglutarate-dependent sulfonate dioxygenase | |
| novel.487 | 2219 | -1.599 | | Fungal Zn(2)-Cys(6) binuclear cluster domain | |
| gene-SI65_09605 | 3285 | -1.604 | | Sporulation-specific protein 71 | |
| novel.241 | 1720 | -1.608 | | Unknown | |
| gene-SI65_06419 | 2400 | -1.608 | | Protein of unknown function (DUF3433) | |
| gene-SI65_08090 | 2025 | -1.609 | | Methylenetetrahydrofolate reductase 2 | |
| gene-SI65_03897 | 885 | -1.614 | | L-xylulose reductase | |
| gene-SI65_00618 | 1218 | -1.616 | | putative 4-hydroxyphenylpyruvate dioxygenase 2 | |
| gene-SI65_03294 | 1593 | -1.617 | | Polyamine oxidase | |
| gene-SI65_07477 | 396 | -1.619 | | Rodlet protein | |
| gene-SI65_07712 | 3300 | -1.627 | | Nitrite reductase [NAD(P)H] | |
| gene-SI65_01347 | 990 | -1.630 | | Sulfotransferase 1C2 | |
| gene-SI65_09786 | 846 | -1.630 | | Uncharacterized oxidoreductase YJR096W | |
| gene-SI65_00639 | 2862 | -1.632 | | Serine kinase atg1 | |
| gene-SI65_01393 | 318 | -1.639 | | Thioredoxin | |
| gene-SI65_05873 | 1710 | -1.640 | | Pyruvate decarboxylase | |
| gene-SI65_09463 | 2325 | -1.641 | | putative 5-methyltetrahydropteroyltriglutamate--homocysteine methyltransferase | |
| gene-SI65_01013 | 555 | -1.643 | | hypothetical protein | |
| gene-SI65_08967 | 1443 | -1.646 | | Uncharacterized transporter C1683.12 | |
| gene-SI65_03619 | 1782 | -1.649 | | NADP-dependent malic enzyme | |
| gene-SI65_05158 | 1740 | -1.653 | | Adenosine deaminase CECR1-A | |
| gene-SI65_07918 | 1461 | -1.656 | | Protein HOL1 | |
| novel.345 | 356 | -1.659 | | Unknown | |
| gene-SI65_07332 | 801 | -1.661 | | hypothetical protein | |
| gene-SI65_09944 | 1101 | -1.662 | | hypothetical protein | |
| gene-SI65_02291 | 951 | -1.662 | | Regulator of G protein signaling domain | |
| gene-SI65_02008 | 1131 | -1.667 | | hypothetical protein | |
| gene-SI65_01456 | 1251 | -1.671 | | Probable glucan 1,3-beta-glucosidase A | |
| gene-SI65_09719 | 1608 | -1.676 | | Ent-kaurene oxidase | |
| gene-SI65_04265 | 270 | -1.686 | | hypothetical protein | |
| gene-SI65_09945 | 819 | -1.698 | | Carnitinyl-CoA dehydratase | |
| gene-SI65_03270 | 549 | -1.701 | | hypothetical protein | |
| gene-SI65_09885 | 312 | -1.712 | | Mitochondrial ATPase inhibitor, IATP | |
| gene-SI65_03261 | 1584 | -1.718 | | hypothetical protein | |
| gene-SI65_02357 | 1203 | -1.72 | | D-serine dehydratase | |
| gene-SI65_05669 | 1494 | -1.721 | | Catalase | |
| gene-SI65_01706 | 1263 | -1.726 | | RNA recognition motif | |
| gene-SI65_00652 | 1263 | -1.728 | | Fumarylacetoacetase | |
| gene-SI65_06234 | 2523 | -1.730 | | Ig-like domain from next to BRCA1 gene | |
| gene-SI65_00579 | 1686 | -1.734 | | Glycerol kinase 3 | |
| gene-SI65_07713 | 2589 | -1.741 | | Nitrate reductase [NADPH] | |
| gene-SI65_00059 | 777 | -1.754 | | GPI-anchored CFEM domain protein A | |
| gene-SI65_02916 | 2700 | -1.758 | | TBC domain-containing protein C1778.09 | |
| gene-SI65_01279 | 1236 | -1.769 | | Alpha/beta hydrolase family | |
| gene-SI65_03857 | 930 | -1.778 | | Peptidoglycan deacetylase | |
| gene-SI65_09430 | 1560 | -1.783 | | Dye-decolorizing peroxidase msp1 | |
| gene-SI65_09910 | 1521 | -1.789 | | Uncharacterized MFS-type transporter C530.15c | |
| gene-SI65_02794 | 2367 | -1.790 | | Uncharacterized secreted glycosidase ARB_07629 | |
| gene-SI65_01874 | 1131 | -1.791 | | hypothetical protein | |
| gene-SI65_09797 | 2169 | -1.794 | | Presilphiperfolan-8-beta-ol synthase | |
| gene-SI65_09854 | 1119 | -1.796 | | Mitochondrial phosphate carrier protein 2 | |
| gene-SI65_02060 | 1311 | -1.808 | | L-serine dehydratase | |
| gene-SI65_08968 | 795 | -1.808 | | UPF0317 protein C5H10.01 | |
| gene-SI65_03763 | 1602 | -1.808 | | FAD binding domain | |
| gene-SI65_09994 | 1614 | -1.814 | | COBW domain-containing protein | |
| gene-SI65_00617 | 1494 | -1.824 | | Aromatic amino acid aminotransferase C56E4.03 | |
| gene-SI65_08869 | 2922 | -1.825 | | Probable phospholipas | |
| gene-SI65_10318 | 1380 | -1.831 | | Uncharacterized protein YDR124W | |
| gene-SI65_04508 | 168 | -1.835 | | hypothetical protein | |
| gene-SI65_02942 | 2322 | -1.836 | | Sexual differentiation process protein isp4 | |
| gene-SI65_10198 | 480 | -1.843 | | hypothetical protein | |
| gene-SI65_06790 | 2628 | -1.849 | | hypothetical protein | |
| gene-SI65_06378 | 2067 | -1.850 | | Glutaminase A | |
| gene-SI65_10203 | 1662 | -1.856 | | Probable allantoin permease | |
| gene-SI65_01214 | 2370 | -1.860 | | Oligopeptide transporter 8 | |
| gene-SI65_07398 | 2262 | -1.867 | | Uncharacterized glycosyl hydrolase YIR007W | |
| gene-SI65_04680 | 2559 | -1.868 | | Pheromone-regulated membrane protein 10 | |
| gene-SI65_05238 | 1599 | -1.869 | | Major Facilitator Superfamily | |
| gene-SI65_09357 | 1551 | -1.876 | | Uncharacterized amino-acid permease C15C4.04c | |
| gene-SI65_09972 | 1455 | -1.898 | | hypothetical protein | |
| gene-SI65_10062 | 561 | -1.903 | | hypothetical protein | |
| gene-SI65_06742 | 2808 | -1.903 | | Lon protease homolog 2, peroxisomal | |
| gene-SI65_09733 | 1584 | -1.905 | | Cytochrome P450 monooxygenase yanC | |
| gene-SI65_00444 | 2550 | -1.908 | | Mannosyl-oligosaccharide 1,2-alpha-mannosidase IA | |
| gene-SI65_05839 | 1482 | -1.912 | | Tryprostatin B 6-hydroxylase | |
| gene-SI65_02480 | 1032 | -1.913 | | hypothetical protein | |
| gene-SI65_07250 | 1947 | -1.913 | | Tyrosinase | |
| gene-SI65_05575 | 1461 | -1.920 | | hypothetical protein | |
| gene-SI65_07038 | 741 | -1.922 | | Uncharacterized protein YpgQ | |
| gene-SI65_06561 | 1545 | -1.929 | | Uncharacterized transporter C1039.04 | |
| gene-SI65_06098 | 1101 | -1.936 | | Cytochrome P450 monooxygenase pyr3 | |
| gene-SI65_04754 | 1794 | -1.947 | | Beta-hexosaminidase 1 | |
| gene-SI65_06326 | 1911 | -1.952 | | hypothetical protein | |
| gene-SI65_06720 | 3618 | -1.953 | | Glycerophosphodiester phosphodiesterase GDE1 | |
| gene-SI65_08707 | 1665 | -1.956 | | Ammonium transporter MEP3 | |
| gene-SI65_00462 | 1902 | -1.959 | | Transcription factor prr | |
| gene-SI65_06673 | 7677 | -1.973 | | Non-reducing polyketide synthase andM | |
| gene-SI65_06991 | 648 | -1.973 | | hypothetical protein | |
| gene-SI65_09841 | 2547 | -1.974 | | Pheromone-regulated membrane protein 10 | |
| gene-SI65_07669 | 1656 | -1.98 | | Uracil permease | |
| gene-SI65_10242 | 762 | -2.015 | | Probable NADP-dependent mannitol dehydrogenase [NADP(+)] | |
| gene-SI65_07980 | 2124 | -2.020 | | Sarcolemmal membrane-associated protein | |
| gene-SI65_07786 | 690 | -2.024 | | Protein of unknown function (DUF541) | |
| gene-SI65_04705 | 438 | -2.025 | | hypothetical protein | |
| gene-SI65_07837 | 372 | -2.029 | | hypothetical protein | |
| gene-SI65_04592 | 948 | -2.029 | | Probable NADP-dependent mannitol dehydrogenase | |
| gene-SI65_08978 | 3531 | -2.031 | | Apoptosis inhibitor IAP | |
| gene-SI65_02845 | 1437 | -2.056 | | Ammonium transporter 1 | |
| gene-SI65_00665 | 1005 | -2.057 | | Fungal Zn(2)-Cys(6) binuclear cluster domain | |
| gene-SI65_08619 | 390 | -2.064 | | Lactonase, 7-bladed beta-propeller | |
| gene-SI65_00857 | 3744 | -2.077 | | Meiotically up-regulated gene 190 protein | |
| gene-SI65_02211 | 2922 | -2.077 | | Uncharacterized MscS family protein C1183.11 | |
| gene-SI65_03979 | 1536 | -2.083 | | hypothetical protein | |
| gene-SI65_09933 | 273 | -2.086 | | Conidiation-specific protein 6 | |
| gene-SI65_05951 | 783 | -2.091 | | hypothetical protein | |
| gene-SI65_07296 | 1317 | -2.097 | | Uracil-regulated protein 1 | |
| gene-SI65_07308 | 564 | -2.098 | | Cupin domain | |
| gene-SI65_08475 | 6354 | -2.098 | | Conidial pigment polyketide synthase alb | |
| gene-SI65_09650 | 855 | -2.100 | | hypothetical protein | |
| gene-SI65_06925 | 288 | -2.108 | | hypothetical protein | |
| gene-SI65_09721 | 1182 | -2.117 | | Geranylgeranyl pyrophosphate synthase | |
| gene-SI65_08592 | 1833 | -2.119 | | Efflux pump FUB11 | |
| gene-SI65_02222 | 1146 | -2.119 | | Aldolase vrtJ | |
| gene-SI65_08956 | 1284 | -2.133 | | Salicylate hydroxylase | |
| gene-SI65_00020 | 3840 | -2.134 | | Hydantoinase/oxoprolinase N-terminal region | |
| gene-SI65_00988 | 1479 | -2.138 | | Uncharacterized transporter | |
| novel.248 | 2473 | -2.142 | | Unknown | |
| gene-SI65_08797 | 1224 | -2.148 | | Domain of unknown function (DUF1996) | |
| gene-SI65_05883 | 2208 | -2.148 | | Grainyhead-like protein 2 | |
| gene-SI65_05526 | 1176 | -2.159 | | hypothetical protein | |
| gene-SI65_06889 | 375 | -2.160 | | hypothetical protein | |
| gene-SI65_06392 | 1299 | -2.161 | | Chitotriosidase-118 | |
| gene-SI65_08118 | 1014 | -2.173 | | Trichodiene synthase (TRI5) | |
| gene-SI65_08354 | 1788 | -2.176 | | Uncharacterized membrane protein YCR023C | |
| gene-SI65_08024 | 618 | -2.197 | | hypothetical protein | |
| gene-SI65_08234 | 654 | -2.216 | | hypothetical protein | |
| gene-SI65_00093 | 1293 | -2.225 | | hypothetical protein | |
| gene-SI65_05213 | 2199 | -2.230 | | Transcriptional regulator RPN4 | |
| gene-SI65_03626 | 1413 | -2.234 | | Uncharacterized transporter C417.10 | |
| gene-SI65_02539 | 2151 | -2.237 | | Glycerol-3-phosphate dehydrogenase, mitochondrial | |
| gene-SI65_09563 | 792 | -2.252 | | 2,5-dichloro-2,5-cyclohexadiene-1,4-diol dehydrogenase | |
| gene-SI65_00084 | 519 | -2.261 | | GXWXG protein | |
| gene-SI65_01213 | 1914 | -2.266 | | Heat shock 70 kDa protein | |
| gene-SI65_02221 | 1704 | -2.270 | | hypothetical protein | |
| gene-SI65_07879 | 1251 | -2.282 | | hypothetical protein | |
| gene-SI65_05966 | 816 | -2.282 | | Alpha/beta hydrolase family | |
| gene-SI65_04947 | 1935 | -2.284 | | hypothetical protein | |
| gene-SI65_10155 | 1524 | -2.296 | | Phosphatidylserine decarboxylase | |
| gene-SI65_06344 | 765 | -2.299 | | Pyridoxamine 5'-phosphate oxidase | |
| gene-SI65_04121 | 360 | -2.305 | | Ethanolamine utilisation protein EutQ | |
| gene-SI65_10070 | 891 | -2.308 | | NmrA-like family | |
| gene-SI65_03756 | 1683 | -2.308 | | Cystathionine gamma-synthase | |
| gene-SI65_07180 | 1803 | -2.319 | | Phosphoenolpyruvate carboxykinase [ATP] | |
| gene-SI65_08120 | 3396 | -2.319 | | Full=Acetyl-coenzyme A synthetase | |
| gene-SI65_00627 | 1668 | -2.328 | | Ubiquitin 3 binding protein But2 C-terminal domain | |
| gene-SI65_00360 | 897 | -2.347 | | Beta-lactamase hydrolase-like protein | |
| gene-SI65_05236 | 537 | -2.358 | | hypothetical protein | |
| gene-SI65_02726 | 789 | -2.367 | | Glutathione S-transferase 1 | |
| gene-SI65_04874 | 624 | -2.378 | | Thioredoxin | |
| gene-SI65_00497 | 1788 | -2.382 | | Uncharacterized membrane protein C3B8.06 | |
| gene-SI65_00065 | 537 | -2.397 | | Diacetyl reductase [(S)-acetoin forming] | |
| gene-SI65_08207 | 666 | -2.406 | | hypothetical protein | |
| gene-SI65_06190 | 732 | -2.412 | | Protein-L-isoaspartate(D-aspartate) O-methyltransferase | |
| gene-SI65_07711 | 1542 | -2.418 | | Nitrate transporter | |
| gene-SI65_02564 | 603 | -2.437 | | Methylene-fatty-acyl-phospholipid synthase | |
| gene-SI65_08137 | 1149 | -2.441 | | Norsolorinic acid reductase A | |
| gene-SI65_03629 | 1158 | -2.441 | | hypothetical protein | |
| gene-SI65_03363 | 3423 | -2.444 | | Tetratricopeptide repeat | |
| gene-SI65_07921 | 1239 | -2.445 | | Putative formamidase C869.04 | |
| gene-SI65_03180 | 927 | -2.447 | | Protein of unknown function (DUF3632) | |
| gene-SI65_06239 | 1467 | -2.456 | | Interferon-related developmental regulator (IFRD) | |
| gene-SI65_06409 | 981 | -2.463 | | Protein of unknown function (DUF1275) | |
| gene-SI65_03677 | 1665 | -2.468 | | Dicarboxylic amino acid permease | |
| gene-SI65_06596 | 1746 | -2.494 | | Efflux pump FUS6 | |
| gene-SI65_03232 | 1479 | -2.502 | | Aspartic-type endopeptidase ctsD | |
| gene-SI65_05745 | 1008 | -2.505 | | hypothetical protein | |
| gene-SI65_08135 | 1869 | -2.506 | | hypothetical protein | |
| gene-SI65_08671 | 963 | -2.510 | | Life-span regulatory factor | |
| gene-SI65_04268 | 1344 | -2.535 | | Adenosine deaminase CECR1 | |
| gene-SI65_03265 | 753 | -2.546 | | ATP-dependent bile acid permease | |
| gene-SI65_08567 | 921 | -2.573 | | Mitochondrial phosphate carrier protein 2 | |
| gene-SI65_05145 | 1410 | -2.597 | | hypothetical protein | |
| gene-SI65_09623 | 504 | -2.602 | | hypothetical protein | |
| gene-SI65_06675 | 1002 | -2.625 | | FAD-dependent monooxygenase terD | |
| gene-SI65_10225 | 1746 | -2.634 | | Efflux pump roqT | |
| gene-SI65_04788 | 1593 | -2.647 | | Deoxyribonuclease NucA/NucB | |
| gene-SI65_02761 | 1773 | -2.654 | | Laccase-2 | |
| gene-SI65_05134 | 3324 | -2.665 | | Calcium channel YVC1 | |
| gene-SI65_03267 | 561 | -2.668 | | Canalicular multispecific organic anion transporter 1 | |
| gene-SI65_00246 | 2157 | -2.669 | | hypothetical protein | |
| gene-SI65_04860 | 1017 | -2.670 | | Glucosamine-6-phosphate deaminase | |
| gene-SI65_04290 | 2382 | -2.709 | | hypothetical protein | |
| gene-SI65_08464 | 714 | -2.714 | | Isoprenyl transferase | |
| gene-SI65_01743 | 1812 | -2.751 | | Arrestin (or S-antigen), N-terminal domain | |
| gene-SI65_07377 | 1386 | -2.789 | | hypothetical protein | |
| gene-SI65_09798 | 468 | -2.793 | | hypothetical protein | |
| gene-SI65_09604 | 738 | -2.814 | | Uncharacterized oxidoreductase SSP1627 | |
| gene-SI65_10241 | 1392 | -2.827 | | 3-hydroxybenzoate 6-hydroxylase | |
| gene-SI65_01812 | 1044 | -2.833 | | Putative monooxygenase Rv1533 | |
| gene-SI65_03945 | 6447 | -2.841 | | Conidial yellow pigment biosynthesis polyketide synthase | |
| gene-SI65_09175 | 450 | -2.849 | | hypothetical protein | |
| gene-SI65_09855 | 459 | -2.856 | | Protein of unknown function (DUF3602) | |
| gene-SI65_10183 | 978 | -2.864 | | D-arabinitol 2-dehydrogenase [ribulose-forming] | |
| gene-SI65_05646 | 1515 | -2.903 | | Allantoinase | |
| gene-SI65_04798 | 873 | -2.91 | | hypothetical protein | |
| gene-SI65_02159 | 807 | -2.915 | | hypothetical protein | |
| gene-SI65_08962 | 708 | -2.936 | | DJ-1/PfpI family | |
| gene-SI65_07855 | 870 | -2.939 | | hypothetical protein | |
| gene-SI65_00313 | 2541 | -2.940 | | Protein of unknown function (DUF4449) | |
| gene-SI65_00552 | 1062 | -2.964 | | Fructose-1,6-bisphosphatase | |
| gene-SI65_01346 | 2532 | -2.976 | | Reverse transcriptase (RNA-dependent DNA polymerase) | |
| gene-SI65_06600 | 1644 | -2.994 | | Putative amidase ARB_02965 | |
| gene-SI65_07415 | 747 | -3.006 | | Esterase FUS5; AltName | |
| gene-SI65_10258 | 1071 | -3.026 | | scyllo-inositol 2-dehydrogenase (NAD(+)) | |
| gene-SI65_03268 | 324 | -3.027 | | hypothetical protein | |
| gene-SI65_03924 | 1365 | -3.035 | | Class III chitinase ARB_03514 | |
| gene-SI65_09446 | 891 | -3.062 | | hypothetical protein | |
| gene-SI65_01603 | 2103 | -3.067 | | Probable urea active transporter 1 | |
| gene-SI65_01537 | 522 | -3.082 | | hypothetical protein | |
| gene-SI65_01409 | 2586 | -3.085 | | putative beta-glucosidase A | |
| gene-SI65_09340 | 1767 | -3.107 | | Catalase A | |
| gene-SI65_03269 | 570 | -3.111 | | hypothetical protein | |
| gene-SI65_02343 | 927 | -3.152 | | hypothetical protein | |
| gene-SI65_09889 | 441 | -3.171 | | Translocator protein homolog | |
| gene-SI65_06650 | 1410 | -3.171 | | Ammonium transporter 1 | |
| gene-SI65_00158 | 2040 | -3.178 | | L-amino-acid oxidase | |
| gene-SI65_06676 | 330 | -3.185 | | hypothetical protein | |
| gene-SI65_05670 | 1740 | -3.193 | | Choline oxidase | |
| gene-SI65_08235 | 486 | -3.215 | | hypothetical protein | |
| gene-SI65_09120 | 774 | -3.221 | | hypothetical protein | |
| gene-SI65_08128 | 3459 | -3.224 | | Putative peroxisomal-coenzyme A synthetase | |
| gene-SI65_00682 | 1392 | -3.232 | | Zinc-regulated transporter | |
| gene-SI65_02684 | 759 | -3.245 | | hypothetical protein | |
| gene-SI65_08960 | 1335 | -3.258 | | Beta-cyclopiazonate dehydrogenase | |
| gene-SI65_03275 | 1926 | -3.260 | | Beta-galactosidase | |
| gene-SI65_00749 | 975 | -3.291 | | Homeobox protein homothorax | |
| gene-SI65_00868 | 1725 | -3.303 | | hypothetical protein | |
| gene-SI65_01379 | 1368 | -3.334 | | DNA polymerase Ligase (LigD) | |
| gene-SI65_03291 | 1101 | -3.365 | | Endoglucanase-4 | |
| gene-SI65_09807 | 828 | -3.397 | | Protein of unknown function (DUF2985) | |
| gene-SI65_05229 | 636 | -3.405 | | hypothetical protein | |
| gene-SI65_00122 | 1560 | -3.427 | | Glutamate decarboxylase 1 | |
| gene-SI65_01536 | 639 | -3.429 | | hypothetical protein | |
| gene-SI65_06856 | 1773 | -3.452 | | Dihydroxyacetone kinase 1domain | |
| gene-SI65_09932 | 1599 | -3.465 | | Sugar transporter STL1 | |
| gene-SI65_03266 | 1074 | -3.469 | | ABC transporter ATP-binding protein/permease VMR1 | |
| gene-SI65_02156 | 1929 | -3.483 | | Glucose oxidase | |
| gene-SI65_05545 | 561 | -3.484 | | Protein kinase domain | |
| gene-SI65_05587 | 486 | -3.547 | | Scytalone dehydratase arp1 | |
| gene-SI65_03257 | 1554 | -3.562 | | Carboxypeptidase Y | |
| gene-SI65_08465 | 456 | -3.611 | | hypothetical protein | |
| gene-SI65_08059 | 489 | -3.649 | | putative ribose 5-phosphate isomerase | |
| gene-SI65_01807 | 1335 | -3.655 | | hypothetical protein | |
| gene-SI65_01770 | 2328 | -3.675 | | Ino eighty subunit 1 | |
| gene-SI65_10076 | 891 | -3.690 | | Uncharacterized oxidoreductase MT0954 | |
| gene-SI65_09840 | 339 | -3.695 | | hypothetical protein | |
| gene-SI65_09136 | 735 | -3.706 | | Haloacid dehalogenase-like hydrolase | |
| gene-SI65_02305 | 888 | -3.713 | | hypothetical protein | |
| gene-SI65_01927 | 1590 | -3.722 | | Amino-acid permease BAT1 | |
| gene-SI65_06420 | 2511 | -3.750 | | Glucan 1,3-beta-glucosidase | |
| gene-SI65_09888 | 669 | -3.753 | | Hydrolase FUB4 | |
| gene-SI65_09134 | 831 | -3.781 | | Nudix hydrolase 14, chloroplastic; Short=AtNUDT14 | |
| gene-SI65_08959 | 396 | -3.785 | | hypothetical protein | |
| gene-SI65_07428 | 1389 | -3.844 | | Mannan endo-1,6-alpha-mannosidase DCW1 | |
| gene-SI65_02970 | 1269 | -3.854 | | hypothetical protein | |
| gene-SI65_09782 | 534 | -3.899 | | hypothetical protein | |
| gene-SI65_02252 | 321 | -3.899 | | Fungal hydrophobin | |
| gene-SI65_07342 | 582 | -3.904 | | Caulimovirus viroplasmin | |
| gene-SI65_04851 | 1614 | -3.936 | | Uncharacterized membrane protein YCR023C | |
| gene-SI65_07423 | 417 | -3.944 | | Allergen Aspf 4 | |
| gene-SI65_06585 | 1365 | -3.946 | | hypothetical protein | |
| gene-SI65_03834 | 1131 | -3.954 | | D-arabinitol 2-dehydrogenase | |
| gene-SI65_09118 | 609 | -3.973 | | hypothetical protein | |
| gene-SI65_09482 | 1206 | -3.976 | | putative 4-hydroxyphenylpyruvate dioxygenase 1 | |
| gene-SI65_08250 | 297 | -3.979 | | hypothetical protein | |
| gene-SI65_09735 | 7671 | -4.000 | | Highly reducing polyketide synthase azaB | |
| gene-SI65_07032 | 507 | -4.036 | | hypothetical protein | |
| gene-SI65_05498 | 1227 | -4.057 | | Probable low-specificity L-threonine aldolase | |
| gene-SI65_04064 | 3714 | -4.081 | | Serine/threonine-protein kinase HSL1 | |
| gene-SI65_02778 | 1275 | -4.089 | | C2H2 type master regulator of conidiophore development brlA | |
| gene-SI65_08417 | 348 | -4.185 | | hypothetical protein | |
| gene-SI65_05635 | 738 | -4.188 | | SUR7/PalI family | |
| gene-SI65_09483 | 738 | -4.271 | | hypothetical protein | |
| gene-SI65_08573 | 1380 | -4.274 | | O-methyltransferase tpcA | |
| gene-SI65_08618 | 444 | -4.282 | | hypothetical protein | |
| gene-SI65_09445 | 486 | -4.289 | | hypothetical protein | |
| gene-SI65_02292 | 642 | -4.320 | | hypothetical protein | |
| gene-SI65_05588 | 795 | -4.360 | | Hydroxynaphthalene reductase arp2; | |
| gene-SI65_00131 | 870 | -4.380 | | Domain of unknown function (DUF3328) | |
| gene-SI65_00148 | 348 | -4.402 | | Shwachman-Bodian-Diamond syndrome (SBDS) protein | |
| gene-SI65_04753 | 696 | -4.460 | | RNase H | |
| gene-SI65_02173 | 801 | -4.476 | | putative NADP-dependent mannitol dehydrogenase | |
| gene-SI65_01355 | 597 | -4.488 | | hypothetical protein | |
| gene-SI65_02239 | 990 | -4.525 | | Glycosyl hydrolases family 43 | |
| gene-SI65_06480 | 1557 | -4.528 | | Efflux pump rdc3 | |
| novel.329 | 588 | -4.572 | | UnknoUnknownwn | |
| novel.255 | 618 | -4.602 | | Unknown | |
| gene-SI65_09337 | 2337 | -4.624 | | FAD-NAD(P)-binding | |
| gene-SI65_05590 | 702 | -4.635 | | hypothetical protein | |
| gene-SI65_02779 | 1017 | -4.710 | | Arylsulfatase | |
| gene-SI65_08023 | 1167 | -4.727 | | Mannitol-1-phosphate 5-dehydrogenase | |
| gene-SI65_00055 | 1485 | -4.778 | | MFS antiporter QDR2 | |
| gene-SI65_02459 | 2163 | -4.803 | | Catalase A | |
| gene-SI65_04742 | 771 | -4.901 | | Pal1 cell morphology protein | |
| novel.239 | 805 | -4.93 | | Unknown | |
| gene-SI65_01448 | 279 | -4.977 | | hypothetical protein | |
| gene-SI65_09339 | 1689 | -5.020 | | Efflux pump FUB11 | |
| gene-SI65_05050 | 1992 | -5.049 | | Beta-glucuronidase | |
| gene-SI65_03861 | 915 | -5.086 | | hypothetical protein | |
| gene-SI65_02344 | 579 | -5.131 | | Ser-Thr-rich glycosyl-phosphatidyl-inositol-anchored membrane family | |
| gene-SI65_07617 | 885 | -5.207 | | hypothetical protein | |
| gene-SI65_03918 | 1140 | -5.245 | | hypothetical protein | |
| gene-SI65_06674 | 2634 | -5.309 | | MFS antiporter QDR2 | |
| gene-SI65_08790 | 1305 | -5.399 | | hypothetical protein | |
| gene-SI65_09651 | 1296 | -5.499 | | hypothetical protein | |
| gene-SI65_03961 | 360 | -5.535 | | hypothetical protein | |
| gene-SI65_10255 | 414 | -5.577 | | Rodlet protein | |
| gene-SI65_05589 | 1227 | -5.593 | | Heptaketide hydrolyase ayg1 | |
| gene-SI65_09890 | 2562 | -5.640 | | Pheromone-regulated membrane protein 10 | |
| gene-SI65_03176 | 360 | -5.643 | | hypothetical protein | |
| gene-SI65_01918 | 1593 | -5.646 | | Proline-specific permease | |
| gene-SI65_08640 | 1596 | -5.651 | | 1,3-beta-glucanosyltransferase gel4 | |
| gene-SI65_08474 | 1398 | -5.657 | | Arylsulfotransferase (ASST) | |
| gene-SI65_00764 | 540 | -5.697 | | hypothetical protein | |
| gene-SI65_03776 | 1440 | -5.846 | | hypothetical protein | |
| gene-SI65_07424 | 477 | -6.016 | | hypothetical protein | |
| gene-SI65_00361 | 780 | -6.036 | | Sulfite efflux pump SSU1 | |
| gene-SI65_05591 | 1455 | -6.155 | | Multicopper oxidase abr1 | |
| gene-SI65_04578 | 333 | -6.186 | | hypothetical protein | |
| gene-SI65_06408 | 1833 | -6.388 | | hypothetical protein | |
| gene-SI65_05592 | 1758 | -6.417 | | Laccase abr2 | |
| gene-SI65_05163 | 1218 | -6.454 | | hypothetical protein | |
| gene-SI65_04948 | 558 | -6.454 | | hypothetical protein | |
| gene-SI65_00446 | 1161 | -6.609 | | Probable beta-glucosidase btgE | |
| gene-SI65_00763 | 468 | -6.787 | | Multistep phosphorelay regulator 1 | |
| gene-SI65_02867 | 1518 | -6.805 | | Vitamin B6 transporter TPN1 | |
| gene-SI65_04459 | 336 | -6.949 | | hypothetical protein | |
| gene-SI65_09338 | 315 | -6.954 | | Stress responsive A/B Barrel Domain | |
| gene-SI65_07257 | 759 | -6.974 | | SnoaL-like domain | |
| gene-SI65_03917 | 1212 | -7.067 | | Protein kinase dsk1 | |
| gene-SI65_06672 | 2205 | -7.097 | | Cytochrome P450 monooxygenase mpaDE | |
| gene-SI65_02242 | 1089 | -7.366 | | hypothetical protein | |
| gene-SI65_05586 | 948 | -7.457 | | hypothetical protein | |
| gene-SI65_05422 | 1818 | -7.805 | | Catalase A | |
| gene-SI65_00161 | 1548 | -8.622 | | High-affinity glucose transporter | |
| gene-SI65_01353 | 762 | -8.943 | | Uncharacterized oxidoreductase | |
